# A novel approach towards web browser using the concept of a complex spherical fuzzy soft information

**DOI:** 10.1038/s41598-024-53783-w

**Published:** 2024-04-06

**Authors:** Wenkai Shao, Shoukat Hussain, Sami Ullah Khan, Fuad A. Awwad, Emad A. A. Ismail

**Affiliations:** 1 Department of Mathematical Teaching and Research, Yibin Vocational & and Technical College, Yibin, 644003 Sichuan China; 2 Department of Mathematics, Institute of Numerical Sciences, Gomal University, Dera Ismail Khan, 29050 KPK Pakistan; 3 Department of Quantitative Analysis, College of Business Administration, King Saud University, P.O. Box 71115, 11587 Riyadh, Saudi Arabia

**Keywords:** Engineering, Mathematics and computing

## Abstract

The modern technology is the practical application of scientific knowledge, whether in industry or daily life, for goals or purposes. More quickly than any other technological advancement in human history, digital technologies have advanced. The technology sector is expanding and provides both new educational opportunities and innovative, exciting products. Right now, one of the most widely used and fascinating technologies is the web browser. This article introduced the novel concepts of complex spherical fuzzy soft relations (CSFSRs) by using the Cartesian Product (CP) of two complex spherical fuzzy soft sets (CSFSSs). Additionally, examples are used to clarify various types of relations. Because it discusses all levels of membership, abstinence, and non-membership with multidimensional variables, the CSFSRs have a detailed structure. The CSFSR-based modelling tools developed in this research, which primarily rely on the score function, can be used to choose the best Web browser. The transaction could be as easy as users sharing records via a functional web browser. Finally, the advantages of this suggested structure are illustrated by contrasting it with alternative structures.

## Introduction

A great deal of daily judgments are highly unlikely. It usually occurs when everything is unstable, the future environment is unpredictable, and there is insufficient knowledge about the implications. The decision-maker is not aware of every option that is available to them or the dangers associated with the potential results of each option. In 1965, Zadeh^1^ created the fuzzy set (FS), a ground-breaking mathematical discovery that allowed for the identification and removal of uncertainty. The quality or effectiveness of each element in this collection is indicated by a membership degree, which ranges from 0 to 1. The FS is more important while making decisions as a human. Its applications and the FS theory were put forth by Zimmermann^2^. The foundation of FS theory explains Roberts’^3^. The FSs theory used by Maiers and Sherif^4^ can be used for a variety of problems and fuzzy control methods. Fuzzy relationships (FRs) are an idea put forward by Mendel^5^. The nature of the link was demonstrated by FRs using the membership degree of each element. A positive relationship is indicated if membership is closer to one, whereas a bad relationship is indicated if membership is closer to zero. The structure of FRs is more extensive than that of classical relations. Based on FRs, Yang and Shih^6^ named the cluster analysis. Ramot et al.^7^ nomination of the complex fuzzy set (CFS), which explains membership spanning between unit circles, came after FS. It uses the terms amplitude and phase, which indicate efficacy and duration, respectively, to determine membership. It lessens the possibility of mistakes and ambiguity. CFSs and their applications in multi-class prediction were studied by Li et al.^8^. Logic and complex fuzzy sets should be reviewed systematically, according to Yazdanbakhsh et al.^9^ in addition, he defines complex fuzzy relations (CFRs) as well. The CFRs were established in the future commission market by Khan et al.^10^.

With all of these advancements in human decision-making, choosing the best course of action may still leave people perplexed. There are a lot of unknowns and unclears in this situation. In 1999, Molodtsov^11^ looked into the idea of the soft set (SS), which helps people make better decisions in difficult situations. The products are chosen by SS based on a few factors. Ali et al.^12^ developed special SSs operations. Yang et al. have written a paper on “soft set theory”^13^. The soft relations (SRs) between the CP of SSs were first described by Babitha et al.^14^. An SS theory was used by Maji et al.^15^ to overcome a problem with decision-making. Georgiou et al.^16^ worked on the soft set theory and topology. Some relevant SR properties were researched by Park et al.^17^. The FS and SS were combined to generate the fuzzy soft set (FSS) by Maji et al. By lowering ambiguity in daily decisions, it aids in human decision-making. Maji et al.^18^ investigated the fuzzy soft sets. Gogoi et al.^19^ investigated the potential applications of the FSS theory to numerous problems. FSS was used in decision-making concerns by Kong et al.^20^. By looking at the CP of FSSs, Borah et al.^21^ developed the novel concept of fuzzy soft relations (FSRs). Hayat et al.^22^ Design concept evaluation using soft sets based on acceptable and satisfactory levels. Hayat et al.^23^ proposed the concept of new group-based generalized interval-valued q-rung orthopair fuzzy soft aggregation operators and their applications in sports decision-making problems. Rehman et al.^24^, identification and prioritization of Develops success factors using bipolar complex fuzzy setting with Frank aggregation operators and analytical hierarchy process. Yang et al.^25^ investigated the aggregation and interaction aggregation soft operators on interval-valued q-rung orthopair fuzzy soft environment and application in automation company evaluation. Thirunavukarasu et al.^26^ investigated the innovative concept of complex fuzzy soft sets (CFSSs), in which the problems are handled on a periodic basis and the degree of membership is expressed in complex numbers. A description of CFS and complicated fuzzy logic theory and applications were examined by Tamir et al.^27^.

The concept of an intuitionistic fuzzy set (IFS), which is more expansive than the FS, was introduced by Atanassov^28^. An FSs only covered the membership degree, but an IFS looked at both membership and non-membership degrees. These two values, which fall between [0, 1] and sum, are also contained inside this range. The IFS correlation was discovered by Gerstenkorn and Manko^29^. The concept of the complex intuitionistic fuzzy set (CIFS) was defined by Alkouri et al.^30^. By including complex numbers, the CIFS framework greatly expands the range of membership and non-membership degrees. This inclusion is especially important when dealing with complex and multidimensional situations where traditional representations might not be sufficient. The CIFS offers a more flexible and nuanced method of managing complex scenarios by using complex numbers to define both membership and non-membership degrees. This helps to create a more thorough understanding of uncertainty and imprecision. Furthermore, as suggested by Xu et al.^31^, the IFSS is the result of the synthesis of the principles of the Soft Set (SS) and the Intuitionistic Fuzzy Set (IFS). This hybrid structure combines the sophisticated uncertainty management of IFS with the soft computing principles of SS to further improve modelling capabilities. The inclusion of IFSS in the conversation highlights the ongoing development of methodologies and highlights how dynamic research is when it comes to tackling difficult real-world problems using creative and well-integrated frameworks. The FSS has been expanded into the IFSS. Generalized intuitionistic fuzzy soft sets were introduced by Agarwal et al.^32^ and have used in decision-making. Using the CP of IFSS, Dinda and Samanta^33^ suggested the intuitionistic fuzzy soft relation (IFSR). Kumar and Bajaj^34^ evaluate complex intuitionistic fuzzy soft sets (CIFSSs), which are parametric in nature. Through the use of the CIFSSs, parameterization techniques are applied to describe multicriteria decision-making problems. The complex Pythagorean fuzzy soft set (CPyFSS) was first introduced by Akram et al.^35^ with the application. By fusing the SS and PyFS, Peng et al.^36^ presented the Pythagorean fuzzy soft set (PyFSS) and interpreted this idea through numerous useful applications.

Later on, Cuong^37^ introduced the picture FSs (PFSs) by integrating the level of abstinence into the IFS framework. If the total of the degrees of membership, abstinence, and non-membership in a PFS falls between 0 and 1, then those values are taken from the unit interval. Son and Thong^38^ describe the use of special fuzzy calculations based on the PFS domain time arrangement for gauging and climate estimation. SFSs and the spherical fuzzy TOPSIS approach were covered by Gundogdu et al.^39^. The PFSS is a model that combines a soft set and a picture fuzzy set. Jan et al.^40^ developed the multi-valued picture fuzzy soft sets and their uses in group decision-making issues. Shanthi et al.^41^ introduced the idea of a complex picture fuzzy soft set (CPFSS). Akram et al.^42^ developed the VIKOR approach using complex spherical fuzzy-soft sets (CSFSSs) for multi-attribute decision-making (CSFSSs). The idea of hybrid decision-making frameworks under complex spherical fuzzy-soft sets was first forth by Akram et al.^43^.

The concept of CSFSS is a helpful tool for managing ambiguity and confusion in the CPFSS theory. The definition of relations for the CSFSS is still pending. This work developed the concept of complex spherical fuzzy soft relations (CSFSRs) by analysing the CP of two CSFSSs. Furthermore, different types of CSFSR have been described as well as the CSFS-reflexive relation, CSFS-symmetric relation, CSFS-transitive relation, CSFS-equivalence relation, CSFS-partial order relation, CSFS-linear order relation, CSFS-strict order relation, CSFS-converse relation, CSFS-composite relation and many more. Each CSFSR definition has been illustrated with examples. Moreover, numerous results for the type of CSFSRs have been established. The innovative idea of CSFSR is superior to pre-defined structures of SS, FSS, CFSS, IFSS, CIFSS, PyFSS and CPFSS. With more scope, the CSFSS examined levels of membership, abstinence, and non-membership. Since they use complex-valued mappings, they can also handle issues involving several variables. This article provided a method of using CSFSRs to choose the best web browser. The best web browser has been selected using the score function. This article chooses the best web browser based on the various factors that experts have suggested.

The web browser industry is dynamic, with challenges that change quickly. As a result, finding creative solutions is becoming more and more important. This urgency is a result of the ongoing efforts to improve user experience and successfully handle new problems. Given this, our work aims to break new ground in the field of web browsers by presenting the cutting edge notion of Complex Spherical Fuzzy Soft Information. This conceptual framework, which aims to reinvent how web browsers interact with and interpret data, represents a paradigm shift. Through combining concepts from complexity, spherical geometry, and fuzzy logic, our method aims to go beyond the bounds of traditional browser features. The ultimate goal is to develop a more intelligent and flexible understanding of user preferences and interactions, ushering in a new era of intelligent online experiences that are focused on the user. All things considered, this research is essentially a significant paradigm shift rather than just an evolutionary step that could change the fundamentals of web browsing.

This article’s following parts are organized as follows: All of the available fuzzy algebraic structures are contained in “Preliminaries” section. For instance, “Main results” section introduces the concept of CSFSRs and the CP of two CSFSSs. The application of a web browser is suggested in “Applications” section using the analysis of CSFSRs. The suggested structure is contrasted with the present structure in “Comparative analysis” section. The essay is concluded in “Conclusion” section.

## Preliminaries

This section defines the basic definitions of fuzzy sets, complex fuzzy sets, soft sets, fuzzy soft sets, picture fuzzy sets, score function, picture fuzzy soft sets, complex picture fuzzy soft sets, and complex spherical fuzzy soft sets for a moment.

### Definition 1

^1^A fuzzy set (FS) ${\acute{\text{\AA}}}$ over the universe $X$ is defined as

$${{{\acute{\text{\AA}}}}}=\left\{\left(f,{\psi }_{{{{\acute{\text{\AA}}}}}}\right)| f\in X\right\},$$

where ${\psi }_{{\acute{\text{\AA}}}}:X\to \left[\mathrm{0,1}\right],$ is a membership function. For each $f\in X, {\psi }_{{\acute{\text{\AA}}}}\left(f\right)$ specifies the degree to which the element $f\in {\acute{\text{\AA}}}.$

### Definition 2

^9^A complex fuzzy set (CFS) ${\acute{\text{\AA}}}$ over the universe $X$ is defined as

$${{{\acute{\text{\AA}}}}}=\left\{\left(f,{\psi }_{{{{{\acute{\text{\AA}}}}}}_{c}}(f)\right)| f\in X\right\},$$

$${\psi }_{{{{{\acute{\text{\AA}}}}}}_{c}}\left(f\right)={y}_{{\psi }_{{{{\acute{\text{\AA}}}}}}}\left(f\right){e}^{\left({x}_{{\psi }_{{{{\acute{\text{\AA}}}}}}}\left(f\right)\right){\varvec{\pi}}{{\it i}}}.$$

Since ${y}_{{\psi }_{{{{\acute{\text{\AA}}}}}}}\left(f\right),{x}_{{\psi }_{{{{\acute{\text{\AA}}}}}}}\left(f\right)\in \left[\mathrm{0,1}\right], {y}_{{\psi }_{{{{\acute{\text{\AA}}}}}}}$ and ${x}_{{\psi }_{{{{\acute{\text{\AA}}}}}}}$ are called an amplitude value and phase value of the degree of membership respectively.

### Definition 3

^14^Suppose that $X$ is a universal set, ${\text{'}}E$ is the set of parameters and ${\text{`}}P\left(X\right)$ the power set of $X$. A pair $(F, {\acute{\text{\AA}}})$ is said to be a soft set (SS) over $X,$ where ${\acute{\text{\AA}}}\subset {\text{'}}E$ and $F$ is a mapping given by $F:{\acute{\text{\AA}}}\to {{`}}P\left(X\right).$

### Example 1

Suppose that $X$ =$\left\{{w}_{1},{w}_{2},{w}_{3},{w}_{4}\right\}$ is a universal set consisting of the set off our washing-machine under consideration, and $\text{'}E=\left\{{s}_{1},{s}_{2},{s}_{3},{s}_{4}\right\}$ be the set of parameters for $X$, where each parameter ${s}_{i}, i=\mathrm{1,2},\mathrm{3,4}$ stands for beautiful, modern, expensive, and cheap respectively. Suppose a soft set $(F, {\text{'}}E)$ describes the attractiveness of the motorbikes, such that

$$F\left({s}_{1}\right)=\left\{{w}_{1},{w}_{4}\right\},$$

$$F\left({s}_{2}\right)=\left\{{w}_{1},{w}_{3},{w}_{4}\right\},$$

$$F\left({s}_{3}\right)={\{{w}_{1},w}_{3}\},$$

$$F\left({s}_{4}\right)=\left\{{w}_{2},{w}_{4}\right\}.$$

Then, the soft set $(F, {\text{'}}E)$ is a parameterized family ${\{s}_{i}, i=\mathrm{1,2},\mathrm{3,4}\}.$

### Definition 4

^19^Let $(F,{\acute{\text{\AA}}})$ and (${\c{G}}, {{B}})$ be two SSs on $X$ and ${\acute{\text{\AA}}}, {{B}}\subseteq {\text{'}}E.$ Let $(F,{\acute{\text{\AA}}})\times \left({\c{G}}, {{B}}\right)=\left(\raisebox{-5pt}{\rm H}{\hspace*{-9pt}\neg}, \raisebox{-2pt}{,}{\hspace*{-9pt}{\rm C}}\right)$ with a mapping $\raisebox{-5pt}{\rm H}{\hspace*{-9pt}\neg}:\raisebox{-2pt}{,}{\hspace*{-9pt}{\rm C}}\to {{`}}P(X)$ then the CP of SSs is denoted and defined as,

$$\raisebox{-5pt}{\rm H}{\hspace*{-9pt}\neg}\left(\raisebox{-2.5pt}{..}{\hspace*{-10pt}{\rm u}},{{v}}\right)=\left\{\left({s}_{\raisebox{-2.5pt}{..}{\hspace*{-10pt}{\rm u}}},\raisebox{-13pt}{\hbox{\^{}}}{\hspace*{-7pt}{\rm t}}_{{{v}}}\right):{s}_{\raisebox{-2.5pt}{..}{\hspace*{-10pt}{\rm u}}}\in \left(F,{\acute{\text{\AA}}}\right),\raisebox{-13pt}{\hbox{\^{}}}{\hspace*{-7pt}{\rm t}}_{{{v}}}\in \left({\c{G}}, {{B}}\right)\right\}.$$

### Definition 5

^19^Suppose that $(F,{\acute{\text{\AA}}})$ and (${\c{G}}, {{B}})$ are two SSs on $X$ and ${\acute{\text{\AA}}}, {{B}}\subseteq {\text{'}}E.$ then, a soft relation ${\check{\mathrm{R}}}$ is any subset of the CP of $(F,{\acute{\text{\AA}}})\times \left({\c{G}}, {{B}}\right).$ It is denoted and defined as,

$${\check{\mathrm{R}}} =\left\{\left({s}_{\raisebox{-2.5pt}{..}{\hspace*{-10pt}{\rm u}}},\raisebox{-13pt}{\hbox{\^{}}}{\hspace*{-7pt}{\rm t}}_{{{v}}}\right):\left({s}_{\raisebox{-2.5pt}{..}{\hspace*{-10pt}{\rm u}}},\raisebox{-13pt}{\hbox{\^{}}}{\hspace*{-7pt}{\rm t}}_{{{v}}}\right)\in \left(F,{\acute{\text{\AA}}}\right)\times \left({\c{G}}, {{B}}\right)\right\}.$$

### Definition 6

^21^Suppose that $X$ is a universal set, $\text{'}E$ is a set of parameters and ${{`}}P\left(X\right)$ is the set of all fuzzy subsets of $X$. A pair $(F, {\acute{\text{\AA}}})$ is said to be a fuzzy soft set (FSS) over $X$, where ${\acute{\text{\AA}}}\subset {\text{'}}E$ and $F$ is a mapping given by $F:{\acute{\text{\AA}}}\to {{`}}P\left(X\right)$.

### Example 2

Suppose that $X$ is the set of scissor companies and $\text{'}E$ is the set of parameters. Suppose that a fuzzy soft set $\left(F, {\text{'}}E\right)$ depicts the characteristics of the scissor in relation to some parameter and each membership degree assigned by experts. $X=\left\{{{ot{\acute{\mathrm{S}}}}}_{1},{{ot{\acute{\mathrm{S}}}}}_{2},{{ot{\acute{\mathrm{S}}}}}_{3},{{ot{\acute{\mathrm{S}}}}}_{4}\right\}$ i.e., ${{ot{\acute{\mathrm{S}}}}}_{1}=$ Smith Chu, ${{ot{\acute{\mathrm{S}}}}}_{2}$ = Brainbow, ${{ot{\acute{\mathrm{S}}}}}_{3}=$ Damascus, and ${{ot{\acute{\mathrm{S}}}}}_{4}$ = Dawlance. $\text{'}E =\left\{{s}_{1},{s}_{2},{s}_{3}\right\}$ i.e., ${s}_{1}=$ Design, ${s}_{2}=$ beautifull, and ${s}_{3}=$ Digital.

$$F\left({s}_{1}\right)=\left\{{{ot{\acute{\mathrm{S}}}}}_{1}=0.4,{{ot{\acute{\mathrm{S}}}}}_{2}=0.6,{{ot{\acute{\mathrm{S}}}}}_{3}=0.7,{{ot{\acute{\mathrm{S}}}}}_{4}=0.2\right\},$$

$$F\left({s}_{2}\right)=\left\{{{ot{\acute{\mathrm{S}}}}}_{1}=0.5,{{ot{\acute{\mathrm{S}}}}}_{2}=0.7,{{ot{\acute{\mathrm{S}}}}}_{3}=0.7,{{ot{\acute{\mathrm{S}}}}}_{4}=0.6\right\},$$

$$F\left({s}_{3}\right)=\left\{{{ot{\acute{\mathrm{S}}}}}_{1}=0.3,{{ot{\acute{\mathrm{S}}}}}_{2}=0.6,{{ot{\acute{\mathrm{S}}}}}_{3}=0.4,{{ot{\acute{\mathrm{S}}}}}_{4}=0.7\right\}.$$

The $\left(F,\text{'}E\right)$ is a parameterized family ${\{F(s}_{i}), i=\mathrm{1,2},3\}.$

### Definition 7

^37^A picture fuzzy set (PFS) ${\acute{\text{\AA}}}$ over the universe $X$ is defined as

$${\acute{\text{\AA}}}=\left\{\left(f,{\psi }_{{\acute{\text{\AA}}}},{\mu }_{{\acute{\text{\AA}}}},{\eta }_{{\acute{\text{\AA}}}}\right)| f\in X\right\},$$

where ${\psi }_{{\acute{\text{\AA}}}}\left(f\right)\in \left[\mathrm{0,1}\right]$ is called the degree of membership of $f$ in ${\acute{\text{\AA}}},{\mu }_{{\acute{\text{\AA}}}}\left(f\right)\in \left[\mathrm{0,1}\right]$ is called the degree of abstinence of $f$ in ${\acute{\text{\AA}}}$ and ${\eta }_{{\acute{\text{\AA}}}}\left(f\right)\in \left[\mathrm{0,1}\right]$ is called the degree of non-membership of $f$ in ${\acute{\text{\AA}}}$, which is satisfying the following condition $0\le ({\psi }_{{\acute{\text{\AA}}}}\left(f\right)+{\mu }_{{\acute{\text{\AA}}}}\left(f\right)+{\eta }_{{\acute{\text{\AA}}}}\left(f\right))\le 1, \forall f\in X.$ For PFS $\left({\psi }_{{\acute{\text{\AA}}}}\left(f\right),{\mu }_{{\acute{\text{\AA}}}}\left(f\right),{\eta }_{{\acute{\text{\AA}}}}\left(f\right)\right)$ are called picture fuzzy value (PFV) or picture fuzzy number (PFN) and each PFV can be denoted by $q=\left({\psi }_{q},{\mu }_{q},{\eta }_{q}\right)$, where ${\psi }_{q},{\mu }_{q}\, and \, {\eta }_{q}\in \left[\mathrm{0,1}\right],$ which satisfy the following condition $0\le \left({\psi }_{{\acute{\text{\AA}}}}\left(f\right)+{\mu }_{{\acute{\text{\AA}}}}\left(f\right)+{\eta }_{{\acute{\text{\AA}}}}\left(f\right)\right)\le 1$.

### Definition 8

^38^Suppose that $q=\left({\psi }_{q},{\mu }_{q},{\eta }_{q}\right)$ is a PFV, then their score function $\omega$ is defined as

$$\omega \left(q\right)={\psi }_{q}-{\eta }_{q}, \omega \left(q\right)\in \left[-\mathrm{1,1}\right].$$

### Definition 9

^40^Suppose that $X$ is a universal set, $\text{'}E$ is a set of parameters and ${{`}}PF\left(X\right)$ is the set of all picture fuzzy subsets of $X$. A pair $(F, {\acute{\text{\AA}}})$ is said to be a picture fuzzy soft set (PFSS) over $X$, where ${\acute{\text{\AA}}}\subset {\text{'}}E$ and $F$ is a mapping given by $F:{\acute{\text{\AA}}}\to {{`}}PF\left(X\right)$.

### Example 3

From Example 2, assume a PFSS $\left(F, {\text{'}}E\right)$ describing the characteristic of the scissor with respect to some parameter, and each membership, abstinence, and non-membership degree given by experts.

$$F\left({s}_{1}\right)=\left\{{ {ot{\acute{\mathrm{S}}}}}_{1}=\left(\mathrm{0.3,0.3,0.4}\right),{ {ot{\acute{\mathrm{S}}}}}_{2}=\left(\mathrm{0.5,0.1,0.3}\right),{ {ot{\acute{\mathrm{S}}}}}_{3}=(\mathrm{0.3,0.2,0.2}){, {ot{\acute{\mathrm{S}}}}}_{4}=(\mathrm{0.2,0.6,0.1})\right\},$$

$$F\left({s}_{2}\right)=\left\{{ {ot{\acute{\mathrm{S}}}}}_{1}=\left(\mathrm{0.4,0.3,0.1}\right),{ {ot{\acute{\mathrm{S}}}}}_{2}=\left(\mathrm{0.4,0.3,0.1}\right),{ {ot{\acute{\mathrm{S}}}}}_{3}=\left(\mathrm{0.3,0.1,0.2}\right),{ {ot{\acute{\mathrm{S}}}}}_{4}=(\mathrm{0.5,0.2,0.2})\right\},$$

$$F\left({s}_{3}\right)=\left\{{ {ot{\acute{\mathrm{S}}}}}_{1}=\left(\mathrm{0.4,0.1,0.2}\right),{ {ot{\acute{\mathrm{S}}}}}_{2}=\left(\mathrm{0.2,0.4,0.3}\right),{ {ot{\acute{\mathrm{S}}}}}_{3}=\left(\mathrm{0.2,0.2}.0.4\right),{ {ot{\acute{\mathrm{S}}}}}_{4}=(\mathrm{0.3,0.2,0.4})\right\}.$$

Then, the PFSS $\left(F, {\text{'}}E\right)$ is a parameterized family $\{F\left({s}_{i}\right),i=\mathrm{1,2},3\}$.

### Definition 10

^40^Suppose that $X$ is a universal set, $\text{'}E$ is a set of parameters and $C{\text{`}}PF\left(X\right)$ is the set of all complex picture fuzzy subsets of $X$. A pair $(F, {\acute{\text{\AA}}})$ is said to be a complex picture fuzzy soft set (CPFSS) over $X$, where ${\acute{\text{\AA}}}\subset {\text{'}}E$ and $F$ is a mapping given by $F:{\acute{\text{\AA}}}\to {{`}}PF\left(X\right)$ defined by the set of ordered pair,

$$F=\left\{\left(f,{\psi }_{{{\acute{\text{\AA}}}}_{c}}\left(f\right),{\mu }_{{{\acute{\text{\AA}}}}_{c}}\left(f\right),{\eta }_{{{\acute{\text{\AA}}}}_{c}}\left(f\right)\right)| f\in {\text{'}}E , {\psi }_{{{\acute{\text{\AA}}}}_{c}}\left(f\right),{\mu }_{{{\acute{\text{\AA}}}}_{c}}\left(f\right),{\eta }_{{{\acute{\text{\AA}}}}_{c}}\left(f\right)\in C{\text{`}}PF\left(X\right)\right\},$$

$$F=\left\{\left(f,{y}_{{\psi }_{{\acute{\text{\AA}}}}}\left(f\right){e}^{{x}_{{\psi }_{{\acute{\text{\AA}}}}}\left(f\right){\varvec{\pi}}{{\it i}}},{y}_{{\mu }_{{\acute{\text{\AA}}}}}\left(f\right){e}^{{x}_{{\mu }_{{\acute{\text{\AA}}}}}\left(f\right){\varvec{\pi}}{{\it i}}},{y}_{{\eta }_{{\acute{\text{\AA}}}}}\left(f\right){e}^{{x}_{{\eta }_{{\acute{\text{\AA}}}}}\left(f\right){\varvec{\pi}}{{\it i}}}\right):f\in {\text{'}}E\right\},$$

where ${y}_{{\psi }_{{\acute{\text{\AA}}}}}, {y}_{{\mu }_{{\acute{\text{\AA}}}}}\,and\,{y}_{{\eta }_{{\acute{\text{\AA}}}}}$ are called an amplitude term of membership, abstinence and non-membership degree respectively, and ${x}_{{\psi }_{{\acute{\text{\AA}}}}},{x}_{{\mu }_{{\acute{\text{\AA}}}}},$ and ${x}_{{\eta }_{{\acute{\text{\AA}}}}}$ are called the phase terms of membership, abstinence and non-membership degree, respectively. It satisfied the following conditions

$$0\le \left({y}_{{\psi }_{{\acute{\text{\AA}}}}}\right)+\left({y}_{{\mu }_{{\acute{\text{\AA}}}}}\right)+\left({y}_{{\eta }_{{\acute{\text{\AA}}}}}\right)\le 1 \, \mathrm{and}\,0\le \left({x}_{{\psi }_{{\acute{\text{\AA}}}}}\right)+\left({x}_{{\mu }_{{\acute{\text{\AA}}}}}\right)+\left({x}_{{\eta }_{{\acute{\text{\AA}}}}}\right)\le 1$$

### Definition 11

^42^Suppose that $X$ is a universal set, $\text{'}E$ is a set of parameters and $CSF\left(X\right)$ is the set of all complex spherical fuzzy subsets of $X$. A pair $(F, {\acute{\text{\AA}}})$ is said to be a complex spherical fuzzy soft set (CSFSS) over $X$, where ${\acute{\text{\AA}}}\subset {\text{'}}E$ and $F$ is a mapping given by $F:{\acute{\text{\AA}}}\to {{`}}PF\left(X\right)$ defined by the set of ordered pairs,

$$F=\left\{\left(f,{\psi }_{{{\acute{\text{\AA}}}}_{c}}\left(f\right),{\mu }_{{{\acute{\text{\AA}}}}_{c}}\left(f\right),{\eta }_{{{\acute{\text{\AA}}}}_{c}}\left(f\right)\right)| f\in {\text{'}}E , {\psi }_{{{\acute{\text{\AA}}}}_{c}}\left(f\right),{\mu }_{{{\acute{\text{\AA}}}}_{c}}\left(f\right),{\eta }_{{{\acute{\text{\AA}}}}_{c}}\left(f\right)\in C{\text{`}}PF\left(X\right)\right\},$$

$$F=\left\{\left(f,{y}_{{\psi }_{{\acute{\text{\AA}}}}}\left(f\right){e}^{{x}_{{\psi }_{{\acute{\text{\AA}}}}}\left(f\right){\varvec{\pi}}{{\it i}}},{y}_{{\mu }_{{\acute{\text{\AA}}}}}\left(f\right){e}^{{x}_{{\mu }_{{\acute{\text{\AA}}}}}\left(f\right){\varvec{\pi}}{{\it i}}},{y}_{{\eta }_{{\acute{\text{\AA}}}}}\left(f\right){e}^{{x}_{{\eta }_{{\acute{\text{\AA}}}}}\left(f\right){\varvec{\pi}}{{\it i}}}\right):f\in {\text{'}}E\right\},$$

where ${y}_{{\psi }_{{\acute{\text{\AA}}}}}, {y}_{{\mu }_{{\acute{\text{\AA}}}}}\,and\,{y}_{{\eta }_{{\acute{\text{\AA}}}}}$ are called an amplitude term of membership, abstinence and non-membership degree respectively, and ${x}_{{\psi }_{{\acute{\text{\AA}}}}},{x}_{{\mu }_{{\acute{\text{\AA}}}}},$ and ${x}_{{\eta }_{{\acute{\text{\AA}}}}}$ are called the phase terms of membership, abstinence and non-membership degree, respectively. It satisfied the following conditions

$$0\le {\left({y}_{{\psi }_{{\acute{\text{\AA}}}}}\right)}^{2}+{\left({y}_{{\mu }_{{\acute{\text{\AA}}}}}\right)}^{2}+{\left({y}_{{\eta }_{{\acute{\text{\AA}}}}}\right)}^{2}\le 1 \, {\text{and}} \, 0\le {\left({x}_{{\psi }_{{\acute{\text{\AA}}}}}\right)}^{2}+{\left({x}_{{\mu }_{{\acute{\text{\AA}}}}}\right)}^{2}+{\left({x}_{{\eta }_{{\acute{\text{\AA}}}}}\right)}^{2}\le 1.$$

## Main results

In this section, explain the idea of the CP of two CSFSSs, their relations, and different types of relations.

### Definition 12

Suppose that ($F,{\acute{\text{\AA}}})$ and ($\c{G}, {B})$ are two complex spherical fuzzy soft sets (CSFSSs) on $X$ and ${\acute{\text{\AA}}}, {B}\subseteq {\text{'}}E.$ Let ($F,{\acute{\text{\AA}}})\times \left(\c{G}, {B}\right)=\left({\raisebox{-5pt}{\rm H}{\hspace*{-9pt}\neg}},\raisebox{-2pt}{,}{\hspace*{-9pt}{\rm C}}\right)$ with a mapping ${\raisebox{-5pt}{\rm H}{\hspace*{-9pt}\neg}}:\raisebox{-2pt}{,}{\hspace*{-9pt}{\rm C}}\to \raisebox{-2pt}{,}{\hspace*{-9pt}{\rm C}}{\text{`}}P F(X)$ and ${\raisebox{-5pt}{\rm H}{\hspace*{-9pt}\neg}}\left({\raisebox{-2.5pt}{..}{\hspace*{-10pt}{\rm u}}},{{v}}\right)=F\left({\raisebox{-2.5pt}{..}{\hspace*{-10pt}{\rm u}}}\right)\times \breve{G} \left({{v}}\right),$ then the CP of CSFSSs is denoted by,

$${\acute{\text{\AA}}}=\left\{\left(s,{{\text{y}}}_{{\psi }_{{\acute{\text{\AA}}}}}^{{\acute{\text{\AA}}}}\left(s\right){e}^{{x}_{{\psi }_{{\acute{\text{\AA}}}}}^{{\acute{\text{\AA}}}}\left(s\right){\varvec{\pi}}{{\it i}}},{{\text{y}}}_{{\mu }_{{\acute{\text{\AA}}}}}^{{\acute{\text{\AA}}}}\left(s\right){e}^{{x}_{{\mu }_{{\acute{\text{\AA}}}}}^{{\acute{\text{\AA}}}}\left(s\right){\varvec{\pi}}{{\it i}}},{{\text{y}}}_{{\eta }_{{\acute{\text{\AA}}}}}^{{\acute{\text{\AA}}}}\left(s\right){e}^{{x}_{{\eta }_{{\acute{\text{\AA}}}}}^{{\acute{\text{\AA}}}}\left(s\right){\varvec{\pi}}{{\it i}}}\right):s\in {\text{'}}E\right\}\,and,$$

$${{B}}=\left\{\left(f,{{\text{y}}}_{{\psi }_{{\acute{\text{\AA}}}}}^{{B}}\left(f\right){e}^{{x}_{{\psi }_{{\acute{\text{\AA}}}}}^{{B}}\left(f\right){\varvec{\pi}}{{\it i}}},{{\text{y}}}_{{\mu }_{{\acute{\text{\AA}}}}}^{{B}}\left(f\right){e}^{{x}_{{\mu }_{{\acute{\text{\AA}}}}}^{{B}}\left(f\right){\varvec{\pi}}{{\it i}}},{{\text{y}}}_{{\eta }_{{\acute{\text{\AA}}}}}^{{B}}\left(f\right){e}^{{x}_{{\eta }_{{\acute{\text{\AA}}}}}^{{B}}\left(f\right){\varvec{\pi}}{{\it i}}}\right):f\in {\text{'}}E\right\},$$

and is defined as,

$$\left({\raisebox{-5pt}{\rm H}{\hspace*{-9pt}\neg}},\raisebox{-2pt}{,}{\hspace*{-9pt}{\rm C}}\right)= {\acute{\text{\AA}}}\times {B}=\left\{\left(\begin{array}{c}\left(s,f\right),{{\text{y}}}_{{\psi }_{{\acute{\text{\AA}}}}}^{{\acute{\text{\AA}}}\times {B}}\left(s,f\right){e}^{{x}_{{\psi }_{{\acute{\text{\AA}}}}}^{{\acute{\text{\AA}}}\times {B}}\left(s,f\right){\varvec{\pi}}{{\it i}}},{{\text{y}}}_{{\mu }_{{\acute{\text{\AA}}}}}^{{\acute{\text{\AA}}}\times {B}}\left(s,f\right){e}^{{x}_{{\mu }_{{\acute{\text{\AA}}}}}^{{\acute{\text{\AA}}}\times {B}}\left(s,f\right){\varvec{\pi}}{{\it i}}},\\ {{\text{y}}}_{{\eta }_{{\acute{\text{\AA}}}}}^{{\acute{\text{\AA}}}\times {B}}\left(s,f\right){e}^{{x}_{{\eta }_{{\acute{\text{\AA}}}}}^{{\acute{\text{\AA}}}\times {B}}\left(s,f\right){\varvec{\pi}}{{\it i}}}\end{array}\right):s\in {\acute{\text{\AA}}},f\in {B}\right\},$$

where

$$\left\{\begin{array}{c}{{\text{y}}}_{{\psi }_{{\acute{\text{\AA}}}}}^{{\acute{\text{\AA}}}\times {B}}\left(s,f\right)=min\left\{{{\text{y}}}_{{\psi }_{{\acute{\text{\AA}}}}}^{{\acute{\text{\AA}}}}\left(s\right),{{\text{y}}}_{{\psi }_{{\acute{\text{\AA}}}}}^{{B}}\left(f\right)\right\}, {{\text{y}}}_{{\mu }_{{\acute{\text{\AA}}}}}^{{\acute{\text{\AA}}}\times {B}}\left(s,f\right)=min\left\{{{\text{y}}}_{{\mu }_{{\acute{\text{\AA}}}}}^{{\acute{\text{\AA}}}}\left(s\right),{{\text{y}}}_{{\mu }_{{\acute{\text{\AA}}}}}^{{B}}\left(f\right)\right\},\\ and\,{{\text{y}}}_{{\eta }_{{\acute{\text{\AA}}}}}^{{\acute{\text{\AA}}}\times {B}}\left(s,f\right)=max\left\{{{\text{y}}}_{{\eta }_{{\acute{\text{\AA}}}}}^{{\acute{\text{\AA}}}}\left(s\right),{{\text{y}}}_{{\eta }_{{\acute{\text{\AA}}}}}^{{B}}\left(f\right)\right\},\end{array}\right\},$$

$$\left\{\begin{array}{c}{x}_{{\psi }_{{\acute{\text{\AA}}}}}^{{\acute{\text{\AA}}}\times {B}}\left(s,f\right)=min\left\{{x}_{{\psi }_{{\acute{\text{\AA}}}}}^{{\acute{\text{\AA}}}}\left(s\right),{x}_{{\psi }_{{\acute{\text{\AA}}}}}^{{B}}\left(f\right)\right\},{x}_{{\mu }_{{\acute{\text{\AA}}}}}^{{\acute{\text{\AA}}}\times {B}}\left(s,f\right)=min\left\{{x}_{{\mu }_{{\acute{\text{\AA}}}}}^{{\acute{\text{\AA}}}}\left(s\right),{x}_{{\mu }_{{\acute{\text{\AA}}}}}^{{B}}\left(f\right)\right\},\\ and\,{x}_{{\eta }_{{\acute{\text{\AA}}}}}^{{\acute{\text{\AA}}}\times {B}}\left(s,f\right)=max\left\{{x}_{{\eta }_{{\acute{\text{\AA}}}}}^{{\acute{\text{\AA}}}}\left(s\right),{x}_{{\eta }_{{\acute{\text{\AA}}}}}^{{B}}\left(f\right)\right\}\end{array}\right\}.$$

### Example 4

Suppose the universal set $X=\left\{{c}_{1},{c}_{2},{c}_{3}\right\}$ consists of three types of mobile phone charger companies, i.e., ${c}_{1}=$ Sumsung, ${c}_{2}=$ Oppo, and ${c}_{3}=$ Apple and there are three parameters $\text{'}E=\left\{{s}_{1},{s}_{2},{s}_{3}\right\}$, i.e., ${s}_{1}=$ medium, ${s}_{2}=$ Fast and ${s}_{3}=$ slow. Let ($F,{\acute{\text{\AA}}})$ and ($\c{G}, {B})$ be two CSFSSs by two experts ${\acute{\text{\AA}}}$ and ${B}$ respectively.

Let their corresponding membership, abstinence, and non-membership as follows; for $n=2$

$$\left(F,{\acute{\text{\AA}}}\right)=\left(\begin{array}{c}\left({s}_{1},\left(\begin{array}{c}0.44{e}^{(0.29)\pi {{\it i}}},\\ 0.40{e}^{(0.30)\pi {{\it i}}},\\ 0.22{e}^{\left(0.38\right)\pi {{\it i}}}\end{array}\right),\left(\begin{array}{c}0.48{e}^{\left(0.47\right)\pi {{\it i}}},\\ 0.41{e}^{\left(0.40\right)\pi {{\it i}}},\\ 0.39{e}^{\left(0.35\right)\pi {{\it i}}}\end{array}\right),\left(\begin{array}{c}0.53{e}^{\left(0.50\right)\pi {{\it i}}},\\ 0.57{e}^{\left(0.33\right)\pi {{\it i}}},\\ 0.31{e}^{\left(0.48\right)\pi {{\it i}}}\end{array}\right),\left(\begin{array}{c}0.51{e}^{\left(0.30\right)\pi {{\it i}}},\\ 0.49{e}^{\left(0.29\right)\pi {{\it i}}},\\ 0.23{e}^{\left(0.40\right)\pi {{\it i}}}\end{array}\right)\right),\\ \left({s}_{2},\left(\begin{array}{c}0.48{e}^{\left(0.31\right)\pi {{\it i}}},\\ 0.49{e}^{\left(0.23\right)\pi {{\it i}}},\\ 0.27{e}^{\left(0.37\right)\pi {{\it i}}}\end{array}\right),\left(\begin{array}{c}0.49{e}^{\left(0.23\right)\pi {{\it i}}},\\ 0.57{e}^{\left(0.39\right)\pi {{\it i}}},\\ 0.38{e}^{\left(0.36\right)\pi {{\it i}}}\end{array}\right),\left(\begin{array}{c}0.46{e}^{\left(0.34\right)\pi {{\it i}}},\\ 0.35{e}^{\left(0.48\right)\pi {{\it i}}},\\ 0.34{e}^{\left(0.39\right)\pi {{\it i}}}\end{array}\right),\left(\begin{array}{c}0.49{e}^{\left(0.36\right)\pi {{\it i}}},\\ 0.43{e}^{\left(0.47\right)\pi {{\it i}}},\\ 0.31{e}^{\left(0.48\right)\pi {{\it i}}}\end{array}\right)\right),\\ \left({s}_{3},\left(\begin{array}{c}0.55{e}^{\left(0.34\right)\pi {{\it i}}},\\ 0.45{e}^{\left(0.39\right)\pi {{\it i}}},\\ 0.35{e}^{\left(0.40\right)\pi {{\it i}}}\end{array}\right),\left(\begin{array}{c}0.58{e}^{\left(0.48\right)\pi {{\it i}}},\\ 0.4{9e}^{\left(0.33\right)\pi {{\it i}}},\\ 0.41{e}^{\left(0.40\right)\pi {{\it i}}}\end{array}\right),\left(\begin{array}{c}0.53{e}^{\left(0.49\right)\pi {{\it i}}},\\ 0.42{e}^{\left(0.48\right)\pi {{\it i}}},\\ 0.39{e}^{\left(0.40\right)\pi {{\it i}}}\end{array}\right),\left(\begin{array}{c}0.54{e}^{\left(0.47\right)\pi {{\it i}}},\\ 0.53{e}^{\left(0.41\right)\pi {{\it i}}},\\ 0.40{e}^{\left(0.33\right)\pi {{\it i}}}\end{array}\right)\right)\end{array}\right),$$

and,

$$(\breve{G} ,{B})=\left(\begin{array}{c}\left({s}_{1},\left(\begin{array}{c}0.49{e}^{(0.45)\pi {{\it i}}},\\ 0.35{e}^{(0.49)\pi {{\it i}}},\\ 0.34{e}^{\left(0.40\right)\pi {{\it i}}}\end{array}\right),\left(\begin{array}{c}0.58{e}^{\left(0.41\right)\pi {{\it i}}},\\ 0.47{e}^{\left(0.40\right)\pi {{\it i}}},\\ 0.39{e}^{\left(0.45\right)\pi {{\it i}}}\end{array}\right),\left(\begin{array}{c}0.52{e}^{\left(0.41\right)\pi {{\it i}}},\\ 0.53{e}^{\left(0.40\right)\pi {{\it i}}},\\ 0.51{e}^{\left(0.30\right)\pi {{\it i}}}\end{array}\right),\left(\begin{array}{c}0.61{e}^{\left(0.40\right)\pi {{\it i}}},\\ 0.35{e}^{\left(0.35\right)\pi {{\it i}}},\\ 0.43{e}^{\left(0.49\right)\pi {{\it i}}}\end{array}\right)\right),\\ \left({s}_{2},\left(\begin{array}{c}0.55{e}^{\left(0.48\right)\pi {{\it i}}},\\ 0.49{e}^{\left(0.42\right)\pi {{\it i}}},\\ 0.37{e}^{\left(0.40\right)\pi {{\it i}}}\end{array}\right),\left(\begin{array}{c}0.51{e}^{\left(0.40\right)\pi {{\it i}}},\\ 0.49{e}^{\left(0.38\right)\pi {{\it i}}},\\ 0.41{e}^{\left(0.40\right)\pi {{\it i}}}\end{array}\right),\left(\begin{array}{c}0.54{e}^{\left(0.41\right)\pi {{\it i}}},\\ 0.52{e}^{\left(0.39\right)\pi {{\it i}}},\\ 0.38{e}^{\left(0.39\right)\pi {{\it i}}}\end{array}\right),\left(\begin{array}{c}0.58{e}^{\left(0.41\right)\pi {{\it i}}},\\ 0.35{e}^{\left(0.52\right)\pi {{\it i}}},\\ 0.45{e}^{\left(0.51\right)\pi {{\it i}}}\end{array}\right)\right),\\ \left({s}_{3},\left(\begin{array}{c}0.59{e}^{\left(0.43\right)\pi {{\it i}}},\\ 0.46{e}^{\left(0.40\right)\pi {{\it i}}},\\ 0.40{e}^{\left(0.39\right)\pi {{\it i}}}\end{array}\right),\left(\begin{array}{c}0.52{e}^{\left(0.39\right)\pi {{\it i}}},\\ 0.54{e}^{\left(0.43\right)\pi {{\it i}}},\\ 0.40{e}^{\left(0.45\right)\pi {{\it i}}}\end{array}\right),\left(\begin{array}{c}0.60{e}^{\left(0.45\right)\pi {{\it i}}},\\ 0.41{e}^{\left(0.46\right)\pi {{\it i}}},\\ 0.38{e}^{\left(0.40\right)\pi {{\it i}}}\end{array}\right),\left(\begin{array}{c}0.64{e}^{\left(0.46\right)\pi {{\it i}}},\\ 0.51{e}^{\left(0.41\right)\pi {{\it i}}},\\ 0.43{e}^{\left(0.46\right)\pi {{\it i}}}\end{array}\right)\right),\end{array}\right).$$

In the observations that are being provided, the first three values for each parameter are crucial in defining the levels of membership, abstinence, and non-membership that are assigned to each company that is being examined. The complex and dynamic relationships between the companies and the parameters are captured by these numerical representations, which demonstrate the intricate nature of their affiliations. In this set of observations, the fourth value takes on a special significance as the overall or general value that is widely recognized as the degree of belongingness. This all-inclusive value functions as a comprehensive indicator, offering a consolidated viewpoint on each company’s degree of association or relevance to the specified parameters. Through the integration of these varied numerical values, this methodology not only provides a detailed comprehension of the positions of individual companies, but also enables a more comprehensive evaluation of their joint participation, thereby augmenting and enlightening the intricate relationship between companies and the parameters under consideration. Each row represents the parametric observations. Then their CP of $\left(F, {\acute{\text{\AA}}}\right)$ and ($\c{G}, {B})$ is denoted by $\left({\raisebox{-5pt}{\rm H}{\hspace*{-9pt}\neg}}, \raisebox{-2pt}{,}{\hspace*{-9pt}{\rm C}}\right)$ and defined as Table 1.

**Table 1:** CP of $(F, {\acute{\text{\AA}}})$ and ($\c{G}, {B})$.

Ordered pair	${c}_{1}$	${c}_{2}$	${c}_{3}$	$\lambda$
$\left({s}_{1},{s}_{1}\right)$	$$\left(\begin{array}{c}0.44{e}^{(0.29)\pi {{\it i}}},\\ 0.35{e}^{(0.30)\pi {{\it i}}},\\ 0.34{e}^{\left(0.40\right)\pi {{\it i}}}\end{array}\right)$$	$$\left(\begin{array}{c}0.48{e}^{\left(0.41\right)\pi {{\it i}}},\\ 0.41{e}^{\left(0.40\right)\pi {{\it i}}},\\ 0.39{e}^{\left(0.45\right)\pi {{\it i}}}\end{array}\right)$$	$$\left(\begin{array}{c}0.52{e}^{\left(0.41\right)\pi {{\it i}}},\\ 0.53{e}^{\left(0.33\right)\pi {{\it i}}},\\ 0.51{e}^{\left(0.48\right)\pi {{\it i}}}\end{array}\right)$$	$$\left(\begin{array}{c}0.51{e}^{\left(0.30\right)\pi {{\it i}}},\\ 0.35{e}^{\left(0.29\right)\pi {{\it i}}},\\ 0.43{e}^{\left(0.49\right)\pi {{\it i}}}\end{array}\right)$$
$\left({s}_{1},{s}_{2}\right)$	$$\left(\begin{array}{c}0.44{e}^{(0.29)\pi {{\it i}}},\\ 0.40{e}^{(0.30)\pi {{\it i}}},\\ 0.37{e}^{\left(0.40\right)\pi {{\it i}}}\end{array}\right)$$	$$\left(\begin{array}{c}0.48{e}^{\left(0.40\right)\pi {{\it i}}},\\ 0.41{e}^{\left(0.38\right)\pi {{\it i}}},\\ 0.41{e}^{\left(0.40\right)\pi {{\it i}}}\end{array}\right)$$	$$\left(\begin{array}{c}0.53{e}^{\left(0.41\right)\pi {{\it i}}},\\ 0.52{e}^{\left(0.33\right)\pi {{\it i}}},\\ 0.38{e}^{\left(0.48\right)\pi {{\it i}}}\end{array}\right)$$	$$\left(\begin{array}{c}0.51{e}^{\left(0.30\right)\pi {{\it i}}},\\ 0.35{e}^{\left(0.29\right)\pi {{\it i}}},\\ 0.45{e}^{\left(0.51\right)\pi {{\it i}}}\end{array}\right)$$
$\left({s}_{1},{s}_{3}\right)$	$$\left(\begin{array}{c}0.44{e}^{(0.29)\pi {{\it i}}},\\ 0.40{e}^{(0.30)\pi {{\it i}}},\\ 0.40{e}^{\left(0.39\right)\pi {{\it i}}}\end{array}\right)$$	$$\left(\begin{array}{c}0.48{e}^{\left(0.39\right)\pi {{\it i}}},\\ 0.41{e}^{\left(0.40\right)\pi {{\it i}}},\\ 0.40{e}^{\left(0.45\right)\pi {{\it i}}}\end{array}\right)$$	$$\left(\begin{array}{c}0.53{e}^{\left(0.45\right)\pi {{\it i}}},\\ 0.41{e}^{\left(0.33\right)\pi {{\it i}}},\\ 0.38{e}^{\left(0.48\right)\pi {{\it i}}}\end{array}\right)$$	$$\left(\begin{array}{c}0.51{e}^{\left(0.30\right)\pi {{\it i}}},\\ 0.49{e}^{\left(0.29\right)\pi {{\it i}}},\\ 0.43{e}^{\left(0.46\right)\pi {{\it i}}}\end{array}\right)$$
$\left({s}_{2},{s}_{1}\right)$	$$\left(\begin{array}{c}0.48{e}^{(0.31)\pi {{\it i}}},\\ 0.35{e}^{(0.23)\pi {{\it i}}},\\ 0.34{e}^{\left(0.40\right)\pi {{\it i}}}\end{array}\right)$$	$$\left(\begin{array}{c}0.49{e}^{\left(0.23\right)\pi {{\it i}}},\\ 0.47{e}^{\left(0.39\right)\pi {{\it i}}},\\ 0.39{e}^{\left(0.45\right)\pi {{\it i}}}\end{array}\right)$$	$$\left(\begin{array}{c}0.46{e}^{\left(0.34\right)\pi {{\it i}}},\\ 0.35{e}^{\left(0.40\right)\pi {{\it i}}},\\ 0.51{e}^{\left(0.39\right)\pi {{\it i}}}\end{array}\right)$$	$$\left(\begin{array}{c}0.49{e}^{\left(0.36\right)\pi {{\it i}}},\\ 0.35{e}^{\left(0.35\right)\pi {{\it i}}},\\ 0.43{e}^{\left(0.49\right)\pi {{\it i}}}\end{array}\right)$$
$\left({s}_{2,}{s}_{2}\right)$	$$\left(\begin{array}{c}0.48{e}^{(0.31)\pi {{\it i}}},\\ 0.49{e}^{(0.23)\pi {{\it i}}},\\ 0.37{e}^{\left(0.40\right)\pi {{\it i}}}\end{array}\right)$$	$$\left(\begin{array}{c}0.49{e}^{\left(0.23\right)\pi {{\it i}}},\\ 0.49{e}^{\left(0.38\right)\pi {{\it i}}},\\ 0.41{e}^{\left(0.40\right)\pi {{\it i}}}\end{array}\right)$$	$$\left(\begin{array}{c}0.46{e}^{\left(0.34\right)\pi {{\it i}}},\\ 0.35{e}^{\left(0.39\right)\pi {{\it i}}},\\ 0.38{e}^{\left(0.39\right)\pi {{\it i}}}\end{array}\right)$$	$$\left(\begin{array}{c}0.49{e}^{\left(0.36\right)\pi {{\it i}}},\\ 0.35{e}^{\left(0.47\right)\pi {{\it i}}},\\ 0.45{e}^{\left(0.51\right)\pi {{\it i}}}\end{array}\right)$$
$\left({s}_{2},{s}_{3}\right)$	$$\left(\begin{array}{c}0.48{e}^{(0.31)\pi {{\it i}}},\\ 0.46{e}^{(0.23)\pi {{\it i}}},\\ 0.40{e}^{\left(0.39\right)\pi {{\it i}}}\end{array}\right)$$	$$\left(\begin{array}{c}0.49{e}^{\left(0.23\right)\pi {{\it i}}},\\ 0.54{e}^{\left(0.39\right)\pi {{\it i}}},\\ 0.40{e}^{\left(0.45\right)\pi {{\it i}}}\end{array}\right)$$	$$\left(\begin{array}{c}0.46{e}^{\left(0.34\right)\pi {{\it i}}},\\ 0.35{e}^{\left(0.46\right)\pi {{\it i}}},\\ 0.38{e}^{\left(0.40\right)\pi {{\it i}}}\end{array}\right)$$	$$\left(\begin{array}{c}0.49{e}^{\left(0.36\right)\pi {{\it i}}},\\ 0.43{e}^{\left(0.41\right)\pi {{\it i}}},\\ 0.43{e}^{\left(0.48\right)\pi {{\it i}}}\end{array}\right)$$
$\left({s}_{3},{s}_{1}\right)$	$$\left(\begin{array}{c}0.49{e}^{(0.34)\pi {{\it i}}},\\ 0.35{e}^{(0.39)\pi {{\it i}}},\\ 0.35{e}^{\left(0.40\right)\pi {{\it i}}}\end{array}\right)$$	$$\left(\begin{array}{c}0.58{e}^{\left(0.41\right)\pi {{\it i}}},\\ 0.47{e}^{\left(0.33\right)\pi {{\it i}}},\\ 0.41{e}^{\left(0.45\right)\pi {{\it i}}}\end{array}\right)$$	$$\left(\begin{array}{c}0.52{e}^{\left(0.41\right)\pi {{\it i}}},\\ 0.42{e}^{\left(0.40\right)\pi {{\it i}}},\\ 0.51{e}^{\left(0.40\right)\pi {{\it i}}}\end{array}\right)$$	$$\left(\begin{array}{c}0.54{e}^{\left(0.40\right)\pi {{\it i}}},\\ 0.35{e}^{\left(0.35\right)\pi {{\it i}}},\\ 0.43{e}^{\left(0.49\right)\pi {{\it i}}}\end{array}\right)$$
$\left({s}_{3},{s}_{2}\right)$	$$\left(\begin{array}{c}0.55{e}^{(0.34)\pi {{\it i}}},\\ 0.45{e}^{(0.39)\pi {{\it i}}},\\ 0.37{e}^{\left(0.40\right)\pi {{\it i}}}\end{array}\right)$$	$$\left(\begin{array}{c}0.51{e}^{\left(0.40\right)\pi {{\it i}}},\\ 0.49{e}^{\left(0.33\right)\pi {{\it i}}},\\ 0.41{e}^{\left(0.40\right)\pi {{\it i}}}\end{array}\right)$$	$$\left(\begin{array}{c}0.53{e}^{\left(0.41\right)\pi {{\it i}}},\\ 0.42{e}^{\left(0.39\right)\pi {{\it i}}},\\ 0.38{e}^{\left(0.39\right)\pi {{\it i}}}\end{array}\right)$$	$$\left(\begin{array}{c}0.54{e}^{\left(0.41\right)\pi {{\it i}}},\\ 0.35{e}^{\left(0.41\right)\pi {{\it i}}},\\ 0.45{e}^{\left(0.51\right)\pi {{\it i}}}\end{array}\right)$$
$\left({s}_{3},{s}_{3}\right)$	$$\left(\begin{array}{c}0.55{e}^{(0.34)\pi {{\it i}}},\\ 0.45{e}^{(0.39)\pi {{\it i}}},\\ 0.40{e}^{\left(0.40\right)\pi {{\it i}}}\end{array}\right)$$	$$\left(\begin{array}{c}0.52{e}^{\left(0.39\right)\pi {{\it i}}},\\ 0.39{e}^{\left(0.33\right)\pi {{\it i}}},\\ 0.41{e}^{\left(0.45\right)\pi {{\it i}}}\end{array}\right)$$	$$\left(\begin{array}{c}0.53{e}^{\left(0.45\right)\pi {{\it i}}},\\ 0.41{e}^{\left(0.46\right)\pi {{\it i}}},\\ 0.39{e}^{\left(0.40\right)\pi {{\it i}}}\end{array}\right)$$	$$\left(\begin{array}{c}0.54{e}^{\left(0.46\right)\pi {{\it i}}},\\ 0.51{e}^{\left(0.41\right)\pi {{\it i}}},\\ 0.43{e}^{\left(0.46\right)\pi {{\it i}}}\end{array}\right)$$

### Definition 13

The CSFSRs denoted by $\left({\raisebox{-5pt}{\rm H}{\hspace*{-9pt}\neg}}, \raisebox{-2pt}{,}{\hspace*{-9pt}{\rm C}}\right)$ is a subset of any CP of two CSFSSs, where $\raisebox{-2pt}{,}{\hspace*{-9pt}{\rm C}} \subseteq {\acute{\text{\AA}}}\times {B}$ and $\raisebox{-4.5pt}{{\rm R}}\rotatebox{45}{\hspace*{-11pt}--}\left({\acute{\text{\AA}}},{B}\right)\subseteq \left(F, {\acute{\text{\AA}}}\right)\times (\c{G}, {B}), \forall {\acute{\text{\AA}}}\times {B} \in \raisebox{-2pt}{,}{\hspace*{-9pt}{\rm C}}.$

### Example 5

Choose a subset of the CP from Table 1. Then the CSFSR $\raisebox{-4.5pt}{{\rm R}}\rotatebox{45}{\hspace*{-11pt}--}$ is

$$\raisebox{-4.5pt}{{\rm R}}\rotatebox{45}{\hspace*{-11pt}--}=\left\{\begin{array}{c}\left(\left({s}_{1},{s}_{1}\right),\left(\begin{array}{c}0.44{e}^{\left(0.29\right)\pi {{\it i}}},\\ 0.35{e}^{\left(0.30\right)\pi {{\it i}}},\\ 0.34{e}^{\left(0.40\right)\pi {{\it i}}}\end{array}\right),\left(\begin{array}{c}0.48{e}^{\left(0.41\right)\pi {{\it i}}},\\ 0.41{e}^{\left(0.40\right)\pi {{\it i}}},\\ 0.39{e}^{\left(0.45\right)\pi {{\it i}}}\end{array}\right),\left(\begin{array}{c}0.52{e}^{\left(0.41\right)\pi {{\it i}}},\\ 0.53{e}^{\left(0.33\right)\pi {{\it i}}},\\ 0.51{e}^{\left(0.48\right)\pi {{\it i}}}\end{array}\right),\left(\begin{array}{c}0.51{e}^{\left(0.30\right)\pi {{\it i}}},\\ 0.35{e}^{\left(0.29\right)\pi {{\it i}}},\\ 0.43{e}^{\left(0.49\right)\pi {{\it i}}}\end{array}\right)\right),\\ \left(\left({s}_{2},{s}_{1}\right),\left(\begin{array}{c}0.48{e}^{\left(0.31\right)\pi {{\it i}}},\\ 0.35{e}^{\left(0.23\right)\pi {{\it i}}},\\ 0.34{e}^{\left(0.40\right)\pi {{\it i}}}\end{array}\right),\left(\begin{array}{c}0.49{e}^{\left(0.23\right)\pi {{\it i}}},\\ 0.47{e}^{\left(0.39\right)\pi {{\it i}}},\\ 0.39{e}^{\left(0.45\right)\pi {{\it i}}}\end{array}\right),\left(\begin{array}{c}0.46{e}^{\left(0.34\right)\pi {{\it i}}},\\ 0.35{e}^{\left(0.40\right)\pi {{\it i}}},\\ 0.51{e}^{\left(0.39\right)\pi {{\it i}}}\end{array}\right),\left(\begin{array}{c}0.49{e}^{\left(0.36\right)\pi {{\it i}}},\\ 0.35{e}^{\left(0.35\right)\pi {{\it i}}},\\ 0.43{e}^{\left(0.49\right)\pi {{\it i}}}\end{array}\right)\right),\\ \left(\left({s}_{2,}{s}_{2}\right),\left(\begin{array}{c}0.48{e}^{\left(0.31\right)\pi {{\it i}}},\\ 0.49{e}^{\left(0.23\right)\pi {{\it i}}},\\ 0.37{e}^{\left(0.40\right)\pi {{\it i}}}\end{array}\right),\left(\begin{array}{c}0.49{e}^{\left(0.23\right)\pi {{\it i}}},\\ 0.49{e}^{\left(0.38\right)\pi {{\it i}}},\\ 0.41{e}^{\left(0.40\right)\pi {{\it i}}}\end{array}\right),\left(\begin{array}{c}0.46{e}^{\left(0.34\right)\pi {{\it i}}},\\ 0.35{e}^{\left(0.39\right)\pi {{\it i}}},\\ 0.38{e}^{\left(0.39\right)\pi {{\it i}}}\end{array}\right),\left(\begin{array}{c}0.49{e}^{\left(0.36\right)\pi {{\it i}}},\\ 0.35{e}^{\left(0.47\right)\pi {{\it i}}},\\ 0.45{e}^{\left(0.51\right)\pi {{\it i}}}\end{array}\right)\right),\\ \left(\left({s}_{3},{s}_{1}\right),\left(\begin{array}{c}0.49{e}^{\left(0.34\right)\pi {{\it i}}},\\ 0.35{e}^{\left(0.39\right)\pi {{\it i}}},\\ 0.35{e}^{\left(0.40\right)\pi {{\it i}}}\end{array}\right),\left(\begin{array}{c}0.58{e}^{\left(0.41\right)\pi {{\it i}}},\\ 0.47{e}^{\left(0.33\right)\pi {{\it i}}},\\ 0.41{e}^{\left(0.45\right)\pi {{\it i}}}\end{array}\right),\left(\begin{array}{c}0.52{e}^{\left(0.41\right)\pi {{\it i}}},\\ 0.42{e}^{\left(0.40\right)\pi {{\it i}}},\\ 0.51{e}^{\left(0.40\right)\pi {{\it i}}}\end{array}\right),\left(\begin{array}{c}0.54{e}^{\left(0.40\right)\pi {{\it i}}},\\ 0.35{e}^{\left(0.35\right)\pi {{\it i}}},\\ 0.43{e}^{\left(0.49\right)\pi {{\it i}}}\end{array}\right)\right),\\ \left(\left({s}_{3},{s}_{2}\right),\left(\begin{array}{c}0.55{e}^{(0.34)\pi {{\it i}}},\\ 0.45{e}^{(0.39)\pi {{\it i}}},\\ 0.37{e}^{\left(0.40\right)\pi {{\it i}}}\end{array}\right),\left(\begin{array}{c}0.51{e}^{\left(0.40\right)\pi {{\it i}}},\\ 0.49{e}^{\left(0.33\right)\pi {{\it i}}},\\ 0.41{e}^{\left(0.40\right)\pi {{\it i}}}\end{array}\right),\left(\begin{array}{c}0.53{e}^{\left(0.41\right)\pi {{\it i}}},\\ 0.42{e}^{\left(0.39\right)\pi {{\it i}}},\\ 0.38{e}^{\left(0.39\right)\pi {{\it i}}}\end{array}\right),\left(\begin{array}{c}0.54{e}^{\left(0.41\right)\pi {{\it i}}},\\ 0.35{e}^{\left(0.41\right)\pi {{\it i}}},\\ 0.45{e}^{\left(0.51\right)\pi {{\it i}}}\end{array}\right)\right)\end{array}\right\}.$$

### Definition 14

Suppose that $(F, {\acute{\text{\AA}}})$ is a CSFSS on $X$ and,

$$\raisebox{-4.5pt}{{\rm R}}\rotatebox{45}{\hspace*{-11pt}--}=\left(\left(\left(s,f\right)[{{\text{y}}}_{{\psi }_{{\acute{\text{\AA}}}}}\left(s,f\right)]{e}^{{x}_{{\psi }_{{\acute{\text{\AA}}}}}\left(s,f\right){\varvec{\pi}}{{\it i}}},{\left(s,f\right)[{\text{y}}}_{{\mu }_{{\acute{\text{\AA}}}}}\left(s,f\right)]{e}^{{x}_{{\mu }_{{\acute{\text{\AA}}}}}\left(s,f\right){\varvec{\pi}}{{\it i}}},\left(s,f\right)[{{\text{y}}}_{{\eta }_{{\acute{\text{\AA}}}}}\left(s,f\right)]{e}^{{x}_{{\eta }_{{\acute{\text{\AA}}}}}\left(s,f\right){\varvec{\pi}}{{\it i}}}\right)\left(s,f\right)\in \raisebox{-4.5pt}{{\rm R}}\rotatebox{45}{\hspace*{-11pt}--}\right),$$

is an CTSFSR on $\left(F, {\acute{\text{\AA}}}\right).$ Then the inverse of CSFSR is denoted by ${\raisebox{-4.5pt}{{\rm R}}\rotatebox{45}{\hspace*{-11pt}--}}^{-1}$ and is defined as

$${\raisebox{-4.5pt}{{\rm R}}\rotatebox{45}{\hspace*{-11pt}--}}^{-1}=\left(\left(\left(f,s\right)[{{\text{y}}}_{{\psi }_{{\acute{\text{\AA}}}}}\left(f,s\right)]{e}^{{x}_{{\psi }_{{\acute{\text{\AA}}}}}\left(f,s\right){\varvec{\pi}}{{\it i}}},{\left(f,s\right)[{\text{y}}}_{{\mu }_{{\acute{\text{\AA}}}}}\left(f,s\right)]{e}^{{x}_{{\mu }_{{\acute{\text{\AA}}}}}\left(f,s\right){\varvec{\pi}}{{\it i}}},\left(f,s\right)[{{\text{y}}}_{{\eta }_{{\acute{\text{\AA}}}}}\left(f,s\right)]{e}^{{x}_{{\eta }_{{\acute{\text{\AA}}}}}\left(f,s\right){\varvec{\pi}}{{\it i}}}\right)\left(f,s\right)\in {\raisebox{-4.5pt}{{\rm R}}\rotatebox{45}{\hspace*{-11pt}--}}^{-1}\right).$$

### Example 6

Choose a relation from Table 1.

$$\raisebox{-4.5pt}{{\rm R}}\rotatebox{45}{\hspace*{-11pt}--}=\left\{\begin{array}{c}\left(\left({s}_{1},{s}_{1}\right),\left(\begin{array}{c}0.44{e}^{\left(0.29\right)\pi {{\it i}}},\\ 0.35{e}^{\left(0.30\right)\pi {{\it i}}},\\ 0.34{e}^{\left(0.40\right)\pi {{\it i}}}\end{array}\right),\left(\begin{array}{c}0.48{e}^{\left(0.41\right)\pi {{\it i}}},\\ 0.41{e}^{\left(0.40\right)\pi {{\it i}}},\\ 0.39{e}^{\left(0.45\right)\pi {{\it i}}}\end{array}\right),\left(\begin{array}{c}0.52{e}^{\left(0.41\right)\pi {{\it i}}},\\ 0.53{e}^{\left(0.33\right)\pi {{\it i}}},\\ 0.51{e}^{\left(0.48\right)\pi {{\it i}}}\end{array}\right),\left(\begin{array}{c}0.51{e}^{\left(0.30\right)\pi {{\it i}}},\\ 0.35{e}^{\left(0.29\right)\pi {{\it i}}},\\ 0.43{e}^{\left(0.49\right)\pi {{\it i}}}\end{array}\right)\right),\\ \left(\left({s}_{2},{s}_{1}\right),\left(\begin{array}{c}0.48{e}^{\left(0.31\right)\pi {{\it i}}},\\ 0.35{e}^{\left(0.23\right)\pi {{\it i}}},\\ 0.34{e}^{\left(0.40\right)\pi {{\it i}}}\end{array}\right),\left(\begin{array}{c}0.49{e}^{\left(0.23\right)\pi {{\it i}}},\\ 0.47{e}^{\left(0.39\right)\pi {{\it i}}},\\ 0.39{e}^{\left(0.45\right)\pi {{\it i}}}\end{array}\right),\left(\begin{array}{c}0.46{e}^{\left(0.34\right)\pi {{\it i}}},\\ 0.35{e}^{\left(0.40\right)\pi {{\it i}}},\\ 0.51{e}^{\left(0.39\right)\pi {{\it i}}}\end{array}\right),\left(\begin{array}{c}0.49{e}^{\left(0.36\right)\pi {{\it i}}},\\ 0.35{e}^{\left(0.35\right)\pi {{\it i}}},\\ 0.43{e}^{\left(0.49\right)\pi {{\it i}}}\end{array}\right)\right),\\ \left(\left({s}_{2,}{s}_{2}\right),\left(\begin{array}{c}0.48{e}^{\left(0.31\right)\pi {{\it i}}},\\ 0.49{e}^{\left(0.23\right)\pi {{\it i}}},\\ 0.37{e}^{\left(0.40\right)\pi {{\it i}}}\end{array}\right),\left(\begin{array}{c}0.49{e}^{\left(0.23\right)\pi {{\it i}}},\\ 0.49{e}^{\left(0.38\right)\pi {{\it i}}},\\ 0.41{e}^{\left(0.40\right)\pi {{\it i}}}\end{array}\right),\left(\begin{array}{c}0.46{e}^{\left(0.34\right)\pi {{\it i}}},\\ 0.35{e}^{\left(0.39\right)\pi {{\it i}}},\\ 0.38{e}^{\left(0.39\right)\pi {{\it i}}}\end{array}\right),\left(\begin{array}{c}0.49{e}^{\left(0.36\right)\pi {{\it i}}},\\ 0.35{e}^{\left(0.47\right)\pi {{\it i}}},\\ 0.45{e}^{\left(0.51\right)\pi {{\it i}}}\end{array}\right)\right),\\ \left(\left({s}_{3},{s}_{1}\right),\left(\begin{array}{c}0.49{e}^{\left(0.34\right)\pi {{\it i}}},\\ 0.35{e}^{\left(0.39\right)\pi {{\it i}}},\\ 0.35{e}^{\left(0.40\right)\pi {{\it i}}}\end{array}\right),\left(\begin{array}{c}0.58{e}^{\left(0.41\right)\pi {{\it i}}},\\ 0.47{e}^{\left(0.33\right)\pi {{\it i}}},\\ 0.41{e}^{\left(0.45\right)\pi {{\it i}}}\end{array}\right),\left(\begin{array}{c}0.52{e}^{\left(0.41\right)\pi {{\it i}}},\\ 0.42{e}^{\left(0.40\right)\pi {{\it i}}},\\ 0.51{e}^{\left(0.40\right)\pi {{\it i}}}\end{array}\right),\left(\begin{array}{c}0.54{e}^{\left(0.40\right)\pi {{\it i}}},\\ 0.35{e}^{\left(0.35\right)\pi {{\it i}}},\\ 0.43{e}^{\left(0.49\right)\pi {{\it i}}}\end{array}\right)\right),\\ \left(\left({s}_{3},{s}_{2}\right),\left(\begin{array}{c}0.55{e}^{(0.34)\pi {{\it i}}},\\ 0.45{e}^{(0.39)\pi {{\it i}}},\\ 0.37{e}^{\left(0.40\right)\pi {{\it i}}}\end{array}\right),\left(\begin{array}{c}0.51{e}^{\left(0.40\right)\pi {{\it i}}},\\ 0.49{e}^{\left(0.33\right)\pi {{\it i}}},\\ 0.41{e}^{\left(0.40\right)\pi {{\it i}}}\end{array}\right),\left(\begin{array}{c}0.53{e}^{\left(0.41\right)\pi {{\it i}}},\\ 0.42{e}^{\left(0.39\right)\pi {{\it i}}},\\ 0.38{e}^{\left(0.39\right)\pi {{\it i}}}\end{array}\right),\left(\begin{array}{c}0.54{e}^{\left(0.41\right)\pi {{\it i}}},\\ 0.35{e}^{\left(0.41\right)\pi {{\it i}}},\\ 0.45{e}^{\left(0.51\right)\pi {{\it i}}}\end{array}\right)\right)\end{array}\right\}.$$

Then the inverse relation ${\raisebox{-4.5pt}{{\rm R}}\rotatebox{45}{\hspace*{-11pt}--}}^{-1}$ of $\raisebox{-4.5pt}{{\rm R}}\rotatebox{45}{\hspace*{-11pt}--}$ is,

$${\raisebox{-4.5pt}{{\rm R}}\rotatebox{45}{\hspace*{-11pt}--}}^{-1}=\left\{\begin{array}{c}\left(\left({s}_{1},{s}_{1}\right),\left(\begin{array}{c}0.44{e}^{\left(0.29\right)\pi {{\it i}}},\\ 0.35{e}^{\left(0.30\right)\pi {{\it i}}},\\ 0.34{e}^{\left(0.40\right)\pi {{\it i}}}\end{array}\right),\left(\begin{array}{c}0.48{e}^{\left(0.41\right)\pi {{\it i}}},\\ 0.41{e}^{\left(0.40\right)\pi {{\it i}}},\\ 0.39{e}^{\left(0.45\right)\pi {{\it i}}}\end{array}\right),\left(\begin{array}{c}0.52{e}^{\left(0.41\right)\pi {{\it i}}},\\ 0.53{e}^{\left(0.33\right)\pi {{\it i}}},\\ 0.51{e}^{\left(0.48\right)\pi {{\it i}}}\end{array}\right),\left(\begin{array}{c}0.51{e}^{\left(0.30\right)\pi {{\it i}}},\\ 0.35{e}^{\left(0.29\right)\pi {{\it i}}},\\ 0.43{e}^{\left(0.49\right)\pi {{\it i}}}\end{array}\right)\right),\\ \left(\left({s}_{1},{s}_{2}\right),\left(\begin{array}{c}0.44{e}^{(0.29)\pi {{\it i}}},\\ 0.40{e}^{(0.30)\pi {{\it i}}},\\ 0.37{e}^{\left(0.40\right)\pi {{\it i}}}\end{array}\right),\left(\begin{array}{c}0.48{e}^{\left(0.40\right)\pi {{\it i}}},\\ 0.41{e}^{\left(0.38\right)\pi {{\it i}}},\\ 0.41{e}^{\left(0.40\right)\pi {{\it i}}}\end{array}\right),\left(\begin{array}{c}0.53{e}^{\left(0.41\right)\pi {{\it i}}},\\ 0.52{e}^{\left(0.33\right)\pi {{\it i}}},\\ 0.38{e}^{\left(0.48\right)\pi {{\it i}}}\end{array}\right),\left(\begin{array}{c}0.51{e}^{\left(0.30\right)\pi {{\it i}}},\\ 0.35{e}^{\left(0.29\right)\pi {{\it i}}},\\ 0.45{e}^{\left(0.51\right)\pi {{\it i}}}\end{array}\right)\right),\\ \left(\left({s}_{2,}{s}_{2}\right),\left(\begin{array}{c}0.48{e}^{\left(0.31\right)\pi {{\it i}}},\\ 0.49{e}^{\left(0.23\right)\pi {{\it i}}},\\ 0.37{e}^{\left(0.40\right)\pi {{\it i}}}\end{array}\right),\left(\begin{array}{c}0.49{e}^{\left(0.23\right)\pi {{\it i}}},\\ 0.49{e}^{\left(0.38\right)\pi {{\it i}}},\\ 0.41{e}^{\left(0.40\right)\pi {{\it i}}}\end{array}\right),\left(\begin{array}{c}0.46{e}^{\left(0.34\right)\pi {{\it i}}},\\ 0.35{e}^{\left(0.39\right)\pi {{\it i}}},\\ 0.38{e}^{\left(0.39\right)\pi {{\it i}}}\end{array}\right),\left(\begin{array}{c}0.49{e}^{\left(0.36\right)\pi {{\it i}}},\\ 0.35{e}^{\left(0.47\right)\pi {{\it i}}},\\ 0.45{e}^{\left(0.51\right)\pi {{\it i}}}\end{array}\right)\right),\\ \left(\left({s}_{1},{s}_{3}\right),\left(\begin{array}{c}0.44{e}^{(0.29)\pi {{\it i}}},\\ 0.40{e}^{(0.30)\pi {{\it i}}},\\ 0.40{e}^{\left(0.39\right)\pi {{\it i}}}\end{array}\right),\left(\begin{array}{c}0.48{e}^{\left(0.39\right)\pi {{\it i}}},\\ 0.41{e}^{\left(0.40\right)\pi {{\it i}}},\\ 0.40{e}^{\left(0.45\right)\pi {{\it i}}}\end{array}\right),\left(\begin{array}{c}0.53{e}^{\left(0.45\right)\pi {{\it i}}},\\ 0.41{e}^{\left(0.33\right)\pi {{\it i}}},\\ 0.38{e}^{\left(0.48\right)\pi {{\it i}}}\end{array}\right),\left(\begin{array}{c}0.51{e}^{\left(0.30\right)\pi {{\it i}}},\\ 0.49{e}^{\left(0.29\right)\pi {{\it i}}},\\ 0.43{e}^{\left(0.46\right)\pi {{\it i}}}\end{array}\right)\right),\\ \left(\left({s}_{2},{s}_{3}\right),\left(\begin{array}{c}0.48{e}^{(0.31)\pi {{\it i}}},\\ 0.46{e}^{(0.23)\pi {{\it i}}},\\ 0.40{e}^{\left(0.39\right)\pi {{\it i}}}\end{array}\right),\left(\begin{array}{c}0.49{e}^{\left(0.23\right)\pi {{\it i}}},\\ 0.54{e}^{\left(0.39\right)\pi {{\it i}}},\\ 0.40{e}^{\left(0.45\right)\pi {{\it i}}}\end{array}\right),\left(\begin{array}{c}0.46{e}^{\left(0.34\right)\pi {{\it i}}},\\ 0.35{e}^{\left(0.46\right)\pi {{\it i}}},\\ 0.38{e}^{\left(0.40\right)\pi {{\it i}}}\end{array}\right),\left(\begin{array}{c}0.49{e}^{\left(0.36\right)\pi {{\it i}}},\\ 0.43{e}^{\left(0.41\right)\pi {{\it i}}},\\ 0.43{e}^{\left(0.48\right)\pi {{\it i}}}\end{array}\right)\right)\end{array}\right\}.$$

### Definition 15

A CSFSR $\raisebox{-4.5pt}{{\rm R}}\rotatebox{45}{\hspace*{-11pt}--}$ on $F$ is called CSFS-reflexive-R if

$$\forall \left(s,{{\psi }_{{\acute{\text{\AA}}}}}_{c}\left(s\right),{{\mu }_{{\acute{\text{\AA}}}}}_{c}\left(s\right),{{\eta }_{{\acute{\text{\AA}}}}}_{c}\left(s\right)\right)\in F,$$

implies that for all $\left(\left(s,s\right),{{\psi }_{{{{\acute{\text{\AA}}}}}}}_{c}\left(s,s\right),{{\mu }_{{{{\acute{\text{\AA}}}}}}}_{c}\left(s,s\right),{{\eta }_{{{{\acute{\text{\AA}}}}}}}_{c}\left(s,s\right)\right)\in \raisebox{-4.5pt}{{\rm R}}\rotatebox{45}{\hspace*{-11pt}--}$.

### Example 7

Table 1 shows the CP of CSFSRs. The CSFS-reflexive-R is,

$$\raisebox{-4.5pt}{{\rm R}}\rotatebox{45}{\hspace*{-11pt}--}=\left\{\begin{array}{c}\left(\left({s}_{1},{s}_{1}\right),\left(\begin{array}{c}0.44{e}^{\left(0.29\right)\pi {{\it i}}},\\ 0.35{e}^{\left(0.30\right)\pi {{\it i}}},\\ 0.34{e}^{\left(0.40\right)\pi {{\it i}}}\end{array}\right),\left(\begin{array}{c}0.48{e}^{\left(0.41\right)\pi {{\it i}}},\\ 0.41{e}^{\left(0.40\right)\pi {{\it i}}},\\ 0.39{e}^{\left(0.45\right)\pi {{\it i}}}\end{array}\right),\left(\begin{array}{c}0.52{e}^{\left(0.41\right)\pi {{\it i}}},\\ 0.53{e}^{\left(0.33\right)\pi {{\it i}}},\\ 0.51{e}^{\left(0.48\right)\pi {{\it i}}}\end{array}\right),\left(\begin{array}{c}0.51{e}^{\left(0.30\right)\pi {{\it i}}},\\ 0.35{e}^{\left(0.29\right)\pi {{\it i}}},\\ 0.43{e}^{\left(0.49\right)\pi {{\it i}}}\end{array}\right)\right),\\ \left(\left({s}_{2,}{s}_{2}\right),\left(\begin{array}{c}0.48{e}^{\left(0.31\right)\pi {{\it i}}},\\ 0.49{e}^{\left(0.23\right)\pi {{\it i}}},\\ 0.37{e}^{\left(0.40\right)\pi {{\it i}}}\end{array}\right),\left(\begin{array}{c}0.49{e}^{\left(0.23\right)\pi {{\it i}}},\\ 0.49{e}^{\left(0.38\right)\pi {{\it i}}},\\ 0.41{e}^{\left(0.40\right)\pi {{\it i}}}\end{array}\right),\left(\begin{array}{c}0.46{e}^{\left(0.34\right)\pi {{\it i}}},\\ 0.35{e}^{\left(0.39\right)\pi {{\it i}}},\\ 0.38{e}^{\left(0.39\right)\pi {{\it i}}}\end{array}\right),\left(\begin{array}{c}0.49{e}^{\left(0.36\right)\pi {{\it i}}},\\ 0.35{e}^{\left(0.47\right)\pi {{\it i}}},\\ 0.45{e}^{\left(0.51\right)\pi {{\it i}}}\end{array}\right)\right),\\ \left(\left({s}_{3},{s}_{3}\right),\left(\begin{array}{c}0.55{e}^{(0.34)\pi {{\it i}}},\\ 0.45{e}^{(0.39)\pi {{\it i}}},\\ 0.40{e}^{\left(0.40\right)\pi {{\it i}}}\end{array}\right),\left(\begin{array}{c}0.52{e}^{\left(0.39\right)\pi {{\it i}}},\\ 0.39{e}^{\left(0.33\right)\pi {{\it i}}},\\ 0.41{e}^{\left(0.45\right)\pi {{\it i}}}\end{array}\right),\left(\begin{array}{c}0.53{e}^{\left(0.45\right)\pi {{\it i}}},\\ 0.41{e}^{\left(0.46\right)\pi {{\it i}}},\\ 0.39{e}^{\left(0.40\right)\pi {{\it i}}}\end{array}\right),\left(\begin{array}{c}0.54{e}^{\left(0.46\right)\pi {{\it i}}},\\ 0.51{e}^{\left(0.41\right)\pi {{\it i}}},\\ 0.43{e}^{\left(0.46\right)\pi {{\it i}}}\end{array}\right)\right),\\ \left(\left({s}_{3},{s}_{1}\right),\left(\begin{array}{c}0.49{e}^{\left(0.34\right)\pi {{\it i}}},\\ 0.35{e}^{\left(0.39\right)\pi {{\it i}}},\\ 0.35{e}^{\left(0.40\right)\pi {{\it i}}}\end{array}\right),\left(\begin{array}{c}0.58{e}^{\left(0.41\right)\pi {{\it i}}},\\ 0.47{e}^{\left(0.33\right)\pi {{\it i}}},\\ 0.41{e}^{\left(0.45\right)\pi {{\it i}}}\end{array}\right),\left(\begin{array}{c}0.52{e}^{\left(0.41\right)\pi {{\it i}}},\\ 0.42{e}^{\left(0.40\right)\pi {{\it i}}},\\ 0.51{e}^{\left(0.40\right)\pi {{\it i}}}\end{array}\right),\left(\begin{array}{c}0.54{e}^{\left(0.40\right)\pi {{\it i}}},\\ 0.35{e}^{\left(0.35\right)\pi {{\it i}}},\\ 0.43{e}^{\left(0.49\right)\pi {{\it i}}}\end{array}\right)\right),\\ \left(\left({s}_{2},{s}_{3}\right),\left(\begin{array}{c}0.48{e}^{(0.31)\pi {{\it i}}},\\ 0.46{e}^{(0.23)\pi {{\it i}}},\\ 0.40{e}^{\left(0.39\right)\pi {{\it i}}}\end{array}\right),\left(\begin{array}{c}0.49{e}^{\left(0.23\right)\pi {{\it i}}},\\ 0.54{e}^{\left(0.39\right)\pi {{\it i}}},\\ 0.40{e}^{\left(0.45\right)\pi {{\it i}}}\end{array}\right),\left(\begin{array}{c}0.46{e}^{\left(0.34\right)\pi {{\it i}}},\\ 0.35{e}^{\left(0.46\right)\pi {{\it i}}},\\ 0.38{e}^{\left(0.40\right)\pi {{\it i}}}\end{array}\right),\left(\begin{array}{c}0.49{e}^{\left(0.36\right)\pi {{\it i}}},\\ 0.43{e}^{\left(0.41\right)\pi {{\it i}}},\\ 0.43{e}^{\left(0.48\right)\pi {{\it i}}}\end{array}\right)\right)\end{array}\right\}.$$

### Definition 16

A CSFSR $\raisebox{-4.5pt}{{\rm R}}\rotatebox{45}{\hspace*{-11pt}--}$ on $F$ is called CSFS-irreflexive-R if

$$\forall \left(s,{{\psi }_{{\acute{\text{\AA}}}}}_{c}\left(s\right),{{\mu }_{{\acute{\text{\AA}}}}}_{c}\left(s\right),{{\eta }_{{\acute{\text{\AA}}}}}_{c}\left(s\right)\right)\in F.$$

Implies that $$\forall \left(\left(s,s\right),{{\psi }_{{\acute{\text{\AA}}}}}_{c}\left(s,s\right),{{\mu }_{{\acute{\text{\AA}}}}}_{c}\left(s,s\right),{{\eta }_{{\acute{\text{\AA}}}}}_{c}\left(s,s\right)\right)\notin \raisebox{-4.5pt}{{\rm R}}\rotatebox{45}{\hspace*{-11pt}--}$$.

### Example 8

Table 1 shows the CP of CSFSR. The CSFS-irreflexive-R is,

$$\raisebox{-4.5pt}{{\rm R}}\rotatebox{45}{\hspace*{-11pt}--}=\left\{\begin{array}{c}\left(\left({s}_{1},{s}_{3}\right),\left(\begin{array}{c}0.44{e}^{(0.29)\pi {{\it i}}},\\ 0.40{e}^{(0.30)\pi {{\it i}}},\\ 0.40{e}^{\left(0.39\right)\pi {{\it i}}}\end{array}\right),\left(\begin{array}{c}0.48{e}^{\left(0.39\right)\pi {{\it i}}},\\ 0.41{e}^{\left(0.40\right)\pi {{\it i}}},\\ 0.40{e}^{\left(0.45\right)\pi {{\it i}}}\end{array}\right),\left(\begin{array}{c}0.53{e}^{\left(0.45\right)\pi {{\it i}}},\\ 0.41{e}^{\left(0.33\right)\pi {{\it i}}},\\ 0.38{e}^{\left(0.48\right)\pi {{\it i}}}\end{array}\right),\left(\begin{array}{c}0.51{e}^{\left(0.30\right)\pi {{\it i}}},\\ 0.49{e}^{\left(0.29\right)\pi {{\it i}}},\\ 0.43{e}^{\left(0.46\right)\pi {{\it i}}}\end{array}\right)\right),\\ \left(\left({s}_{2},{s}_{3}\right),\left(\begin{array}{c}0.48{e}^{(0.31)\pi {{\it i}}},\\ 0.46{e}^{(0.23)\pi {{\it i}}},\\ 0.40{e}^{\left(0.39\right)\pi {{\it i}}}\end{array}\right),\left(\begin{array}{c}0.49{e}^{\left(0.23\right)\pi {{\it i}}},\\ 0.54{e}^{\left(0.39\right)\pi {{\it i}}},\\ 0.40{e}^{\left(0.45\right)\pi {{\it i}}}\end{array}\right),\left(\begin{array}{c}0.46{e}^{\left(0.34\right)\pi {{\it i}}},\\ 0.35{e}^{\left(0.46\right)\pi {{\it i}}},\\ 0.38{e}^{\left(0.40\right)\pi {{\it i}}}\end{array}\right),\left(\begin{array}{c}0.49{e}^{\left(0.36\right)\pi {{\it i}}},\\ 0.43{e}^{\left(0.41\right)\pi {{\it i}}},\\ 0.43{e}^{\left(0.48\right)\pi {{\it i}}}\end{array}\right)\right),\\ \left(\left({s}_{1},{s}_{2}\right),\left(\begin{array}{c}0.44{e}^{(0.29)\pi {{\it i}}},\\ 0.40{e}^{(0.30)\pi {{\it i}}},\\ 0.37{e}^{\left(0.40\right)\pi {{\it i}}}\end{array}\right),\left(\begin{array}{c}0.48{e}^{\left(0.40\right)\pi {{\it i}}},\\ 0.41{e}^{\left(0.38\right)\pi {{\it i}}},\\ 0.41{e}^{\left(0.40\right)\pi {{\it i}}}\end{array}\right),\left(\begin{array}{c}0.53{e}^{\left(0.41\right)\pi {{\it i}}},\\ 0.52{e}^{\left(0.33\right)\pi {{\it i}}},\\ 0.38{e}^{\left(0.48\right)\pi {{\it i}}}\end{array}\right),\left(\begin{array}{c}0.51{e}^{\left(0.30\right)\pi {{\it i}}},\\ 0.35{e}^{\left(0.29\right)\pi {{\it i}}},\\ 0.45{e}^{\left(0.51\right)\pi {{\it i}}}\end{array}\right)\right)\end{array}\right\}.$$

### Definition 17

A CSFSR $\raisebox{-4.5pt}{{\rm R}}\rotatebox{45}{\hspace*{-11pt}--}$ on $F$ is called CSFS-symmetric-R if

$$\forall \left(s,{{\psi }_{{\acute{\text{\AA}}}}}_{c}\left(s\right),{{\mu }_{{\acute{\text{\AA}}}}}_{c}\left(s\right),{{\eta }_{{\acute{\text{\AA}}}}}_{c}\left(s\right)\right),\left(f,{{\psi }_{{\acute{\text{\AA}}}}}_{c}\left(f\right),{{\mu }_{{\acute{\text{\AA}}}}}_{c}\left(f\right),{{\eta }_{{\acute{\text{\AA}}}}}_{c}\left(f\right)\right)\in F \, \text{and }s,f\in F,$$

Then $$\left(\left(s,f\right),{{\psi }_{{\acute{\text{\AA}}}}}_{c}\left(s,f\right),{{\mu }_{{\acute{\text{\AA}}}}}_{c}\left(s,f\right),{{\eta }_{{\acute{\text{\AA}}}}}_{c}\left(s,f\right)\right)\in \raisebox{-4.5pt}{{\rm R}}\rotatebox{45}{\hspace*{-11pt}--}  \Rightarrow  \left(\left(f,s\right),{{\psi }_{{\acute{\text{\AA}}}}}_{c}\left(f,s\right),{{\mu }_{{\acute{\text{\AA}}}}}_{c}\left(f,s\right),{{\eta }_{{\acute{\text{\AA}}}}}_{c}\left(f,s\right)\right)\in \raisebox{-4.5pt}{{\rm R}}\rotatebox{45}{\hspace*{-11pt}--}$$.

### Example 9

Table 1 shows the CP of CSFSR. The CSFS-symmetric-R is,

$$\raisebox{-4.5pt}{{\rm R}}\rotatebox{45}{\hspace*{-11pt}--}=\left\{\begin{array}{c}\left(\left({s}_{1},{s}_{2}\right),\left(\begin{array}{c}0.44{e}^{(0.29)\pi {{\it i}}},\\ 0.40{e}^{(0.30)\pi {{\it i}}},\\ 0.37{e}^{\left(0.40\right)\pi {{\it i}}}\end{array}\right),\left(\begin{array}{c}0.48{e}^{\left(0.40\right)\pi {{\it i}}},\\ 0.41{e}^{\left(0.38\right)\pi {{\it i}}},\\ 0.41{e}^{\left(0.40\right)\pi {{\it i}}}\end{array}\right),\left(\begin{array}{c}0.53{e}^{\left(0.41\right)\pi {{\it i}}},\\ 0.52{e}^{\left(0.33\right)\pi {{\it i}}},\\ 0.38{e}^{\left(0.48\right)\pi {{\it i}}}\end{array}\right),\left(\begin{array}{c}0.51{e}^{\left(0.30\right)\pi {{\it i}}},\\ 0.35{e}^{\left(0.29\right)\pi {{\it i}}},\\ 0.45{e}^{\left(0.51\right)\pi {{\it i}}}\end{array}\right)\right),\\ \left(\left({s}_{2},{s}_{3}\right),\left(\begin{array}{c}0.48{e}^{(0.31)\pi {{\it i}}},\\ 0.46{e}^{(0.23)\pi {{\it i}}},\\ 0.40{e}^{\left(0.39\right)\pi {{\it i}}}\end{array}\right),\left(\begin{array}{c}0.49{e}^{\left(0.23\right)\pi {{\it i}}},\\ 0.54{e}^{\left(0.39\right)\pi {{\it i}}},\\ 0.40{e}^{\left(0.45\right)\pi {{\it i}}}\end{array}\right),\left(\begin{array}{c}0.46{e}^{\left(0.34\right)\pi {{\it i}}},\\ 0.35{e}^{\left(0.46\right)\pi {{\it i}}},\\ 0.38{e}^{\left(0.40\right)\pi {{\it i}}}\end{array}\right),\left(\begin{array}{c}0.49{e}^{\left(0.36\right)\pi {{\it i}}},\\ 0.43{e}^{\left(0.41\right)\pi {{\it i}}},\\ 0.43{e}^{\left(0.48\right)\pi {{\it i}}}\end{array}\right)\right),\\ \left(\left({s}_{2},{s}_{1}\right),\left(\begin{array}{c}0.48{e}^{\left(0.31\right)\pi {{\it i}}},\\ 0.35{e}^{\left(0.23\right)\pi {{\it i}}},\\ 0.34{e}^{\left(0.40\right)\pi {{\it i}}}\end{array}\right),\left(\begin{array}{c}0.49{e}^{\left(0.23\right)\pi {{\it i}}},\\ 0.47{e}^{\left(0.39\right)\pi {{\it i}}},\\ 0.39{e}^{\left(0.45\right)\pi {{\it i}}}\end{array}\right),\left(\begin{array}{c}0.46{e}^{\left(0.34\right)\pi {{\it i}}},\\ 0.35{e}^{\left(0.40\right)\pi {{\it i}}},\\ 0.51{e}^{\left(0.39\right)\pi {{\it i}}}\end{array}\right),\left(\begin{array}{c}0.49{e}^{\left(0.36\right)\pi {{\it i}}},\\ 0.35{e}^{\left(0.35\right)\pi {{\it i}}},\\ 0.43{e}^{\left(0.49\right)\pi {{\it i}}}\end{array}\right)\right),\\ \left(\left({s}_{1},{s}_{3}\right),\left(\begin{array}{c}0.44{e}^{(0.29)\pi {{\it i}}},\\ 0.40{e}^{(0.30)\pi {{\it i}}},\\ 0.40{e}^{\left(0.39\right)\pi {{\it i}}}\end{array}\right),\left(\begin{array}{c}0.48{e}^{\left(0.39\right)\pi {{\it i}}},\\ 0.41{e}^{\left(0.40\right)\pi {{\it i}}},\\ 0.40{e}^{\left(0.45\right)\pi {{\it i}}}\end{array}\right),\left(\begin{array}{c}0.53{e}^{\left(0.45\right)\pi {{\it i}}},\\ 0.41{e}^{\left(0.33\right)\pi {{\it i}}},\\ 0.38{e}^{\left(0.48\right)\pi {{\it i}}}\end{array}\right),\left(\begin{array}{c}0.51{e}^{\left(0.30\right)\pi {{\it i}}},\\ 0.49{e}^{\left(0.29\right)\pi {{\it i}}},\\ 0.43{e}^{\left(0.46\right)\pi {{\it i}}}\end{array}\right)\right),\\ \left(\left({s}_{3},{s}_{2}\right),\left(\begin{array}{c}0.55{e}^{(0.34)\pi {{\it i}}},\\ 0.45{e}^{(0.39)\pi {{\it i}}},\\ 0.37{e}^{\left(0.40\right)\pi {{\it i}}}\end{array}\right),\left(\begin{array}{c}0.51{e}^{\left(0.40\right)\pi {{\it i}}},\\ 0.49{e}^{\left(0.33\right)\pi {{\it i}}},\\ 0.41{e}^{\left(0.40\right)\pi {{\it i}}}\end{array}\right),\left(\begin{array}{c}0.53{e}^{\left(0.41\right)\pi {{\it i}}},\\ 0.42{e}^{\left(0.39\right)\pi {{\it i}}},\\ 0.38{e}^{\left(0.39\right)\pi {{\it i}}}\end{array}\right),\left(\begin{array}{c}0.54{e}^{\left(0.41\right)\pi {{\it i}}},\\ 0.35{e}^{\left(0.41\right)\pi {{\it i}}},\\ 0.45{e}^{\left(0.51\right)\pi {{\it i}}}\end{array}\right)\right),\\ \left(\left({s}_{3},{s}_{1}\right),\left(\begin{array}{c}0.49{e}^{\left(0.34\right)\pi {{\it i}}},\\ 0.35{e}^{\left(0.39\right)\pi {{\it i}}},\\ 0.35{e}^{\left(0.40\right)\pi {{\it i}}}\end{array}\right),\left(\begin{array}{c}0.58{e}^{\left(0.41\right)\pi {{\it i}}},\\ 0.47{e}^{\left(0.33\right)\pi {{\it i}}},\\ 0.41{e}^{\left(0.45\right)\pi {{\it i}}}\end{array}\right),\left(\begin{array}{c}0.52{e}^{\left(0.41\right)\pi {{\it i}}},\\ 0.42{e}^{\left(0.40\right)\pi {{\it i}}},\\ 0.51{e}^{\left(0.40\right)\pi {{\it i}}}\end{array}\right),\left(\begin{array}{c}0.54{e}^{\left(0.40\right)\pi {{\it i}}},\\ 0.35{e}^{\left(0.35\right)\pi {{\it i}}},\\ 0.43{e}^{\left(0.49\right)\pi {{\it i}}}\end{array}\right)\right)\end{array}\right\}.$$

### Definition 18

A CSFSR $\raisebox{-4.5pt}{{\rm R}}\rotatebox{45}{\hspace*{-11pt}--}$ on $F$ is called CSFS-antisymmetric-R if

$$\forall \left(s,{{\psi }_{{\acute{\text{\AA}}}}}_{c}\left(s\right),{{\mu }_{{\acute{\text{\AA}}}}}_{c}\left(s\right),{{\eta }_{{\acute{\text{\AA}}}}}_{c}\left(s\right)\right),\left(f,{{\psi }_{{\acute{\text{\AA}}}}}_{c}\left(f\right),{{\mu }_{{\acute{\text{\AA}}}}}_{c}\left(f\right),{{\eta }_{{\acute{\text{\AA}}}}}_{c}\left(f\right)\right)\in F,$$

Then $$\left(\left(s,f\right),{{\psi }_{{\acute{\text{\AA}}}}}_{c}\left(s,f\right),{{\mu }_{{\acute{\text{\AA}}}}}_{c}\left(s,f\right),{{\eta }_{{\acute{\text{\AA}}}}}_{c}\left(s,f\right)\right)\in {\raisebox{-4.5pt}{{\rm R}}\rotatebox{45}{\hspace*{-11pt}--}}\,and\,\left(\left(f,s\right),{{\psi }_{{\acute{\text{\AA}}}}}_{c}\left(f,s\right),{{\mu }_{{\acute{\text{\AA}}}}}_{c}\left(f,s\right),{{\eta }_{{\acute{\text{\AA}}}}}_{c}\left(f,s\right)\right)\in \raisebox{-4.5pt}{{\rm R}}\rotatebox{45}{\hspace*{-11pt}--}$$.

$$\Rightarrow \left(\left(s,f\right),{{\psi }_{{\acute{\text{\AA}}}}}_{c}\left(s,f\right),{{\mu }_{{\acute{\text{\AA}}}}}_{c}\left(s,f\right),{{\eta }_{{\acute{\text{\AA}}}}}_{c}\left(s,f\right)\right)=\left(\left(f,s\right),{{\psi }_{{\acute{\text{\AA}}}}}_{c}\left(f,s\right),{{\mu }_{{\acute{\text{\AA}}}}}_{c}\left(f,s\right),{{\eta }_{{\acute{\text{\AA}}}}}_{c}\left(f,s\right)\right).$$

### Example 10

Table 1 shows the CP of CSFSR. The CSFS-anti symmetric-R is,

$$\raisebox{-4.5pt}{{\rm R}}\rotatebox{45}{\hspace*{-11pt}--}=\left\{\begin{array}{c}\left(\left({s}_{1},{s}_{1}\right),\left(\begin{array}{c}0.44{e}^{\left(0.29\right){\varvec{\pi}}{{\it i}}},\\ 0.35{e}^{\left(0.30\right){\varvec{\pi}}{{\it i}}},\\ 0.34{e}^{\left(0.40\right){\varvec{\pi}}{{\it i}}}\end{array}\right),\left(\begin{array}{c}0.48{e}^{\left(0.41\right){\varvec{\pi}}{{\it i}}},\\ 0.41{e}^{\left(0.40\right){\varvec{\pi}}{{\it i}}},\\ 0.39{e}^{\left(0.45\right){\varvec{\pi}}{{\it i}}}\end{array}\right),\left(\begin{array}{c}0.52{e}^{\left(0.41\right){\varvec{\pi}}{{\it i}}},\\ 0.53{e}^{\left(0.33\right){\varvec{\pi}}{{\it i}}},\\ 0.51{e}^{\left(0.48\right){\varvec{\pi}}{{\it i}}}\end{array}\right),\left(\begin{array}{c}0.51{e}^{\left(0.30\right){\varvec{\pi}}{{\it i}}},\\ 0.35{e}^{\left(0.29\right){\varvec{\pi}}{{\it i}}},\\ 0.43{e}^{\left(0.49\right){\varvec{\pi}}{{\it i}}}\end{array}\right)\right),\\ \left(\left({s}_{2,}{s}_{2}\right),\left(\begin{array}{c}0.48{e}^{\left(0.31\right){\varvec{\pi}}{{\it i}}},\\ 0.49{e}^{\left(0.23\right){\varvec{\pi}}{{\it i}}},\\ 0.37{e}^{\left(0.40\right){\varvec{\pi}}{{\it i}}}\end{array}\right),\left(\begin{array}{c}0.49{e}^{\left(0.23\right){\varvec{\pi}}{{\it i}}},\\ 0.49{e}^{\left(0.38\right){\varvec{\pi}}{{\it i}}},\\ 0.41{e}^{\left(0.40\right){\varvec{\pi}}{{\it i}}}\end{array}\right),\left(\begin{array}{c}0.46{e}^{\left(0.34\right){\varvec{\pi}}{{\it i}}},\\ 0.35{e}^{\left(0.39\right){\varvec{\pi}}{{\it i}}},\\ 0.38{e}^{\left(0.39\right){\varvec{\pi}}{{\it i}}}\end{array}\right),\left(\begin{array}{c}0.49{e}^{\left(0.36\right){\varvec{\pi}}{{\it i}}},\\ 0.35{e}^{\left(0.47\right){\varvec{\pi}}{{\it i}}},\\ 0.45{e}^{\left(0.51\right){\varvec{\pi}}{{\it i}}}\end{array}\right)\right),\\ \left(\left({s}_{3},{s}_{3}\right),\left(\begin{array}{c}0.55{e}^{(0.34){\varvec{\pi}}{{\it i}}},\\ 0.45{e}^{(0.39){\varvec{\pi}}{{\it i}}},\\ 0.40{e}^{\left(0.40\right){\varvec{\pi}}{{\it i}}}\end{array}\right),\left(\begin{array}{c}0.52{e}^{\left(0.39\right){\varvec{\pi}}{{\it i}}},\\ 0.39{e}^{\left(0.33\right){\varvec{\pi}}{{\it i}}},\\ 0.41{e}^{\left(0.45\right){\varvec{\pi}}{{\it i}}}\end{array}\right),\left(\begin{array}{c}0.53{e}^{\left(0.45\right){\varvec{\pi}}{{\it i}}},\\ 0.41{e}^{\left(0.46\right){\varvec{\pi}}{{\it i}}},\\ 0.39{e}^{\left(0.40\right){\varvec{\pi}}{{\it i}}}\end{array}\right),\left(\begin{array}{c}0.54{e}^{\left(0.46\right){\varvec{\pi}}{{\it i}}},\\ 0.51{e}^{\left(0.41\right){\varvec{\pi}}{{\it i}}},\\ 0.43{e}^{\left(0.46\right){\varvec{\pi}}{{\it i}}}\end{array}\right)\right)\end{array}\right\}.$$

### Definition 19

A CSFSR $\raisebox{-4.5pt}{{\rm R}}\rotatebox{45}{\hspace*{-11pt}--}$ on $F$ is called CSFS-asymmetric-R if

$$\forall \left(s,{{\psi }_{{\acute{\text{\AA}}}}}_{c}\left(s\right),{{\mu }_{{\acute{\text{\AA}}}}}_{c}\left(s\right),{{\eta }_{{\acute{\text{\AA}}}}}_{c}\left(s\right)\right),\left(f,{{\psi }_{{\acute{\text{\AA}}}}}_{c}\left(f\right),{{\mu }_{{\acute{\text{\AA}}}}}_{c}\left(f\right),{{\eta }_{{\acute{\text{\AA}}}}}_{c}\left(f\right)\right)\in F.$$

Then $$\left(\left(s,f\right),{{\psi }_{{\acute{\text{\AA}}}}}_{c}\left(s,f\right),{{\mu }_{{\acute{\text{\AA}}}}}_{c}\left(s,f\right),{{\eta }_{{\acute{\text{\AA}}}}}_{c}\left(s,f\right)\right)\in \raisebox{-4.5pt}{{\rm R}}\rotatebox{45}{\hspace*{-11pt}--}  \Rightarrow  \left(\left(f,s\right),{{\psi }_{{\acute{\text{\AA}}}}}_{c}\left(f,s\right),{{\mu }_{{\acute{\text{\AA}}}}}_{c}\left(f,s\right),{{\eta }_{{\acute{\text{\AA}}}}}_{c}\left(f,s\right)\right)\notin \raisebox{-4.5pt}{{\rm R}}\rotatebox{45}{\hspace*{-11pt}--}$$.

### Example 11

Table 1 shows the CP of CSFSR. The CSFS-asymmetric-R is

$$\raisebox{-4.5pt}{{\rm R}}\rotatebox{45}{\hspace*{-11pt}--}=\left\{\begin{array}{c}\left(\left({s}_{1},{s}_{2}\right),\left(\begin{array}{c}0.44{e}^{(0.29){\varvec{\pi}}{{\it i}}},\\ 0.40{e}^{(0.30){\varvec{\pi}}{{\it i}}},\\ 0.37{e}^{\left(0.40\right){\varvec{\pi}}{{\it i}}}\end{array}\right),\left(\begin{array}{c}0.48{e}^{\left(0.40\right){\varvec{\pi}}{{\it i}}},\\ 0.41{e}^{\left(0.38\right){\varvec{\pi}}{{\it i}}},\\ 0.41{e}^{\left(0.40\right){\varvec{\pi}}{{\it i}}}\end{array}\right),\left(\begin{array}{c}0.53{e}^{\left(0.41\right){\varvec{\pi}}{{\it i}}},\\ 0.52{e}^{\left(0.33\right){\varvec{\pi}}{{\it i}}},\\ 0.38{e}^{\left(0.48\right){\varvec{\pi}}{{\it i}}}\end{array}\right),\left(\begin{array}{c}0.51{e}^{\left(0.30\right){\varvec{\pi}}{{\it i}}},\\ 0.35{e}^{\left(0.29\right){\varvec{\pi}}{{\it i}}},\\ 0.45{e}^{\left(0.51\right){\varvec{\pi}}{{\it i}}}\end{array}\right)\right),\\ \left(\left({s}_{1},{s}_{3}\right),\left(\begin{array}{c}0.44{e}^{(0.29){\varvec{\pi}}{{\it i}}},\\ 0.40{e}^{(0.30){\varvec{\pi}}{{\it i}}},\\ 0.40{e}^{\left(0.39\right){\varvec{\pi}}{{\it i}}}\end{array}\right),\left(\begin{array}{c}0.48{e}^{\left(0.39\right){\varvec{\pi}}{{\it i}}},\\ 0.41{e}^{\left(0.40\right){\varvec{\pi}}{{\it i}}},\\ 0.40{e}^{\left(0.45\right){\varvec{\pi}}{{\it i}}}\end{array}\right),\left(\begin{array}{c}0.53{e}^{\left(0.45\right){\varvec{\pi}}{{\it i}}},\\ 0.41{e}^{\left(0.33\right){\varvec{\pi}}{{\it i}}},\\ 0.38{e}^{\left(0.48\right){\varvec{\pi}}{{\it i}}}\end{array}\right),\left(\begin{array}{c}0.51{e}^{\left(0.30\right){\varvec{\pi}}{{\it i}}},\\ 0.49{e}^{\left(0.29\right){\varvec{\pi}}{{\it i}}},\\ 0.43{e}^{\left(0.46\right){\varvec{\pi}}{{\it i}}}\end{array}\right)\right),\\ \left(\left({s}_{2},{s}_{3}\right),\left(\begin{array}{c}0.48{e}^{(0.31){\varvec{\pi}}{{\it i}}},\\ 0.46{e}^{(0.23){\varvec{\pi}}{{\it i}}},\\ 0.40{e}^{\left(0.39\right){\varvec{\pi}}{{\it i}}}\end{array}\right),\left(\begin{array}{c}0.49{e}^{\left(0.23\right){\varvec{\pi}}{{\it i}}},\\ 0.54{e}^{\left(0.39\right){\varvec{\pi}}{{\it i}}},\\ 0.40{e}^{\left(0.45\right){\varvec{\pi}}{{\it i}}}\end{array}\right),\left(\begin{array}{c}0.46{e}^{\left(0.34\right){\varvec{\pi}}{{\it i}}},\\ 0.35{e}^{\left(0.46\right){\varvec{\pi}}{{\it i}}},\\ 0.38{e}^{\left(0.40\right){\varvec{\pi}}{{\it i}}}\end{array}\right),\left(\begin{array}{c}0.49{e}^{\left(0.36\right){\varvec{\pi}}{{\it i}}},\\ 0.43{e}^{\left(0.41\right){\varvec{\pi}}{{\it i}}},\\ 0.43{e}^{\left(0.48\right){\varvec{\pi}}{{\it i}}}\end{array}\right)\right)\end{array}\right\}.$$

### Definition 20

A CSFSR $\raisebox{-4.5pt}{{\rm R}}\rotatebox{45}{\hspace*{-11pt}--}$ on $F$ is called CSFS-transitive-R if

$$\forall \left(s,{{\psi }_{{\acute{\text{\AA}}}}}_{c}\left(s\right),{{\mu }_{{\acute{\text{\AA}}}}}_{c}\left(s\right),{{\eta }_{{\acute{\text{\AA}}}}}_{c}\left(s\right)\right),\left(f,{{\psi }_{{\acute{\text{\AA}}}}}_{c}\left(f\right),{{\mu }_{{\acute{\text{\AA}}}}}_{c}\left(f\right),{{\eta }_{{\acute{\text{\AA}}}}}_{c}\left(f\right)\right)and\left(g{\hspace*{-7pt}\neg},{{\psi }_{{\acute{\text{\AA}}}}}_{c}\left(g{\hspace*{-7pt}\neg}\right),{{\mu }_{{\acute{\text{\AA}}}}}_{c}\left(g{\hspace*{-7pt}\neg}\right),{{\eta }_{{\acute{\text{\AA}}}}}_{c}\left(g{\hspace*{-7pt}\neg}\right)\right)\in F.$$

Then $$\left(\left(s,f\right),{{\psi }_{{\acute{\text{\AA}}}}}_{c}\left(s,f\right),{{\mu }_{{\acute{\text{\AA}}}}}_{c}\left(s,f\right),{{\eta }_{{\acute{\text{\AA}}}}}_{c}\left(s,f\right)\right)\in {\raisebox{-4.5pt}{{\rm R}}\rotatebox{45}{\hspace*{-11pt}--}}and\left(\left(f,g{\hspace*{-7pt}\neg}\right),{{\psi }_{{\acute{\text{\AA}}}}}_{c}\left(f,g{\hspace*{-7pt}\neg}\right),{{\mu }_{{\acute{\text{\AA}}}}}_{c}\left(f,g{\hspace*{-7pt}\neg}\right),{{\eta }_{{\acute{\text{\AA}}}}}_{c}\left(f,g{\hspace*{-7pt}\neg}\right)\right)\in \raisebox{-4.5pt}{{\rm R}}\rotatebox{45}{\hspace*{-11pt}--}$$

$$  \Rightarrow  \left(\left(s,g{\hspace*{-7pt}\neg}\right),{{\psi }_{{\acute{\text{\AA}}}}}_{c}\left(s,g{\hspace*{-7pt}\neg}\right),{{\mu }_{{\acute{\text{\AA}}}}}_{c}\left(s,g{\hspace*{-7pt}\neg}\right),{{\eta }_{{\acute{\text{\AA}}}}}_{c}\left(s,g{\hspace*{-7pt}\neg}\right)\right)\in \raisebox{-4.5pt}{{\rm R}}\rotatebox{45}{\hspace*{-11pt}--}.$$

### Example 12

Table 1 shows the CP of CSFSR. The CSFS-transitive-R is,

$$\raisebox{-4.5pt}{{\rm R}}\rotatebox{45}{\hspace*{-11pt}--}=\left\{\begin{array}{c}\left(\left({s}_{1},{s}_{2}\right),\left(\begin{array}{c}0.44{e}^{(0.29){\varvec{\pi}}{{\it i}}},\\ 0.40{e}^{(0.30){\varvec{\pi}}{{\it i}}},\\ 0.37{e}^{\left(0.40\right){\varvec{\pi}}{{\it i}}}\end{array}\right),\left(\begin{array}{c}0.48{e}^{\left(0.40\right){\varvec{\pi}}{{\it i}}},\\ 0.41{e}^{\left(0.38\right){\varvec{\pi}}{{\it i}}},\\ 0.41{e}^{\left(0.40\right){\varvec{\pi}}{{\it i}}}\end{array}\right),\left(\begin{array}{c}0.53{e}^{\left(0.41\right){\varvec{\pi}}{{\it i}}},\\ 0.52{e}^{\left(0.33\right){\varvec{\pi}}{{\it i}}},\\ 0.38{e}^{\left(0.48\right){\varvec{\pi}}{{\it i}}}\end{array}\right),\left(\begin{array}{c}0.51{e}^{\left(0.30\right){\varvec{\pi}}{{\it i}}},\\ 0.35{e}^{\left(0.29\right){\varvec{\pi}}{{\it i}}},\\ 0.45{e}^{\left(0.51\right){\varvec{\pi}}{{\it i}}}\end{array}\right)\right),\\ \left(\left({s}_{2},{s}_{3}\right),\left(\begin{array}{c}0.48{e}^{(0.31){\varvec{\pi}}{{\it i}}},\\ 0.46{e}^{(0.23){\varvec{\pi}}{{\it i}}},\\ 0.40{e}^{\left(0.39\right){\varvec{\pi}}{{\it i}}}\end{array}\right),\left(\begin{array}{c}0.49{e}^{\left(0.23\right){\varvec{\pi}}{{\it i}}},\\ 0.54{e}^{\left(0.39\right){\varvec{\pi}}{{\it i}}},\\ 0.40{e}^{\left(0.45\right){\varvec{\pi}}{{\it i}}}\end{array}\right),\left(\begin{array}{c}0.46{e}^{\left(0.34\right){\varvec{\pi}}{{\it i}}},\\ 0.35{e}^{\left(0.46\right){\varvec{\pi}}{{\it i}}},\\ 0.38{e}^{\left(0.40\right){\varvec{\pi}}{{\it i}}}\end{array}\right),\left(\begin{array}{c}0.49{e}^{\left(0.36\right){\varvec{\pi}}{{\it i}}},\\ 0.43{e}^{\left(0.41\right){\varvec{\pi}}{{\it i}}},\\ 0.43{e}^{\left(0.48\right){\varvec{\pi}}{{\it i}}}\end{array}\right)\right),\\ \left(\left({s}_{1},{s}_{3}\right),\left(\begin{array}{c}0.44{e}^{(0.29){\varvec{\pi}}{{\it i}}},\\ 0.40{e}^{(0.30){\varvec{\pi}}{{\it i}}},\\ 0.40{e}^{\left(0.39\right){\varvec{\pi}}{{\it i}}}\end{array}\right),\left(\begin{array}{c}0.48{e}^{\left(0.39\right){\varvec{\pi}}{{\it i}}},\\ 0.41{e}^{\left(0.40\right){\varvec{\pi}}{{\it i}}},\\ 0.40{e}^{\left(0.45\right){\varvec{\pi}}{{\it i}}}\end{array}\right),\left(\begin{array}{c}0.53{e}^{\left(0.45\right){\varvec{\pi}}{{\it i}}},\\ 0.41{e}^{\left(0.33\right){\varvec{\pi}}{{\it i}}},\\ 0.38{e}^{\left(0.48\right){\varvec{\pi}}{{\it i}}}\end{array}\right),\left(\begin{array}{c}0.51{e}^{\left(0.30\right){\varvec{\pi}}{{\it i}}},\\ 0.49{e}^{\left(0.29\right){\varvec{\pi}}{{\it i}}},\\ 0.43{e}^{\left(0.46\right){\varvec{\pi}}{{\it i}}}\end{array}\right)\right)\end{array}\right\}.$$

### Definition 21

A CSFSR $\raisebox{-4.5pt}{{\rm R}}\rotatebox{45}{\hspace*{-11pt}--}$ on $F$ is called CSFS-equivalence-R if it is

- Reflexive;
- Symmetric;
- Transitive.

### Example 13

Table 1 shows the CP of CSFSR. The CSFS-equivalence-R is,

$$\raisebox{-4.5pt}{{\rm R}}\rotatebox{45}{\hspace*{-11pt}--}=\left\{\begin{array}{c}\left(\left({s}_{2},{s}_{3}\right),\left(\begin{array}{c}0.48{e}^{(0.31){\varvec{\pi}}{{\it i}}},\\ 0.46{e}^{(0.23){\varvec{\pi}}{{\it i}}},\\ 0.40{e}^{\left(0.39\right){\varvec{\pi}}{{\it i}}}\end{array}\right),\left(\begin{array}{c}0.49{e}^{\left(0.23\right){\varvec{\pi}}{{\it i}}},\\ 0.54{e}^{\left(0.39\right){\varvec{\pi}}{{\it i}}},\\ 0.40{e}^{\left(0.45\right){\varvec{\pi}}{{\it i}}}\end{array}\right),\left(\begin{array}{c}0.46{e}^{\left(0.34\right){\varvec{\pi}}{{\it i}}},\\ 0.35{e}^{\left(0.46\right){\varvec{\pi}}{{\it i}}},\\ 0.38{e}^{\left(0.40\right){\varvec{\pi}}{{\it i}}}\end{array}\right),\left(\begin{array}{c}0.49{e}^{\left(0.36\right){\varvec{\pi}}{{\it i}}},\\ 0.43{e}^{\left(0.41\right){\varvec{\pi}}{{\it i}}},\\ 0.43{e}^{\left(0.48\right){\varvec{\pi}}{{\it i}}}\end{array}\right)\right),\\ \left(\left({s}_{1},{s}_{1}\right),\left(\begin{array}{c}0.44{e}^{\left(0.29\right){\varvec{\pi}}{{\it i}}},\\ 0.35{e}^{\left(0.30\right){\varvec{\pi}}{{\it i}}},\\ 0.34{e}^{\left(0.40\right){\varvec{\pi}}{{\it i}}}\end{array}\right),\left(\begin{array}{c}0.48{e}^{\left(0.41\right){\varvec{\pi}}{{\it i}}},\\ 0.41{e}^{\left(0.40\right){\varvec{\pi}}{{\it i}}},\\ 0.39{e}^{\left(0.45\right){\varvec{\pi}}{{\it i}}}\end{array}\right),\left(\begin{array}{c}0.52{e}^{\left(0.41\right){\varvec{\pi}}{{\it i}}},\\ 0.53{e}^{\left(0.33\right){\varvec{\pi}}{{\it i}}},\\ 0.51{e}^{\left(0.48\right){\varvec{\pi}}{{\it i}}}\end{array}\right),\left(\begin{array}{c}0.51{e}^{\left(0.30\right){\varvec{\pi}}{{\it i}}},\\ 0.35{e}^{\left(0.29\right){\varvec{\pi}}{{\it i}}},\\ 0.43{e}^{\left(0.49\right){\varvec{\pi}}{{\it i}}}\end{array}\right)\right),\\ \left(\left({s}_{3},{s}_{2}\right),\left(\begin{array}{c}0.55{e}^{(0.34){\varvec{\pi}}{{\it i}}},\\ 0.45{e}^{(0.39){\varvec{\pi}}{{\it i}}},\\ 0.37{e}^{\left(0.40\right){\varvec{\pi}}{{\it i}}}\end{array}\right),\left(\begin{array}{c}0.51{e}^{\left(0.40\right){\varvec{\pi}}{{\it i}}},\\ 0.49{e}^{\left(0.33\right){\varvec{\pi}}{{\it i}}},\\ 0.41{e}^{\left(0.40\right){\varvec{\pi}}{{\it i}}}\end{array}\right),\left(\begin{array}{c}0.53{e}^{\left(0.41\right){\varvec{\pi}}{{\it i}}},\\ 0.42{e}^{\left(0.39\right){\varvec{\pi}}{{\it i}}},\\ 0.38{e}^{\left(0.39\right){\varvec{\pi}}{{\it i}}}\end{array}\right),\left(\begin{array}{c}0.54{e}^{\left(0.41\right){\varvec{\pi}}{{\it i}}},\\ 0.35{e}^{\left(0.41\right){\varvec{\pi}}{{\it i}}},\\ 0.45{e}^{\left(0.51\right){\varvec{\pi}}{{\it i}}}\end{array}\right)\right),\\ \left(\left({s}_{2,}{s}_{2}\right),\left(\begin{array}{c}0.48{e}^{\left(0.31\right){\varvec{\pi}}{{\it i}}},\\ 0.49{e}^{\left(0.23\right){\varvec{\pi}}{{\it i}}},\\ 0.37{e}^{\left(0.40\right){\varvec{\pi}}{{\it i}}}\end{array}\right),\left(\begin{array}{c}0.49{e}^{\left(0.23\right){\varvec{\pi}}{{\it i}}},\\ 0.49{e}^{\left(0.38\right){\varvec{\pi}}{{\it i}}},\\ 0.41{e}^{\left(0.40\right){\varvec{\pi}}{{\it i}}}\end{array}\right),\left(\begin{array}{c}0.46{e}^{\left(0.34\right){\varvec{\pi}}{{\it i}}},\\ 0.35{e}^{\left(0.39\right){\varvec{\pi}}{{\it i}}},\\ 0.38{e}^{\left(0.39\right){\varvec{\pi}}{{\it i}}}\end{array}\right),\left(\begin{array}{c}0.49{e}^{\left(0.36\right){\varvec{\pi}}{{\it i}}},\\ 0.35{e}^{\left(0.47\right){\varvec{\pi}}{{\it i}}},\\ 0.45{e}^{\left(0.51\right){\varvec{\pi}}{{\it i}}}\end{array}\right)\right),\\ \left(\left({s}_{3},{s}_{3}\right),\left(\begin{array}{c}0.55{e}^{(0.34){\varvec{\pi}}{{\it i}}},\\ 0.45{e}^{(0.39){\varvec{\pi}}{{\it i}}},\\ 0.40{e}^{\left(0.40\right){\varvec{\pi}}{{\it i}}}\end{array}\right),\left(\begin{array}{c}0.52{e}^{\left(0.39\right){\varvec{\pi}}{{\it i}}},\\ 0.39{e}^{\left(0.33\right){\varvec{\pi}}{{\it i}}},\\ 0.41{e}^{\left(0.45\right){\varvec{\pi}}{{\it i}}}\end{array}\right),\left(\begin{array}{c}0.53{e}^{\left(0.45\right){\varvec{\pi}}{{\it i}}},\\ 0.41{e}^{\left(0.46\right){\varvec{\pi}}{{\it i}}},\\ 0.39{e}^{\left(0.40\right){\varvec{\pi}}{{\it i}}}\end{array}\right),\left(\begin{array}{c}0.54{e}^{\left(0.46\right){\varvec{\pi}}{{\it i}}},\\ 0.51{e}^{\left(0.41\right){\varvec{\pi}}{{\it i}}},\\ 0.43{e}^{\left(0.46\right){\varvec{\pi}}{{\it i}}}\end{array}\right)\right)\end{array}\right\}.$$

### Definition 22

A CSFSR $\raisebox{-4.5pt}{{\rm R}}\rotatebox{45}{\hspace*{-11pt}--}$ on $F$ is called CSFS-partial order-R if it is

- Reflexive
- Anti-Symmetric
- Transitive

### Example 14

Table 1 shows the CP of CSFSR. The CSFS-partial order-R is,

$$\raisebox{-4.5pt}{{\rm R}}\rotatebox{45}{\hspace*{-11pt}--}=\left\{\begin{array}{c}\left(\left({s}_{1},{s}_{2}\right),\left(\begin{array}{c}0.44{e}^{(0.29){\varvec{\pi}}{{\it i}}},\\ 0.40{e}^{(0.30){\varvec{\pi}}{{\it i}}},\\ 0.37{e}^{\left(0.40\right){\varvec{\pi}}{{\it i}}}\end{array}\right),\left(\begin{array}{c}0.48{e}^{\left(0.40\right){\varvec{\pi}}{{\it i}}},\\ 0.41{e}^{\left(0.38\right){\varvec{\pi}}{{\it i}}},\\ 0.41{e}^{\left(0.40\right){\varvec{\pi}}{{\it i}}}\end{array}\right),\left(\begin{array}{c}0.53{e}^{\left(0.41\right){\varvec{\pi}}{{\it i}}},\\ 0.52{e}^{\left(0.33\right){\varvec{\pi}}{{\it i}}},\\ 0.38{e}^{\left(0.48\right){\varvec{\pi}}{{\it i}}}\end{array}\right),\left(\begin{array}{c}0.51{e}^{\left(0.30\right){\varvec{\pi}}{{\it i}}},\\ 0.35{e}^{\left(0.29\right){\varvec{\pi}}{{\it i}}},\\ 0.45{e}^{\left(0.51\right){\varvec{\pi}}{{\it i}}}\end{array}\right)\right),\\ \left(\left({s}_{1},{s}_{1}\right),\left(\begin{array}{c}0.44{e}^{\left(0.29\right){\varvec{\pi}}{{\it i}}},\\ 0.35{e}^{\left(0.30\right){\varvec{\pi}}{{\it i}}},\\ 0.34{e}^{\left(0.40\right){\varvec{\pi}}{{\it i}}}\end{array}\right),\left(\begin{array}{c}0.48{e}^{\left(0.41\right){\varvec{\pi}}{{\it i}}},\\ 0.41{e}^{\left(0.40\right){\varvec{\pi}}{{\it i}}},\\ 0.39{e}^{\left(0.45\right){\varvec{\pi}}{{\it i}}}\end{array}\right),\left(\begin{array}{c}0.52{e}^{\left(0.41\right){\varvec{\pi}}{{\it i}}},\\ 0.53{e}^{\left(0.33\right){\varvec{\pi}}{{\it i}}},\\ 0.51{e}^{\left(0.48\right){\varvec{\pi}}{{\it i}}}\end{array}\right),\left(\begin{array}{c}0.51{e}^{\left(0.30\right){\varvec{\pi}}{{\it i}}},\\ 0.35{e}^{\left(0.29\right){\varvec{\pi}}{{\it i}}},\\ 0.43{e}^{\left(0.49\right){\varvec{\pi}}{{\it i}}}\end{array}\right)\right),\\ \left(\left({s}_{2,}{s}_{2}\right),\left(\begin{array}{c}0.48{e}^{\left(0.31\right){\varvec{\pi}}{{\it i}}},\\ 0.49{e}^{\left(0.23\right){\varvec{\pi}}{{\it i}}},\\ 0.37{e}^{\left(0.40\right){\varvec{\pi}}{{\it i}}}\end{array}\right),\left(\begin{array}{c}0.49{e}^{\left(0.23\right){\varvec{\pi}}{{\it i}}},\\ 0.49{e}^{\left(0.38\right){\varvec{\pi}}{{\it i}}},\\ 0.41{e}^{\left(0.40\right){\varvec{\pi}}{{\it i}}}\end{array}\right),\left(\begin{array}{c}0.46{e}^{\left(0.34\right){\varvec{\pi}}{{\it i}}},\\ 0.35{e}^{\left(0.39\right){\varvec{\pi}}{{\it i}}},\\ 0.38{e}^{\left(0.39\right){\varvec{\pi}}{{\it i}}}\end{array}\right),\left(\begin{array}{c}0.49{e}^{\left(0.36\right){\varvec{\pi}}{{\it i}}},\\ 0.35{e}^{\left(0.47\right){\varvec{\pi}}{{\it i}}},\\ 0.45{e}^{\left(0.51\right){\varvec{\pi}}{{\it i}}}\end{array}\right)\right),\\ \left(\left({s}_{1},{s}_{1}\right),\left(\begin{array}{c}0.44{e}^{\left(0.29\right){\varvec{\pi}}{{\it i}}},\\ 0.35{e}^{\left(0.30\right){\varvec{\pi}}{{\it i}}},\\ 0.34{e}^{\left(0.40\right){\varvec{\pi}}{{\it i}}}\end{array}\right),\left(\begin{array}{c}0.48{e}^{\left(0.41\right){\varvec{\pi}}{{\it i}}},\\ 0.41{e}^{\left(0.40\right){\varvec{\pi}}{{\it i}}},\\ 0.39{e}^{\left(0.45\right){\varvec{\pi}}{{\it i}}}\end{array}\right),\left(\begin{array}{c}0.52{e}^{\left(0.41\right){\varvec{\pi}}{{\it i}}},\\ 0.53{e}^{\left(0.33\right){\varvec{\pi}}{{\it i}}},\\ 0.51{e}^{\left(0.48\right){\varvec{\pi}}{{\it i}}}\end{array}\right),\left(\begin{array}{c}0.51{e}^{\left(0.30\right){\varvec{\pi}}{{\it i}}},\\ 0.35{e}^{\left(0.29\right){\varvec{\pi}}{{\it i}}},\\ 0.43{e}^{\left(0.49\right){\varvec{\pi}}{{\it i}}}\end{array}\right)\right),\\ \left(\left({s}_{3},{s}_{3}\right),\left(\begin{array}{c}0.55{e}^{(0.34){\varvec{\pi}}{{\it i}}},\\ 0.45{e}^{(0.39){\varvec{\pi}}{{\it i}}},\\ 0.40{e}^{\left(0.40\right){\varvec{\pi}}{{\it i}}}\end{array}\right),\left(\begin{array}{c}0.52{e}^{\left(0.39\right){\varvec{\pi}}{{\it i}}},\\ 0.39{e}^{\left(0.33\right){\varvec{\pi}}{{\it i}}},\\ 0.41{e}^{\left(0.45\right){\varvec{\pi}}{{\it i}}}\end{array}\right),\left(\begin{array}{c}0.53{e}^{\left(0.45\right){\varvec{\pi}}{{\it i}}},\\ 0.41{e}^{\left(0.46\right){\varvec{\pi}}{{\it i}}},\\ 0.39{e}^{\left(0.40\right){\varvec{\pi}}{{\it i}}}\end{array}\right),\left(\begin{array}{c}0.54{e}^{\left(0.46\right){\varvec{\pi}}{{\it i}}},\\ 0.51{e}^{\left(0.41\right){\varvec{\pi}}{{\it i}}},\\ 0.43{e}^{\left(0.46\right){\varvec{\pi}}{{\it i}}}\end{array}\right)\right),\\ \left(\left({s}_{2},{s}_{3}\right),\left(\begin{array}{c}0.48{e}^{(0.31){\varvec{\pi}}{{\it i}}},\\ 0.46{e}^{(0.23){\varvec{\pi}}{{\it i}}},\\ 0.40{e}^{\left(0.39\right){\varvec{\pi}}{{\it i}}}\end{array}\right),\left(\begin{array}{c}0.49{e}^{\left(0.23\right){\varvec{\pi}}{{\it i}}},\\ 0.54{e}^{\left(0.39\right){\varvec{\pi}}{{\it i}}},\\ 0.40{e}^{\left(0.45\right){\varvec{\pi}}{{\it i}}}\end{array}\right),\left(\begin{array}{c}0.46{e}^{\left(0.34\right){\varvec{\pi}}{{\it i}}},\\ 0.35{e}^{\left(0.46\right){\varvec{\pi}}{{\it i}}},\\ 0.38{e}^{\left(0.40\right){\varvec{\pi}}{{\it i}}}\end{array}\right),\left(\begin{array}{c}0.49{e}^{\left(0.36\right){\varvec{\pi}}{{\it i}}},\\ 0.43{e}^{\left(0.41\right){\varvec{\pi}}{{\it i}}},\\ 0.43{e}^{\left(0.48\right){\varvec{\pi}}{{\it i}}}\end{array}\right)\right)\end{array}\right\}.$$

### Definition 23

A CSFSR $\raisebox{-4.5pt}{{\rm R}}\rotatebox{45}{\hspace*{-11pt}--}$ on $F$ is called CSFS-pre order-R if it is

- Reflexive;
- Transitive.

### Example 15

Table 1 shows the CP of CSFSR. The CSFS-pre order-R is,

$$\raisebox{-4.5pt}{{\rm R}}\rotatebox{45}{\hspace*{-11pt}--}=\left\{\begin{array}{c}\left(\left({s}_{1},{s}_{1}\right),\left(\begin{array}{c}0.44{e}^{\left(0.29\right){\varvec{\pi}}{{\it i}}},\\ 0.35{e}^{\left(0.30\right){\varvec{\pi}}{{\it i}}},\\ 0.34{e}^{\left(0.40\right){\varvec{\pi}}{{\it i}}}\end{array}\right),\left(\begin{array}{c}0.48{e}^{\left(0.41\right){\varvec{\pi}}{{\it i}}},\\ 0.41{e}^{\left(0.40\right){\varvec{\pi}}{{\it i}}},\\ 0.39{e}^{\left(0.45\right){\varvec{\pi}}{{\it i}}}\end{array}\right),\left(\begin{array}{c}0.52{e}^{\left(0.41\right){\varvec{\pi}}{{\it i}}},\\ 0.53{e}^{\left(0.33\right){\varvec{\pi}}{{\it i}}},\\ 0.51{e}^{\left(0.48\right){\varvec{\pi}}{{\it i}}}\end{array}\right),\left(\begin{array}{c}0.51{e}^{\left(0.30\right){\varvec{\pi}}{{\it i}}},\\ 0.35{e}^{\left(0.29\right){\varvec{\pi}}{{\it i}}},\\ 0.43{e}^{\left(0.49\right){\varvec{\pi}}{{\it i}}}\end{array}\right)\right),\\ \left(\left({s}_{1},{s}_{3}\right),\left(\begin{array}{c}0.44{e}^{(0.29){\varvec{\pi}}{{\it i}}},\\ 0.40{e}^{(0.30){\varvec{\pi}}{{\it i}}},\\ 0.40{e}^{\left(0.39\right){\varvec{\pi}}{{\it i}}}\end{array}\right),\left(\begin{array}{c}0.48{e}^{\left(0.39\right){\varvec{\pi}}{{\it i}}},\\ 0.41{e}^{\left(0.40\right){\varvec{\pi}}{{\it i}}},\\ 0.40{e}^{\left(0.45\right){\varvec{\pi}}{{\it i}}}\end{array}\right),\left(\begin{array}{c}0.53{e}^{\left(0.45\right){\varvec{\pi}}{{\it i}}},\\ 0.41{e}^{\left(0.33\right){\varvec{\pi}}{{\it i}}},\\ 0.38{e}^{\left(0.48\right){\varvec{\pi}}{{\it i}}}\end{array}\right),\left(\begin{array}{c}0.51{e}^{\left(0.30\right){\varvec{\pi}}{{\it i}}},\\ 0.49{e}^{\left(0.29\right){\varvec{\pi}}{{\it i}}},\\ 0.43{e}^{\left(0.46\right){\varvec{\pi}}{{\it i}}}\end{array}\right)\right),\\ \left(\left({s}_{1},{s}_{2}\right),\left(\begin{array}{c}0.44{e}^{(0.29){\varvec{\pi}}{{\it i}}},\\ 0.40{e}^{(0.30){\varvec{\pi}}{{\it i}}},\\ 0.37{e}^{\left(0.40\right){\varvec{\pi}}{{\it i}}}\end{array}\right),\left(\begin{array}{c}0.48{e}^{\left(0.40\right){\varvec{\pi}}{{\it i}}},\\ 0.41{e}^{\left(0.38\right){\varvec{\pi}}{{\it i}}},\\ 0.41{e}^{\left(0.40\right){\varvec{\pi}}{{\it i}}}\end{array}\right),\left(\begin{array}{c}0.53{e}^{\left(0.41\right){\varvec{\pi}}{{\it i}}},\\ 0.52{e}^{\left(0.33\right){\varvec{\pi}}{{\it i}}},\\ 0.38{e}^{\left(0.48\right){\varvec{\pi}}{{\it i}}}\end{array}\right),\left(\begin{array}{c}0.51{e}^{\left(0.30\right){\varvec{\pi}}{{\it i}}},\\ 0.35{e}^{\left(0.29\right){\varvec{\pi}}{{\it i}}},\\ 0.45{e}^{\left(0.51\right){\varvec{\pi}}{{\it i}}}\end{array}\right)\right),\\ \left(\left({s}_{2},{s}_{3}\right),\left(\begin{array}{c}0.48{e}^{(0.31){\varvec{\pi}}{{\it i}}},\\ 0.46{e}^{(0.23){\varvec{\pi}}{{\it i}}},\\ 0.40{e}^{\left(0.39\right){\varvec{\pi}}{{\it i}}}\end{array}\right),\left(\begin{array}{c}0.49{e}^{\left(0.23\right){\varvec{\pi}}{{\it i}}},\\ 0.54{e}^{\left(0.39\right){\varvec{\pi}}{{\it i}}},\\ 0.40{e}^{\left(0.45\right){\varvec{\pi}}{{\it i}}}\end{array}\right),\left(\begin{array}{c}0.46{e}^{\left(0.34\right){\varvec{\pi}}{{\it i}}},\\ 0.35{e}^{\left(0.46\right){\varvec{\pi}}{{\it i}}},\\ 0.38{e}^{\left(0.40\right){\varvec{\pi}}{{\it i}}}\end{array}\right),\left(\begin{array}{c}0.49{e}^{\left(0.36\right){\varvec{\pi}}{{\it i}}},\\ 0.43{e}^{\left(0.41\right){\varvec{\pi}}{{\it i}}},\\ 0.43{e}^{\left(0.48\right){\varvec{\pi}}{{\it i}}}\end{array}\right)\right),\\ \left(\left({s}_{2,}{s}_{2}\right),\left(\begin{array}{c}0.48{e}^{\left(0.31\right){\varvec{\pi}}{{\it i}}},\\ 0.49{e}^{\left(0.23\right){\varvec{\pi}}{{\it i}}},\\ 0.37{e}^{\left(0.40\right){\varvec{\pi}}{{\it i}}}\end{array}\right),\left(\begin{array}{c}0.49{e}^{\left(0.23\right){\varvec{\pi}}{{\it i}}},\\ 0.49{e}^{\left(0.38\right){\varvec{\pi}}{{\it i}}},\\ 0.41{e}^{\left(0.40\right){\varvec{\pi}}{{\it i}}}\end{array}\right),\left(\begin{array}{c}0.46{e}^{\left(0.34\right){\varvec{\pi}}{{\it i}}},\\ 0.35{e}^{\left(0.39\right){\varvec{\pi}}{{\it i}}},\\ 0.38{e}^{\left(0.39\right){\varvec{\pi}}{{\it i}}}\end{array}\right),\left(\begin{array}{c}0.49{e}^{\left(0.36\right){\varvec{\pi}}{{\it i}}},\\ 0.35{e}^{\left(0.47\right){\varvec{\pi}}{{\it i}}},\\ 0.45{e}^{\left(0.51\right){\varvec{\pi}}{{\it i}}}\end{array}\right)\right),\\ \left(\left({s}_{3},{s}_{3}\right),\left(\begin{array}{c}0.55{e}^{(0.34){\varvec{\pi}}{{\it i}}},\\ 0.45{e}^{(0.39){\varvec{\pi}}{{\it i}}},\\ 0.40{e}^{\left(0.40\right){\varvec{\pi}}{{\it i}}}\end{array}\right),\left(\begin{array}{c}0.52{e}^{\left(0.39\right){\varvec{\pi}}{{\it i}}},\\ 0.39{e}^{\left(0.33\right){\varvec{\pi}}{{\it i}}},\\ 0.41{e}^{\left(0.45\right){\varvec{\pi}}{{\it i}}}\end{array}\right),\left(\begin{array}{c}0.53{e}^{\left(0.45\right){\varvec{\pi}}{{\it i}}},\\ 0.41{e}^{\left(0.46\right){\varvec{\pi}}{{\it i}}},\\ 0.39{e}^{\left(0.40\right){\varvec{\pi}}{{\it i}}}\end{array}\right),\left(\begin{array}{c}0.54{e}^{\left(0.46\right){\varvec{\pi}}{{\it i}}},\\ 0.51{e}^{\left(0.41\right){\varvec{\pi}}{{\it i}}},\\ 0.43{e}^{\left(0.46\right){\varvec{\pi}}{{\it i}}}\end{array}\right)\right)\end{array}\right\}.$$

### Definition 24

A CSFSR $\raisebox{-4.5pt}{{\rm R}}\rotatebox{45}{\hspace*{-11pt}--}$ on $F$ is called CSFS-linear order-R if it is

- Reflexive;
- Anti-symmetric;
- Transitive;
- Complete.

### Example 16

Table 1 shows the CP of CSFSR. The CSFS-linear order-R is,

$${\raisebox{-4.5pt}{{\rm R}}\rotatebox{45}{\hspace*{-11pt}--}}=\left\{\begin{array}{c}\left(\left({s}_{2},{s}_{1}\right),\left(\begin{array}{c}0.48{e}^{\left(0.31\right){\varvec{\pi}}{{\it i}}},\\ 0.35{e}^{\left(0.23\right){\varvec{\pi}}{{\it i}}},\\ 0.34{e}^{\left(0.40\right){\varvec{\pi}}{{\it i}}}\end{array}\right),\left(\begin{array}{c}0.49{e}^{\left(0.23\right){\varvec{\pi}}{{\it i}}},\\ 0.47{e}^{\left(0.39\right){\varvec{\pi}}{{\it i}}},\\ 0.39{e}^{\left(0.45\right){\varvec{\pi}}{{\it i}}}\end{array}\right),\left(\begin{array}{c}0.46{e}^{\left(0.34\right){\varvec{\pi}}{{\it i}}},\\ 0.35{e}^{\left(0.40\right){\varvec{\pi}}{{\it i}}},\\ 0.51{e}^{\left(0.39\right){\varvec{\pi}}{{\it i}}}\end{array}\right),\left(\begin{array}{c}0.49{e}^{\left(0.36\right){\varvec{\pi}}{{\it i}}},\\ 0.35{e}^{\left(0.35\right){\varvec{\pi}}{{\it i}}},\\ 0.43{e}^{\left(0.49\right){\varvec{\pi}}{{\it i}}}\end{array}\right)\right),\\ \left(\left({s}_{1},{s}_{1}\right),\left(\begin{array}{c}0.44{e}^{\left(0.29\right){\varvec{\pi}}{{\it i}}},\\ 0.35{e}^{\left(0.30\right){\varvec{\pi}}{{\it i}}},\\ 0.34{e}^{\left(0.40\right){\varvec{\pi}}{{\it i}}}\end{array}\right),\left(\begin{array}{c}0.48{e}^{\left(0.41\right){\varvec{\pi}}{{\it i}}},\\ 0.41{e}^{\left(0.40\right){\varvec{\pi}}{{\it i}}},\\ 0.39{e}^{\left(0.45\right){\varvec{\pi}}{{\it i}}}\end{array}\right),\left(\begin{array}{c}0.52{e}^{\left(0.41\right){\varvec{\pi}}{{\it i}}},\\ 0.53{e}^{\left(0.33\right){\varvec{\pi}}{{\it i}}},\\ 0.51{e}^{\left(0.48\right){\varvec{\pi}}{{\it i}}}\end{array}\right),\left(\begin{array}{c}0.51{e}^{\left(0.30\right){\varvec{\pi}}{{\it i}}},\\ 0.35{e}^{\left(0.29\right){\varvec{\pi}}{{\it i}}},\\ 0.43{e}^{\left(0.49\right){\varvec{\pi}}{{\it i}}}\end{array}\right)\right),\\ \left(\left({s}_{2,}{s}_{2}\right),\left(\begin{array}{c}0.48{e}^{\left(0.31\right){\varvec{\pi}}{{\it i}}},\\ 0.49{e}^{\left(0.23\right){\varvec{\pi}}{{\it i}}},\\ 0.37{e}^{\left(0.40\right){\varvec{\pi}}{{\it i}}}\end{array}\right),\left(\begin{array}{c}0.49{e}^{\left(0.23\right){\varvec{\pi}}{{\it i}}},\\ 0.49{e}^{\left(0.38\right){\varvec{\pi}}{{\it i}}},\\ 0.41{e}^{\left(0.40\right){\varvec{\pi}}{{\it i}}}\end{array}\right),\left(\begin{array}{c}0.46{e}^{\left(0.34\right){\varvec{\pi}}{{\it i}}},\\ 0.35{e}^{\left(0.39\right){\varvec{\pi}}{{\it i}}},\\ 0.38{e}^{\left(0.39\right){\varvec{\pi}}{{\it i}}}\end{array}\right),\left(\begin{array}{c}0.49{e}^{\left(0.36\right){\varvec{\pi}}{{\it i}}},\\ 0.35{e}^{\left(0.47\right){\varvec{\pi}}{{\it i}}},\\ 0.45{e}^{\left(0.51\right){\varvec{\pi}}{{\it i}}}\end{array}\right)\right),\\ \left(\left({s}_{3},{s}_{3}\right),\left(\begin{array}{c}0.55{e}^{(0.34){\varvec{\pi}}{{\it i}}},\\ 0.45{e}^{(0.39){\varvec{\pi}}{{\it i}}},\\ 0.40{e}^{\left(0.40\right){\varvec{\pi}}{{\it i}}}\end{array}\right),\left(\begin{array}{c}0.52{e}^{\left(0.39\right){\varvec{\pi}}{{\it i}}},\\ 0.39{e}^{\left(0.33\right){\varvec{\pi}}{{\it i}}},\\ 0.41{e}^{\left(0.45\right){\varvec{\pi}}{{\it i}}}\end{array}\right),\left(\begin{array}{c}0.53{e}^{\left(0.45\right){\varvec{\pi}}{{\it i}}},\\ 0.41{e}^{\left(0.46\right){\varvec{\pi}}{{\it i}}},\\ 0.39{e}^{\left(0.40\right){\varvec{\pi}}{{\it i}}}\end{array}\right),\left(\begin{array}{c}0.54{e}^{\left(0.46\right){\varvec{\pi}}{{\it i}}},\\ 0.51{e}^{\left(0.41\right){\varvec{\pi}}{{\it i}}},\\ 0.43{e}^{\left(0.46\right){\varvec{\pi}}{{\it i}}}\end{array}\right)\right),\\ \left(\left({s}_{3},{s}_{2}\right),\left(\begin{array}{c}0.55{e}^{(0.34){\varvec{\pi}}{{\it i}}},\\ 0.45{e}^{(0.39){\varvec{\pi}}{{\it i}}},\\ 0.37{e}^{\left(0.40\right){\varvec{\pi}}{{\it i}}}\end{array}\right),\left(\begin{array}{c}0.51{e}^{\left(0.40\right){\varvec{\pi}}{{\it i}}},\\ 0.49{e}^{\left(0.33\right){\varvec{\pi}}{{\it i}}},\\ 0.41{e}^{\left(0.40\right){\varvec{\pi}}{{\it i}}}\end{array}\right),\left(\begin{array}{c}0.53{e}^{\left(0.41\right){\varvec{\pi}}{{\it i}}},\\ 0.42{e}^{\left(0.39\right){\varvec{\pi}}{{\it i}}},\\ 0.38{e}^{\left(0.39\right){\varvec{\pi}}{{\it i}}}\end{array}\right),\left(\begin{array}{c}0.54{e}^{\left(0.41\right){\varvec{\pi}}{{\it i}}},\\ 0.35{e}^{\left(0.41\right){\varvec{\pi}}{{\it i}}},\\ 0.45{e}^{\left(0.51\right){\varvec{\pi}}{{\it i}}}\end{array}\right)\right),\\ \left(\left({s}_{3},{s}_{1}\right),\left(\begin{array}{c}0.49{e}^{\left(0.34\right){\varvec{\pi}}{{\it i}}},\\ 0.35{e}^{\left(0.39\right){\varvec{\pi}}{{\it i}}},\\ 0.35{e}^{\left(0.40\right){\varvec{\pi}}{{\it i}}}\end{array}\right),\left(\begin{array}{c}0.58{e}^{\left(0.41\right){\varvec{\pi}}{{\it i}}},\\ 0.47{e}^{\left(0.33\right){\varvec{\pi}}{{\it i}}},\\ 0.41{e}^{\left(0.45\right){\varvec{\pi}}{{\it i}}}\end{array}\right),\left(\begin{array}{c}0.52{e}^{\left(0.41\right){\varvec{\pi}}{{\it i}}},\\ 0.42{e}^{\left(0.40\right){\varvec{\pi}}{{\it i}}},\\ 0.51{e}^{\left(0.40\right){\varvec{\pi}}{{\it i}}}\end{array}\right),\left(\begin{array}{c}0.54{e}^{\left(0.40\right){\varvec{\pi}}{{\it i}}},\\ 0.35{e}^{\left(0.35\right){\varvec{\pi}}{{\it i}}},\\ 0.43{e}^{\left(0.49\right){\varvec{\pi}}{{\it i}}}\end{array}\right)\right)\end{array}\right\}.$$

### Definition 25

A CSFSR $\raisebox{-4.5pt}{{\rm R}}\rotatebox{45}{\hspace*{-11pt}--}$ on $F$ is called CSFS-strict order-R if it is

- ir-reflexive;
- transitive.

### Example 17

Table 1 shows the CP of CSFSR. The CSFS-strict order-R is,

$${\raisebox{-4.5pt}{{\rm R}}\rotatebox{45}{\hspace*{-11pt}--}}=\left\{\begin{array}{c}\left(\left({s}_{1},{s}_{2}\right),\left(\begin{array}{c}0.44{e}^{(0.29){\varvec{\pi}}{{\it i}}},\\ 0.40{e}^{(0.30){\varvec{\pi}}{{\it i}}},\\ 0.37{e}^{\left(0.40\right){\varvec{\pi}}{{\it i}}}\end{array}\right),\left(\begin{array}{c}0.48{e}^{\left(0.40\right){\varvec{\pi}}{{\it i}}},\\ 0.41{e}^{\left(0.38\right){\varvec{\pi}}{{\it i}}},\\ 0.41{e}^{\left(0.40\right){\varvec{\pi}}{{\it i}}}\end{array}\right),\left(\begin{array}{c}0.53{e}^{\left(0.41\right){\varvec{\pi}}{{\it i}}},\\ 0.52{e}^{\left(0.33\right){\varvec{\pi}}{{\it i}}},\\ 0.38{e}^{\left(0.48\right){\varvec{\pi}}{{\it i}}}\end{array}\right),\left(\begin{array}{c}0.51{e}^{\left(0.30\right){\varvec{\pi}}{{\it i}}},\\ 0.35{e}^{\left(0.29\right){\varvec{\pi}}{{\it i}}},\\ 0.45{e}^{\left(0.51\right){\varvec{\pi}}{{\it i}}}\end{array}\right)\right),\\ \left(\left({s}_{2},{s}_{3}\right),\left(\begin{array}{c}0.48{e}^{(0.31){\varvec{\pi}}{{\it i}}},\\ 0.46{e}^{(0.23){\varvec{\pi}}{{\it i}}},\\ 0.40{e}^{\left(0.39\right){\varvec{\pi}}{{\it i}}}\end{array}\right),\left(\begin{array}{c}0.49{e}^{\left(0.23\right){\varvec{\pi}}{{\it i}}},\\ 0.54{e}^{\left(0.39\right){\varvec{\pi}}{{\it i}}},\\ 0.40{e}^{\left(0.45\right){\varvec{\pi}}{{\it i}}}\end{array}\right),\left(\begin{array}{c}0.46{e}^{\left(0.34\right){\varvec{\pi}}{{\it i}}},\\ 0.35{e}^{\left(0.46\right){\varvec{\pi}}{{\it i}}},\\ 0.38{e}^{\left(0.40\right){\varvec{\pi}}{{\it i}}}\end{array}\right),\left(\begin{array}{c}0.49{e}^{\left(0.36\right){\varvec{\pi}}{{\it i}}},\\ 0.43{e}^{\left(0.41\right){\varvec{\pi}}{{\it i}}},\\ 0.43{e}^{\left(0.48\right){\varvec{\pi}}{{\it i}}}\end{array}\right)\right),\\ \left(\left({s}_{1},{s}_{3}\right),\left(\begin{array}{c}0.44{e}^{(0.29){\varvec{\pi}}{{\it i}}},\\ 0.40{e}^{(0.30){\varvec{\pi}}{{\it i}}},\\ 0.40{e}^{\left(0.39\right){\varvec{\pi}}{{\it i}}}\end{array}\right),\left(\begin{array}{c}0.48{e}^{\left(0.39\right){\varvec{\pi}}{{\it i}}},\\ 0.41{e}^{\left(0.40\right){\varvec{\pi}}{{\it i}}},\\ 0.40{e}^{\left(0.45\right){\varvec{\pi}}{{\it i}}}\end{array}\right),\left(\begin{array}{c}0.53{e}^{\left(0.45\right){\varvec{\pi}}{{\it i}}},\\ 0.41{e}^{\left(0.33\right){\varvec{\pi}}{{\it i}}},\\ 0.38{e}^{\left(0.48\right){\varvec{\pi}}{{\it i}}}\end{array}\right),\left(\begin{array}{c}0.51{e}^{\left(0.30\right){\varvec{\pi}}{{\it i}}},\\ 0.49{e}^{\left(0.29\right){\varvec{\pi}}{{\it i}}},\\ 0.43{e}^{\left(0.46\right){\varvec{\pi}}{{\it i}}}\end{array}\right)\right)\end{array}\right\}.$$

### Definition 26

A CSFSR $\raisebox{-4.5pt}{{\rm R}}\rotatebox{45}{\hspace*{-11pt}--}$ on $F$ is called CSFS equivalence class of $s$ modulo $\raisebox{-4.5pt}{{\rm R}}\rotatebox{45}{\hspace*{-11pt}--}$ is defined as,

$${\raisebox{-4.5pt}{{\rm R}}\rotatebox{45}{\hspace*{-11pt}--}}\left[s\right]=\left\{\left(s,{{\psi }_{{\acute{\text{\AA}}}}}_{c}\left(s\right),{{\mu }_{{\acute{\text{\AA}}}}}_{c}\left(s\right),{{\eta }_{{\acute{\text{\AA}}}}}_{c}\left(s\right)\right):\left(\left(f,s\right),\left({{\psi }_{{\acute{\text{\AA}}}}}_{c}\left(f,s\right)\right),\left({{\mu }_{{\acute{\text{\AA}}}}}_{c}\left(f,s\right)\right),\left({{\eta }_{{\acute{\text{\AA}}}}}_{c}\left(f,s\right)\right)\right)\in {\raisebox{-4.5pt}{{\rm R}}\rotatebox{45}{\hspace*{-11pt}--}}\right\}.$$

### Example 18

Table 1 shows the CP of CSFSR. The CSFS-equivalence-R is,

$${\raisebox{-4.5pt}{{\rm R}}\rotatebox{45}{\hspace*{-11pt}--}}=\left\{\begin{array}{c}\left(\left({s}_{1},{s}_{1}\right),\left(\begin{array}{c}0.44{e}^{\left(0.29\right){\varvec{\pi}}{{\it i}}},\\ 0.35{e}^{\left(0.30\right){\varvec{\pi}}{{\it i}}},\\ 0.34{e}^{\left(0.40\right){\varvec{\pi}}{{\it i}}}\end{array}\right),\left(\begin{array}{c}0.48{e}^{\left(0.41\right){\varvec{\pi}}{{\it i}}},\\ 0.41{e}^{\left(0.40\right){\varvec{\pi}}{{\it i}}},\\ 0.39{e}^{\left(0.45\right){\varvec{\pi}}{{\it i}}}\end{array}\right),\left(\begin{array}{c}0.52{e}^{\left(0.41\right){\varvec{\pi}}{{\it i}}},\\ 0.53{e}^{\left(0.33\right){\varvec{\pi}}{{\it i}}},\\ 0.51{e}^{\left(0.48\right){\varvec{\pi}}{{\it i}}}\end{array}\right),\left(\begin{array}{c}0.51{e}^{\left(0.30\right){\varvec{\pi}}{{\it i}}},\\ 0.35{e}^{\left(0.29\right){\varvec{\pi}}{{\it i}}},\\ 0.43{e}^{\left(0.49\right){\varvec{\pi}}{{\it i}}}\end{array}\right)\right),\\ \left(\left({s}_{2},{s}_{3}\right),\left(\begin{array}{c}0.48{e}^{(0.31){\varvec{\pi}}{{\it i}}},\\ 0.46{e}^{(0.23){\varvec{\pi}}{{\it i}}},\\ 0.40{e}^{\left(0.39\right){\varvec{\pi}}{{\it i}}}\end{array}\right),\left(\begin{array}{c}0.49{e}^{\left(0.23\right){\varvec{\pi}}{{\it i}}},\\ 0.54{e}^{\left(0.39\right){\varvec{\pi}}{{\it i}}},\\ 0.40{e}^{\left(0.45\right){\varvec{\pi}}{{\it i}}}\end{array}\right),\left(\begin{array}{c}0.46{e}^{\left(0.34\right){\varvec{\pi}}{{\it i}}},\\ 0.35{e}^{\left(0.46\right){\varvec{\pi}}{{\it i}}},\\ 0.38{e}^{\left(0.40\right){\varvec{\pi}}{{\it i}}}\end{array}\right),\left(\begin{array}{c}0.49{e}^{\left(0.36\right){\varvec{\pi}}{{\it i}}},\\ 0.43{e}^{\left(0.41\right){\varvec{\pi}}{{\it i}}},\\ 0.43{e}^{\left(0.48\right){\varvec{\pi}}{{\it i}}}\end{array}\right)\right),\\ \left(\left({s}_{2,}{s}_{2}\right),\left(\begin{array}{c}0.48{e}^{\left(0.31\right){\varvec{\pi}}{{\it i}}},\\ 0.49{e}^{\left(0.23\right){\varvec{\pi}}{{\it i}}},\\ 0.37{e}^{\left(0.40\right){\varvec{\pi}}{{\it i}}}\end{array}\right),\left(\begin{array}{c}0.49{e}^{\left(0.23\right){\varvec{\pi}}{{\it i}}},\\ 0.49{e}^{\left(0.38\right){\varvec{\pi}}{{\it i}}},\\ 0.41{e}^{\left(0.40\right){\varvec{\pi}}{{\it i}}}\end{array}\right),\left(\begin{array}{c}0.46{e}^{\left(0.34\right){\varvec{\pi}}{{\it i}}},\\ 0.35{e}^{\left(0.39\right){\varvec{\pi}}{{\it i}}},\\ 0.38{e}^{\left(0.39\right){\varvec{\pi}}{{\it i}}}\end{array}\right),\left(\begin{array}{c}0.49{e}^{\left(0.36\right){\varvec{\pi}}{{\it i}}},\\ 0.35{e}^{\left(0.47\right){\varvec{\pi}}{{\it i}}},\\ 0.45{e}^{\left(0.51\right){\varvec{\pi}}{{\it i}}}\end{array}\right)\right),\\ \left(\left({s}_{3},{s}_{2}\right),\left(\begin{array}{c}0.55{e}^{(0.34){\varvec{\pi}}{{\it i}}},\\ 0.45{e}^{(0.39){\varvec{\pi}}{{\it i}}},\\ 0.37{e}^{\left(0.40\right){\varvec{\pi}}{{\it i}}}\end{array}\right),\left(\begin{array}{c}0.51{e}^{\left(0.40\right){\varvec{\pi}}{{\it i}}},\\ 0.49{e}^{\left(0.33\right){\varvec{\pi}}{{\it i}}},\\ 0.41{e}^{\left(0.40\right){\varvec{\pi}}{{\it i}}}\end{array}\right),\left(\begin{array}{c}0.53{e}^{\left(0.41\right){\varvec{\pi}}{{\it i}}},\\ 0.42{e}^{\left(0.39\right){\varvec{\pi}}{{\it i}}},\\ 0.38{e}^{\left(0.39\right){\varvec{\pi}}{{\it i}}}\end{array}\right),\left(\begin{array}{c}0.54{e}^{\left(0.41\right){\varvec{\pi}}{{\it i}}},\\ 0.35{e}^{\left(0.41\right){\varvec{\pi}}{{\it i}}},\\ 0.45{e}^{\left(0.51\right){\varvec{\pi}}{{\it i}}}\end{array}\right)\right),\\ \left(\left({s}_{3},{s}_{3}\right),\left(\begin{array}{c}0.55{e}^{(0.34){\varvec{\pi}}{{\it i}}},\\ 0.45{e}^{(0.39){\varvec{\pi}}{{\it i}}},\\ 0.40{e}^{\left(0.40\right){\varvec{\pi}}{{\it i}}}\end{array}\right),\left(\begin{array}{c}0.52{e}^{\left(0.39\right){\varvec{\pi}}{{\it i}}},\\ 0.39{e}^{\left(0.33\right){\varvec{\pi}}{{\it i}}},\\ 0.41{e}^{\left(0.45\right){\varvec{\pi}}{{\it i}}}\end{array}\right),\left(\begin{array}{c}0.53{e}^{\left(0.45\right){\varvec{\pi}}{{\it i}}},\\ 0.41{e}^{\left(0.46\right){\varvec{\pi}}{{\it i}}},\\ 0.39{e}^{\left(0.40\right){\varvec{\pi}}{{\it i}}}\end{array}\right),\left(\begin{array}{c}0.54{e}^{\left(0.46\right){\varvec{\pi}}{{\it i}}},\\ 0.51{e}^{\left(0.41\right){\varvec{\pi}}{{\it i}}},\\ 0.43{e}^{\left(0.46\right){\varvec{\pi}}{{\it i}}}\end{array}\right)\right)\end{array}\right\}.$$

Then the CSFS equivalence classes are,

1. ${s}_{1}$ modulo $\raisebox{-4.5pt}{{\rm R}}\rotatebox{45}{\hspace*{-11pt}--}$ is given as
  $$\raisebox{-4.5pt}{{\rm R}}\rotatebox{45}{\hspace*{-11pt}--}\left[{s}_{1}\right]=\left\{\left({s}_{1},\left(\begin{array}{c}0.44{e}^{\left(0.29\right){\varvec{\pi}}{{\it i}}},\\ 0.35{e}^{\left(0.30\right){\varvec{\pi}}{{\it i}}},\\ 0.34{e}^{\left(0.40\right){\varvec{\pi}}{{\it i}}}\end{array}\right),\left(\begin{array}{c}0.48{e}^{\left(0.41\right){\varvec{\pi}}{{\it i}}},\\ 0.41{e}^{\left(0.40\right){\varvec{\pi}}{{\it i}}},\\ 0.39{e}^{\left(0.45\right){\varvec{\pi}}{{\it i}}}\end{array}\right),\left(\begin{array}{c}0.52{e}^{\left(0.41\right){\varvec{\pi}}{{\it i}}},\\ 0.53{e}^{\left(0.33\right){\varvec{\pi}}{{\it i}}},\\ 0.51{e}^{\left(0.48\right){\varvec{\pi}}{{\it i}}}\end{array}\right),\left(\begin{array}{c}0.51{e}^{\left(0.30\right){\varvec{\pi}}{{\it i}}},\\ 0.35{e}^{\left(0.29\right){\varvec{\pi}}{{\it i}}},\\ 0.43{e}^{\left(0.49\right){\varvec{\pi}}{{\it i}}}\end{array}\right)\right)\right\}.$$
2. ${s}_{2}$ modulo $\raisebox{-4.5pt}{{\rm R}}\rotatebox{45}{\hspace*{-11pt}--}$ is given as
  $$\raisebox{-4.5pt}{{\rm R}}\rotatebox{45}{\hspace*{-11pt}--}\left[{s}_{2}\right]=\left\{\begin{array}{c}\left({s}_{3} ,\left(\begin{array}{c}0.55{e}^{(0.34){\varvec{\pi}}{{\it i}}},\\ 0.45{e}^{(0.39){\varvec{\pi}}{{\it i}}},\\ 0.40{e}^{\left(0.40\right){\varvec{\pi}}{{\it i}}}\end{array}\right),\left(\begin{array}{c}0.52{e}^{\left(0.39\right){\varvec{\pi}}{{\it i}}},\\ 0.39{e}^{\left(0.33\right){\varvec{\pi}}{{\it i}}},\\ 0.41{e}^{\left(0.45\right){\varvec{\pi}}{{\it i}}}\end{array}\right),\left(\begin{array}{c}0.53{e}^{\left(0.45\right){\varvec{\pi}}{{\it i}}},\\ 0.41{e}^{\left(0.46\right){\varvec{\pi}}{{\it i}}},\\ 0.39{e}^{\left(0.40\right){\varvec{\pi}}{{\it i}}}\end{array}\right),\left(\begin{array}{c}0.54{e}^{\left(0.46\right){\varvec{\pi}}{{\it i}}},\\ 0.51{e}^{\left(0.41\right){\varvec{\pi}}{{\it i}}},\\ 0.43{e}^{\left(0.46\right){\varvec{\pi}}{{\it i}}}\end{array}\right)\right),\\ \left({s}_{2} ,\left(\begin{array}{c}0.55{e}^{(0.34){\varvec{\pi}}{{\it i}}},\\ 0.45{e}^{(0.39){\varvec{\pi}}{{\it i}}},\\ 0.37{e}^{\left(0.40\right){\varvec{\pi}}{{\it i}}}\end{array}\right),\left(\begin{array}{c}0.51{e}^{\left(0.40\right){\varvec{\pi}}{{\it i}}},\\ 0.49{e}^{\left(0.33\right){\varvec{\pi}}{{\it i}}},\\ 0.41{e}^{\left(0.40\right){\varvec{\pi}}{{\it i}}}\end{array}\right),\left(\begin{array}{c}0.53{e}^{\left(0.41\right){\varvec{\pi}}{{\it i}}},\\ 0.42{e}^{\left(0.39\right){\varvec{\pi}}{{\it i}}},\\ 0.38{e}^{\left(0.39\right){\varvec{\pi}}{{\it i}}}\end{array}\right),\left(\begin{array}{c}0.54{e}^{\left(0.41\right){\varvec{\pi}}{{\it i}}},\\ 0.35{e}^{\left(0.41\right){\varvec{\pi}}{{\it i}}},\\ 0.45{e}^{\left(0.51\right){\varvec{\pi}}{{\it i}}}\end{array}\right)\right)\end{array}\right\}.$$
3. ${s}_{3}$ modulo $\raisebox{-4.5pt}{{\rm R}}\rotatebox{45}{\hspace*{-11pt}--}$ is given as
  $$\raisebox{-4.5pt}{{\rm R}}\rotatebox{45}{\hspace*{-11pt}--}\left[{s}_{3}\right]=\left\{\begin{array}{c}\left({s}_{2} ,\left(\begin{array}{c}0.48{e}^{\left(0.31\right){\varvec{\pi}}{{\it i}}},\\ 0.49{e}^{\left(0.23\right){\varvec{\pi}}{{\it i}}},\\ 0.37{e}^{\left(0.40\right){\varvec{\pi}}{{\it i}}}\end{array}\right),\left(\begin{array}{c}0.49{e}^{\left(0.23\right){\varvec{\pi}}{{\it i}}},\\ 0.49{e}^{\left(0.38\right){\varvec{\pi}}{{\it i}}},\\ 0.41{e}^{\left(0.40\right){\varvec{\pi}}{{\it i}}}\end{array}\right),\left(\begin{array}{c}0.46{e}^{\left(0.34\right){\varvec{\pi}}{{\it i}}},\\ 0.35{e}^{\left(0.39\right){\varvec{\pi}}{{\it i}}},\\ 0.38{e}^{\left(0.39\right){\varvec{\pi}}{{\it i}}}\end{array}\right),\left(\begin{array}{c}0.49{e}^{\left(0.36\right){\varvec{\pi}}{{\it i}}},\\ 0.35{e}^{\left(0.47\right){\varvec{\pi}}{{\it i}}},\\ 0.45{e}^{\left(0.51\right){\varvec{\pi}}{{\it i}}}\end{array}\right)\right),\\ \left({s}_{3} ,\left(\begin{array}{c}0.55{e}^{(0.34){\varvec{\pi}}{{\it i}}},\\ 0.45{e}^{(0.39){\varvec{\pi}}{{\it i}}},\\ 0.40{e}^{\left(0.40\right){\varvec{\pi}}{{\it i}}}\end{array}\right),\left(\begin{array}{c}0.52{e}^{\left(0.39\right){\varvec{\pi}}{{\it i}}},\\ 0.39{e}^{\left(0.33\right){\varvec{\pi}}{{\it i}}},\\ 0.41{e}^{\left(0.45\right){\varvec{\pi}}{{\it i}}}\end{array}\right),\left(\begin{array}{c}0.53{e}^{\left(0.45\right){\varvec{\pi}}{{\it i}}},\\ 0.41{e}^{\left(0.46\right){\varvec{\pi}}{{\it i}}},\\ 0.39{e}^{\left(0.40\right){\varvec{\pi}}{{\it i}}}\end{array}\right),\left(\begin{array}{c}0.54{e}^{\left(0.46\right){\varvec{\pi}}{{\it i}}},\\ 0.51{e}^{\left(0.41\right){\varvec{\pi}}{{\it i}}},\\ 0.43{e}^{\left(0.46\right){\varvec{\pi}}{{\it i}}}\end{array}\right)\right)\end{array}\right\}.$$

### Definition 27

For CSFSR $\raisebox{-4.5pt}{{\rm R}}\rotatebox{45}{\hspace*{-11pt}--}$ on a CSFSS $F,$ then the CSFS-composite-R ${\raisebox{-4.5pt}{{\rm R}}\rotatebox{45}{\hspace*{-11pt}--}}_{1}\circ {\raisebox{-4.5pt}{{\rm R}}\rotatebox{45}{\hspace*{-11pt}--}}_{2}$ is defined as,

$$\left(\left(s,f\right),{{\psi }_{{\acute{\text{\AA}}}}}_{c}\left(s,f\right),{{\mu }_{{\acute{\text{\AA}}}}}_{c}\left(s,f\right),{{\eta }_{{\acute{\text{\AA}}}}}_{c}\left(s,f\right)\right)\in {\raisebox{-4.5pt}{{\rm R}}\rotatebox{45}{\hspace*{-11pt}--}}_{1}and\left(\left(f,g{\hspace*{-7pt}\neg}\right),{{\psi }_{{\acute{\text{\AA}}}}}_{c}\left(f,g{\hspace*{-7pt}\neg}\right),{{\mu }_{{\acute{\text{\AA}}}}}_{c}\left(f,g{\hspace*{-7pt}\neg}\right),{{\eta }_{{\acute{\text{\AA}}}}}_{c}\left(f,g{\hspace*{-7pt}\neg}\right)\right)\in {\raisebox{-4.5pt}{{\rm R}}\rotatebox{45}{\hspace*{-11pt}--}}_{2},$$

$$  \Rightarrow  \left(\left(s,g{\hspace*{-7pt}\neg}\right),{{\psi }_{{\acute{\text{\AA}}}}}_{c}\left(s,g{\hspace*{-7pt}\neg}\right),{{\mu }_{{\acute{\text{\AA}}}}}_{c}\left(s,g{\hspace*{-7pt}\neg}\right),{{\eta }_{{\acute{\text{\AA}}}}}_{c}\left(s,g{\hspace*{-7pt}\neg}\right)\right)\in {\raisebox{-4.5pt}{{\rm R}}\rotatebox{45}{\hspace*{-11pt}--}}_{1}\circ {\raisebox{-4.5pt}{{\rm R}}\rotatebox{45}{\hspace*{-11pt}--}}_{2}.$$

### Example 19

Table 1 shows the CP of CSFSR. We get two relations ${\raisebox{-4.5pt}{{\rm R}}\rotatebox{45}{\hspace*{-11pt}--}}_{1}$ and ${\raisebox{-4.5pt}{{\rm R}}\rotatebox{45}{\hspace*{-11pt}--}}_{2}$ from Table 1

$${\raisebox{-4.5pt}{{\rm R}}\rotatebox{45}{\hspace*{-11pt}--}}_{1}=\left\{\begin{array}{c}\left(\left({s}_{1},{s}_{2}\right),\left(\begin{array}{c}0.44{e}^{(0.29){\varvec{\pi}}{{\it i}}},\\ 0.40{e}^{(0.30){\varvec{\pi}}{{\it i}}},\\ 0.37{e}^{\left(0.40\right){\varvec{\pi}}{{\it i}}}\end{array}\right),\left(\begin{array}{c}0.48{e}^{\left(0.40\right){\varvec{\pi}}{{\it i}}},\\ 0.41{e}^{\left(0.38\right){\varvec{\pi}}{{\it i}}},\\ 0.41{e}^{\left(0.40\right){\varvec{\pi}}{{\it i}}}\end{array}\right),\left(\begin{array}{c}0.53{e}^{\left(0.41\right){\varvec{\pi}}{{\it i}}},\\ 0.52{e}^{\left(0.33\right){\varvec{\pi}}{{\it i}}},\\ 0.38{e}^{\left(0.48\right){\varvec{\pi}}{{\it i}}}\end{array}\right),\left(\begin{array}{c}0.51{e}^{\left(0.30\right){\varvec{\pi}}{{\it i}}},\\ 0.35{e}^{\left(0.29\right){\varvec{\pi}}{{\it i}}},\\ 0.45{e}^{\left(0.51\right){\varvec{\pi}}{{\it i}}}\end{array}\right)\right),\\ \left(\left({s}_{2},{s}_{3}\right),\left(\begin{array}{c}0.48{e}^{(0.31){\varvec{\pi}}{{\it i}}},\\ 0.46{e}^{(0.23){\varvec{\pi}}{{\it i}}},\\ 0.40{e}^{\left(0.39\right){\varvec{\pi}}{{\it i}}}\end{array}\right),\left(\begin{array}{c}0.49{e}^{\left(0.23\right){\varvec{\pi}}{{\it i}}},\\ 0.54{e}^{\left(0.39\right){\varvec{\pi}}{{\it i}}},\\ 0.40{e}^{\left(0.45\right){\varvec{\pi}}{{\it i}}}\end{array}\right),\left(\begin{array}{c}0.46{e}^{\left(0.34\right){\varvec{\pi}}{{\it i}}},\\ 0.35{e}^{\left(0.46\right){\varvec{\pi}}{{\it i}}},\\ 0.38{e}^{\left(0.40\right){\varvec{\pi}}{{\it i}}}\end{array}\right),\left(\begin{array}{c}0.49{e}^{\left(0.36\right){\varvec{\pi}}{{\it i}}},\\ 0.43{e}^{\left(0.41\right){\varvec{\pi}}{{\it i}}},\\ 0.43{e}^{\left(0.48\right){\varvec{\pi}}{{\it i}}}\end{array}\right)\right),\\ \left(\left({s}_{1},{s}_{3}\right),\left(\begin{array}{c}0.44{e}^{(0.29){\varvec{\pi}}{{\it i}}},\\ 0.40{e}^{(0.30){\varvec{\pi}}{{\it i}}},\\ 0.40{e}^{\left(0.39\right){\varvec{\pi}}{{\it i}}}\end{array}\right),\left(\begin{array}{c}0.48{e}^{\left(0.39\right){\varvec{\pi}}{{\it i}}},\\ 0.41{e}^{\left(0.40\right){\varvec{\pi}}{{\it i}}},\\ 0.40{e}^{\left(0.45\right){\varvec{\pi}}{{\it i}}}\end{array}\right),\left(\begin{array}{c}0.53{e}^{\left(0.45\right){\varvec{\pi}}{{\it i}}},\\ 0.41{e}^{\left(0.33\right){\varvec{\pi}}{{\it i}}},\\ 0.38{e}^{\left(0.48\right){\varvec{\pi}}{{\it i}}}\end{array}\right),\left(\begin{array}{c}0.51{e}^{\left(0.30\right){\varvec{\pi}}{{\it i}}},\\ 0.49{e}^{\left(0.29\right){\varvec{\pi}}{{\it i}}},\\ 0.43{e}^{\left(0.46\right){\varvec{\pi}}{{\it i}}}\end{array}\right)\right)\end{array}\right\},$$

and

$${\raisebox{-4.5pt}{{\rm R}}\rotatebox{45}{\hspace*{-11pt}--}}_{2}=\left\{\begin{array}{c}\left(\left({s}_{2,}{s}_{2}\right),\left(\begin{array}{c}0.48{e}^{\left(0.31\right){\varvec{\pi}}{{\it i}}},\\ 0.49{e}^{\left(0.23\right){\varvec{\pi}}{{\it i}}},\\ 0.37{e}^{\left(0.40\right){\varvec{\pi}}{{\it i}}}\end{array}\right),\left(\begin{array}{c}0.49{e}^{\left(0.23\right){\varvec{\pi}}{{\it i}}},\\ 0.49{e}^{\left(0.38\right){\varvec{\pi}}{{\it i}}},\\ 0.41{e}^{\left(0.40\right){\varvec{\pi}}{{\it i}}}\end{array}\right),\left(\begin{array}{c}0.46{e}^{\left(0.34\right){\varvec{\pi}}{{\it i}}},\\ 0.35{e}^{\left(0.39\right){\varvec{\pi}}{{\it i}}},\\ 0.38{e}^{\left(0.39\right){\varvec{\pi}}{{\it i}}}\end{array}\right),\left(\begin{array}{c}0.49{e}^{\left(0.36\right){\varvec{\pi}}{{\it i}}},\\ 0.35{e}^{\left(0.47\right){\varvec{\pi}}{{\it i}}},\\ 0.45{e}^{\left(0.51\right){\varvec{\pi}}{{\it i}}}\end{array}\right)\right),\\ \left(\left({s}_{3},{s}_{1}\right),\left(\begin{array}{c}0.49{e}^{\left(0.34\right){\varvec{\pi}}{{\it i}}},\\ 0.35{e}^{\left(0.39\right){\varvec{\pi}}{{\it i}}},\\ 0.35{e}^{\left(0.40\right){\varvec{\pi}}{{\it i}}}\end{array}\right),\left(\begin{array}{c}0.58{e}^{\left(0.41\right){\varvec{\pi}}{{\it i}}},\\ 0.47{e}^{\left(0.33\right){\varvec{\pi}}{{\it i}}},\\ 0.41{e}^{\left(0.45\right){\varvec{\pi}}{{\it i}}}\end{array}\right),\left(\begin{array}{c}0.52{e}^{\left(0.41\right){\varvec{\pi}}{{\it i}}},\\ 0.42{e}^{\left(0.40\right){\varvec{\pi}}{{\it i}}},\\ 0.51{e}^{\left(0.40\right){\varvec{\pi}}{{\it i}}}\end{array}\right),\left(\begin{array}{c}0.54{e}^{\left(0.40\right){\varvec{\pi}}{{\it i}}},\\ 0.35{e}^{\left(0.35\right){\varvec{\pi}}{{\it i}}},\\ 0.43{e}^{\left(0.49\right){\varvec{\pi}}{{\it i}}}\end{array}\right)\right),\\ \left(\left({s}_{3},{s}_{3}\right),\left(\begin{array}{c}0.55{e}^{(0.34){\varvec{\pi}}{{\it i}}},\\ 0.45{e}^{(0.39){\varvec{\pi}}{{\it i}}},\\ 0.40{e}^{\left(0.40\right){\varvec{\pi}}{{\it i}}}\end{array}\right),\left(\begin{array}{c}0.52{e}^{\left(0.39\right){\varvec{\pi}}{{\it i}}},\\ 0.39{e}^{\left(0.33\right){\varvec{\pi}}{{\it i}}},\\ 0.41{e}^{\left(0.45\right){\varvec{\pi}}{{\it i}}}\end{array}\right),\left(\begin{array}{c}0.53{e}^{\left(0.45\right){\varvec{\pi}}{{\it i}}},\\ 0.41{e}^{\left(0.46\right){\varvec{\pi}}{{\it i}}},\\ 0.39{e}^{\left(0.40\right){\varvec{\pi}}{{\it i}}}\end{array}\right),\left(\begin{array}{c}0.54{e}^{\left(0.46\right){\varvec{\pi}}{{\it i}}},\\ 0.51{e}^{\left(0.41\right){\varvec{\pi}}{{\it i}}},\\ 0.43{e}^{\left(0.46\right){\varvec{\pi}}{{\it i}}}\end{array}\right)\right)\end{array}\right\}.$$

Then the CSFS-composite-R is given as,

$${\raisebox{-4.5pt}{{\rm R}}\rotatebox{45}{\hspace*{-11pt}--}}_{1}\circ {\raisebox{-4.5pt}{{\rm R}}\rotatebox{45}{\hspace*{-11pt}--}}_{2}=\left\{\begin{array}{c}\left(\left({s}_{1},{s}_{2}\right),\left(\begin{array}{c}0.44{e}^{\left(0.29\right){\varvec{\pi}}{{\it i}}},\\ 0.40{e}^{\left(0.30\right){\varvec{\pi}}{{\it i}}},\\ 0.37{e}^{\left(0.40\right){\varvec{\pi}}{{\it i}}}\end{array}\right),\left(\begin{array}{c}0.48{e}^{\left(0.40\right){\varvec{\pi}}{{\it i}}},\\ 0.41{e}^{\left(0.38\right){\varvec{\pi}}{{\it i}}},\\ 0.41{e}^{\left(0.40\right){\varvec{\pi}}{{\it i}}}\end{array}\right),\left(\begin{array}{c}0.53{e}^{\left(0.41\right){\varvec{\pi}}{{\it i}}},\\ 0.52{e}^{\left(0.33\right){\varvec{\pi}}{{\it i}}},\\ 0.38{e}^{\left(0.48\right){\varvec{\pi}}{{\it i}}}\end{array}\right),\left(\begin{array}{c}0.51{e}^{\left(0.30\right){\varvec{\pi}}{{\it i}}},\\ 0.35{e}^{\left(0.29\right){\varvec{\pi}}{{\it i}}},\\ 0.45{e}^{\left(0.51\right){\varvec{\pi}}{{\it i}}}\end{array}\right)\right),\\ \left(\left({s}_{2},{s}_{1}\right),\left(\begin{array}{c}0.48{e}^{\left(0.31\right){\varvec{\pi}}{{\it i}}},\\ 0.35{e}^{\left(0.23\right){\varvec{\pi}}{{\it i}}},\\ 0.34{e}^{\left(0.40\right){\varvec{\pi}}{{\it i}}}\end{array}\right),\left(\begin{array}{c}0.49{e}^{\left(0.23\right){\varvec{\pi}}{{\it i}}},\\ 0.47{e}^{\left(0.39\right){\varvec{\pi}}{{\it i}}},\\ 0.39{e}^{\left(0.45\right){\varvec{\pi}}{{\it i}}}\end{array}\right),\left(\begin{array}{c}0.46{e}^{\left(0.34\right){\varvec{\pi}}{{\it i}}},\\ 0.35{e}^{\left(0.40\right){\varvec{\pi}}{{\it i}}},\\ 0.51{e}^{\left(0.39\right){\varvec{\pi}}{{\it i}}}\end{array}\right),\left(\begin{array}{c}0.49{e}^{\left(0.36\right){\varvec{\pi}}{{\it i}}},\\ 0.35{e}^{\left(0.35\right){\varvec{\pi}}{{\it i}}},\\ 0.43{e}^{\left(0.49\right){\varvec{\pi}}{{\it i}}}\end{array}\right)\right),\\ \left(\left({s}_{1},{s}_{3}\right),\left(\begin{array}{c}0.44{e}^{\left(0.29\right){\varvec{\pi}}{{\it i}}},\\ 0.40{e}^{\left(0.30\right){\varvec{\pi}}{{\it i}}},\\ 0.40{e}^{\left(0.39\right){\varvec{\pi}}{{\it i}}}\end{array}\right),\left(\begin{array}{c}0.48{e}^{\left(0.39\right){\varvec{\pi}}{{\it i}}},\\ 0.41{e}^{\left(0.40\right){\varvec{\pi}}{{\it i}}},\\ 0.40{e}^{\left(0.45\right){\varvec{\pi}}{{\it i}}}\end{array}\right),\left(\begin{array}{c}0.53{e}^{\left(0.45\right){\varvec{\pi}}{{\it i}}},\\ 0.41{e}^{\left(0.33\right){\varvec{\pi}}{{\it i}}},\\ 0.38{e}^{\left(0.48\right){\varvec{\pi}}{{\it i}}}\end{array}\right),\left(\begin{array}{c}0.51{e}^{\left(0.30\right){\varvec{\pi}}{{\it i}}},\\ 0.49{e}^{\left(0.29\right){\varvec{\pi}}{{\it i}}},\\ 0.43{e}^{\left(0.46\right){\varvec{\pi}}{{\it i}}}\end{array}\right)\right)\end{array}\right\}.$$

### Theorem 1

A CSFSR $\raisebox{-4.5pt}{{\rm R}}\rotatebox{45}{\hspace*{-11pt}--}$ is a CSFS-symmetric-R on a CSFSS $F$ iff ${\raisebox{-4.5pt}{{\rm R}}\rotatebox{45}{\hspace*{-11pt}--}}=\raisebox{-4.5pt}{{\rm R}}\rotatebox{45}{\hspace*{-11pt}--}^{c}.$

### Proof

Suppose that ${\raisebox{-4.5pt}{{\rm R}}\rotatebox{45}{\hspace*{-11pt}--}}=\raisebox{-4.5pt}{{\rm R}}\rotatebox{45}{\hspace*{-11pt}--}^{c}$, then $\left(\left(s,f\right),{({\psi }_{{\acute{\text{\AA}}}}}_{c}\left(s,f\right)),\left({{\mu }_{{\acute{\text{\AA}}}}}_{c}\left(s,f\right)\right),\left({{\eta }_{{\acute{\text{\AA}}}}}_{c}\left(s,f\right)\right)\right)\in {\raisebox{-4.5pt}{{\rm R}}\rotatebox{45}{\hspace*{-11pt}--}},$

$$  \Rightarrow  \left(\left(f,s\right),{({\psi }_{{\acute{\text{\AA}}}}}_{c}\left(f,s\right)),\left({{\mu }_{{\acute{\text{\AA}}}}}_{c}\left(f,s\right)\right),\left({{\eta }_{{\acute{\text{\AA}}}}}_{c}\left(f,s\right)\right)\right)\in \raisebox{-4.5pt}{{\rm R}}\rotatebox{45}{\hspace*{-11pt}--}^{c},$$

$$  \Rightarrow  \left(\left(f,s\right),{({\psi }_{{\acute{\text{\AA}}}}}_{c}\left(f,s\right)),\left({{\mu }_{{\acute{\text{\AA}}}}}_{c}\left(f,s\right)\right),\left({{\eta }_{{\acute{\text{\AA}}}}}_{c}\left(f,s\right)\right)\right)\in {\raisebox{-4.5pt}{{\rm R}}\rotatebox{45}{\hspace*{-11pt}--}}.$$

Thus, ${\raisebox{-4.5pt}{{\rm R}}\rotatebox{45}{\hspace*{-11pt}--}}$ is a CSFS-symmetric-R on a CSFSS $F$.

Conversely, assume that ${\raisebox{-4.5pt}{{\rm R}}\rotatebox{45}{\hspace*{-11pt}--}}$ is a CSFS-symmetric-R on a CSFSS $F$, then

$$\left(\left(s,f\right),{({\psi }_{{\acute{\text{\AA}}}}}_{c}\left(s,f\right)),\left({{\mu }_{{\acute{\text{\AA}}}}}_{c}\left(s,f\right)\right),\left({{\eta }_{{\acute{\text{\AA}}}}}_{c}\left(s,f\right)\right)\right)\in {\raisebox{-4.5pt}{{\rm R}}\rotatebox{45}{\hspace*{-11pt}--}}  \Rightarrow  \left(\left(f,s\right),{({\psi }_{{\acute{\text{\AA}}}}}_{c}\left(f,s\right)),\left({{\mu }_{{\acute{\text{\AA}}}}}_{c}\left(f,s\right)\right),\left({{\eta }_{{\acute{\text{\AA}}}}}_{c}\left(f,s\right)\right)\right)\in {\raisebox{-4.5pt}{{\rm R}}\rotatebox{45}{\hspace*{-11pt}--}}.$$

However, $$\left(\left(f,s\right),{({\psi }_{{\acute{\text{\AA}}}}}_{c}\left(f,s\right)),\left({{\mu }_{{\acute{\text{\AA}}}}}_{c}\left(f,s\right)\right),\left({{\eta }_{{\acute{\text{\AA}}}}}_{c}\left(f,s\right)\right)\right)\in \raisebox{-4.5pt}{{\rm R}}\rotatebox{45}{\hspace*{-11pt}--}^{c}$$

$$  \Rightarrow  {\raisebox{-4.5pt}{{\rm R}}\rotatebox{45}{\hspace*{-11pt}--}}=\raisebox{-4.5pt}{{\rm R}}\rotatebox{45}{\hspace*{-11pt}--}^{c} .$$

### Theorem 2

CSFSR ${\raisebox{-4.5pt}{{\rm R}}\rotatebox{45}{\hspace*{-11pt}--}}$ is a CSFS-transitive-R on a CSFSS $F$ iff ${\raisebox{-4.5pt}{{\rm R}}\rotatebox{45}{\hspace*{-11pt}--}}\circ {\raisebox{-4.5pt}{{\rm R}}\rotatebox{45}{\hspace*{-11pt}--}}\subseteq \raisebox{-4.5pt}{{\rm R}}\rotatebox{45}{\hspace*{-11pt}--}^{c}.$

### Proof

Suppose that ${\raisebox{-4.5pt}{{\rm R}}\rotatebox{45}{\hspace*{-11pt}--}}$ is a CSFS-transitive-R on a CSFSS $F$.

Let $$\left(\left(s,g{\hspace*{-7pt}\neg}\right),\left({({\psi }_{{\acute{\text{\AA}}}}}_{c}\left(s,g{\hspace*{-7pt}\neg}\right)\right),\left({{\mu }_{{\acute{\text{\AA}}}}}_{c}\left(s,g{\hspace*{-7pt}\neg}\right)\right),\left({{\eta }_{{\acute{\text{\AA}}}}}_{c}\left(s,g{\hspace*{-7pt}\neg}\right)\right)\right)\in {\raisebox{-4.5pt}{{\rm R}}\rotatebox{45}{\hspace*{-11pt}--}}\circ {\raisebox{-4.5pt}{{\rm R}}\rotatebox{45}{\hspace*{-11pt}--}},$$.

Then, by the definition of CSFS-transitive-R,

$$\left(\left(s,f\right),{({\psi }_{{\acute{\text{\AA}}}}}_{c}\left(s,f\right)),\left({{\mu }_{{\acute{\text{\AA}}}}}_{c}\left(s,f\right)\right),\left({{\eta }_{{\acute{\text{\AA}}}}}_{c}\left(s,f\right)\right)\right)\in {\raisebox{-4.5pt}{{\rm R}}\rotatebox{45}{\hspace*{-11pt}--}}\text{ and }\left(\left(f,g{\hspace*{-7pt}\neg}\right),{({\psi }_{{\acute{\text{\AA}}}}}_{c}\left(f,g{\hspace*{-7pt}\neg}\right)),\left({{\mu }_{{\acute{\text{\AA}}}}}_{c}\left(f,g{\hspace*{-7pt}\neg}\right)\right),\left({{\eta }_{{\acute{\text{\AA}}}}}_{c}\left(f,g{\hspace*{-7pt}\neg}\right)\right)\right)\in {\raisebox{-4.5pt}{{\rm R}}\rotatebox{45}{\hspace*{-11pt}--}},$$

$$\left(\left(s,g{\hspace*{-7pt}\neg}\right),{({\psi }_{{\acute{\text{\AA}}}}}_{c}\left(s,g{\hspace*{-7pt}\neg}\right)),\left({{\mu }_{{\acute{\text{\AA}}}}}_{c}\left(s,g{\hspace*{-7pt}\neg}\right)\right),\left({{\eta }_{{\acute{\text{\AA}}}}}_{c}\left(s,g{\hspace*{-7pt}\neg}\right)\right)\right)\in {\raisebox{-4.5pt}{{\rm R}}\rotatebox{45}{\hspace*{-11pt}--}},$$

$$  \Rightarrow  {\raisebox{-4.5pt}{{\rm R}}\rotatebox{45}{\hspace*{-11pt}--}}\circ {\raisebox{-4.5pt}{{\rm R}}\rotatebox{45}{\hspace*{-11pt}--}}\subseteq {\raisebox{-4.5pt}{{\rm R}}\rotatebox{45}{\hspace*{-11pt}--}}.$$

Conversely, assume that ${\raisebox{-4.5pt}{{\rm R}}\rotatebox{45}{\hspace*{-11pt}--}}\circ {\raisebox{-4.5pt}{{\rm R}}\rotatebox{45}{\hspace*{-11pt}--}}\subseteq {\raisebox{-4.5pt}{{\rm R}}\rotatebox{45}{\hspace*{-11pt}--}},$ then

For $$\left(\left(s,f\right),{({\psi }_{{\acute{\text{\AA}}}}}_{c}\left(s,f\right)),\left({{\mu }_{{\acute{\text{\AA}}}}}_{c}\left(s,f\right)\right),\left({{\eta }_{{\acute{\text{\AA}}}}}_{c}\left(s,f\right)\right)\right)\in {\raisebox{-4.5pt}{{\rm R}}\rotatebox{45}{\hspace*{-11pt}--}} $$ and $$\left(\left(f,g{\hspace*{-7pt}\neg}\right),{({\psi }_{{\acute{\text{\AA}}}}}_{c}\left(f,g{\hspace*{-7pt}\neg}\right)),\left({{\mu }_{{\acute{\text{\AA}}}}}_{c}\left(f,g{\hspace*{-7pt}\neg}\right)\right),\left({{\eta }_{{\acute{\text{\AA}}}}}_{c}\left(f,g{\hspace*{-7pt}\neg}\right)\right)\right)\in {\raisebox{-4.5pt}{{\rm R}}\rotatebox{45}{\hspace*{-11pt}--}} $$

$$\left(\left(s,g{\hspace*{-7pt}\neg}\right),{({\psi }_{{\acute{\text{\AA}}}}}_{c}\left(s,g{\hspace*{-7pt}\neg}\right)),\left({{\mu }_{{\acute{\text{\AA}}}}}_{c}\left(s,g{\hspace*{-7pt}\neg}\right)\right),\left({{\eta }_{{\acute{\text{\AA}}}}}_{c}\left(s,g{\hspace*{-7pt}\neg}\right)\right)\right)\in {\raisebox{-4.5pt}{{\rm R}}\rotatebox{45}{\hspace*{-11pt}--}}\circ {\raisebox{-4.5pt}{{\rm R}}\rotatebox{45}{\hspace*{-11pt}--}}\subseteq {\raisebox{-4.5pt}{{\rm R}}\rotatebox{45}{\hspace*{-11pt}--}},$$

$$\left(\left(s,g{\hspace*{-7pt}\neg}\right),{({\psi }_{{\acute{\text{\AA}}}}}_{c}\left(s,g{\hspace*{-7pt}\neg}\right)),\left({{\mu }_{{\acute{\text{\AA}}}}}_{c}\left(s,g{\hspace*{-7pt}\neg}\right)\right),\left({{\eta }_{{\acute{\text{\AA}}}}}_{c}\left(s,g{\hspace*{-7pt}\neg}\right)\right)\right)\in {\raisebox{-4.5pt}{{\rm R}}\rotatebox{45}{\hspace*{-11pt}--}}.$$

Thus, ${\raisebox{-4.5pt}{{\rm R}}\rotatebox{45}{\hspace*{-11pt}--}}$ is a CSFS-transitive-R on a CSFSS $F$.

### Theorem 3

ACSFSR ${\raisebox{-4.5pt}{{\rm R}}\rotatebox{45}{\hspace*{-11pt}--}}$ is a CSFS-equivalence-R on a CSFSS $F,$ iff ${\raisebox{-4.5pt}{{\rm R}}\rotatebox{45}{\hspace*{-11pt}--}}\circ {\raisebox{-4.5pt}{{\rm R}}\rotatebox{45}{\hspace*{-11pt}--}}={\raisebox{-4.5pt}{{\rm R}}\rotatebox{45}{\hspace*{-11pt}--}}.$

### Proof

Suppose that

$$\left(\left(s,f\right),{({\psi }_{{\acute{\text{\AA}}}}}_{c}\left(s,f\right)),\left({{\mu }_{{\acute{\text{\AA}}}}}_{c}\left(s,f\right)\right),\left({{\eta }_{{\acute{\text{\AA}}}}}_{c}\left(s,f\right)\right)\right)\in {\raisebox{-4.5pt}{{\rm R}}\rotatebox{45}{\hspace*{-11pt}--}}.$$

Then by the definition of CSFS-symmetric-R,

$$\left(\left(f,s\right),{({\psi }_{{\acute{\text{\AA}}}}}_{c}\left(f,s\right)),\left({{\mu }_{{\acute{\text{\AA}}}}}_{c}\left(f,s\right)\right),\left({{\eta }_{{\acute{\text{\AA}}}}}_{c}\left(f,s\right)\right)\right)\in {\raisebox{-4.5pt}{{\rm R}}\rotatebox{45}{\hspace*{-11pt}--}}.$$

Now, by the definition of CSFS-transitive-R,

$$\left(\left(s,s\right),{({\psi }_{{\acute{\text{\AA}}}}}_{c}\left(s,s\right)),\left({{\mu }_{{\acute{\text{\AA}}}}}_{c}\left(s,s\right)\right),\left({{\eta }_{{\acute{\text{\AA}}}}}_{c}\left(s,s\right)\right)\right)\in {\raisebox{-4.5pt}{{\rm R}}\rotatebox{45}{\hspace*{-11pt}--}}.$$

However, by the definition of CSFS-composite-R,

$$\left(\left(s,s\right),{({\psi }_{{\acute{\text{\AA}}}}}_{c}\left(s,s\right)),\left({{\mu }_{{\acute{\text{\AA}}}}}_{c}\left(s,s\right)\right),\left({{\eta }_{{\acute{\text{\AA}}}}}_{c}\left(s,s\right)\right)\right)\in {\raisebox{-4.5pt}{{\rm R}}\rotatebox{45}{\hspace*{-11pt}--}}\circ {\raisebox{-4.5pt}{{\rm R}}\rotatebox{45}{\hspace*{-11pt}--}}.$$

Hence,

$${\raisebox{-4.5pt}{{\rm R}}\rotatebox{45}{\hspace*{-11pt}--}}\subseteq {\raisebox{-4.5pt}{{\rm R}}\rotatebox{45}{\hspace*{-11pt}--}}\circ {\raisebox{-4.5pt}{{\rm R}}\rotatebox{45}{\hspace*{-11pt}--}} \left(i\right).$$

Conversely, assume that

$$\left(\left(s,f\right),{({\psi }_{{\acute{\text{\AA}}}}}_{c}\left(s,f\right)),\left({{\mu }_{{\acute{\text{\AA}}}}}_{c}\left(s,f\right)\right),\left({{\eta }_{{\acute{\text{\AA}}}}}_{c}\left(s,f\right)\right)\right)\in {\raisebox{-4.5pt}{{\rm R}}\rotatebox{45}{\hspace*{-11pt}--}}\circ {\raisebox{-4.5pt}{{\rm R}}\rotatebox{45}{\hspace*{-11pt}--}}.$$

Then there exist

$$g{\hspace*{-7pt}\neg}\in {\acute{\text{\AA}}}\ni \left(\left(s,g{\hspace*{-7pt}\neg}\right),{({\psi }_{{\acute{\text{\AA}}}}}_{c}\left(s,g{\hspace*{-7pt}\neg}\right)),\left({{\mu }_{{\acute{\text{\AA}}}}}_{c}\left(s,g{\hspace*{-7pt}\neg}\right)\right),\left({{\eta }_{{\acute{\text{\AA}}}}}_{c}\left(s,g{\hspace*{-7pt}\neg}\right)\right)\right)\in {\raisebox{-4.5pt}{{\rm R}}\rotatebox{45}{\hspace*{-11pt}--}}\text{ and }\left(\left(g{\hspace*{-7pt}\neg},f\right),{({\psi }_{{\acute{\text{\AA}}}}}_{c}\left(g{\hspace*{-7pt}\neg},f\right)),\left({{\mu }_{{\acute{\text{\AA}}}}}_{c}\left(g{\hspace*{-7pt}\neg},f\right)\right),\left({{\eta }_{{\acute{\text{\AA}}}}}_{c}\left(g{\hspace*{-7pt}\neg},f\right)\right)\right)\in {\raisebox{-4.5pt}{{\rm R}}\rotatebox{45}{\hspace*{-11pt}--}}.$$

However, ${\raisebox{-4.5pt}{{\rm R}}\rotatebox{45}{\hspace*{-11pt}--}}$ is a CSFS-equivalence-R on a CSFSS ${\acute{\text{\AA}}},$ so ${\raisebox{-4.5pt}{{\rm R}}\rotatebox{45}{\hspace*{-11pt}--}}$ is also a CSFS-transitive-R. Therefore, $g{\hspace*{-7pt}\neg}$

$$\left(\left(s,f\right),{({\psi }_{{\acute{\text{\AA}}}}}_{c}\left(s,f\right)),\left({{\mu }_{{\acute{\text{\AA}}}}}_{c}\left(s,f\right)\right),\left({{\eta }_{{\acute{\text{\AA}}}}}_{c}\left(s,f\right)\right)\right)\in {\raisebox{-4.5pt}{{\rm R}}\rotatebox{45}{\hspace*{-11pt}--}},$$

$$  \Rightarrow  {\raisebox{-4.5pt}{{\rm R}}\rotatebox{45}{\hspace*{-11pt}--}}\circ {\raisebox{-4.5pt}{{\rm R}}\rotatebox{45}{\hspace*{-11pt}--}}\subseteq {\raisebox{-4.5pt}{{\rm R}}\rotatebox{45}{\hspace*{-11pt}--}} \left({\text{ii}}\right).$$

Hence, equation (i) and (ii),

$${\raisebox{-4.5pt}{{\rm R}}\rotatebox{45}{\hspace*{-11pt}--}}\circ {\raisebox{-4.5pt}{{\rm R}}\rotatebox{45}{\hspace*{-11pt}--}}={\raisebox{-4.5pt}{{\rm R}}\rotatebox{45}{\hspace*{-11pt}--}}.$$

## Applications

In this section, an application of the proposed ideas to select the best web-browser by utilizing the concept of CSFSRs and their types.

### Web browser

A web browser is a piece of software that is used to access content on the World Wide Web. The task of the web browser is to find the requested information on any website. You can also navigate anywhere on the internet with a web browser. The web browser retrieves the information and displays it on your computer or mobile device to start the process. The fact that this data comes from multiple websites illustrates how interconnected the World Wide Web is. The Hypertext Transfer Protocol (HTTP), a standardized protocol that controls the transmission of text, images, and video over the internet, is the essential mechanism enabling this data exchange. The rules and conventions for communication between web browsers and servers are set forth by HTTP, guaranteeing a smooth exchange of content.

**Figure 1:**
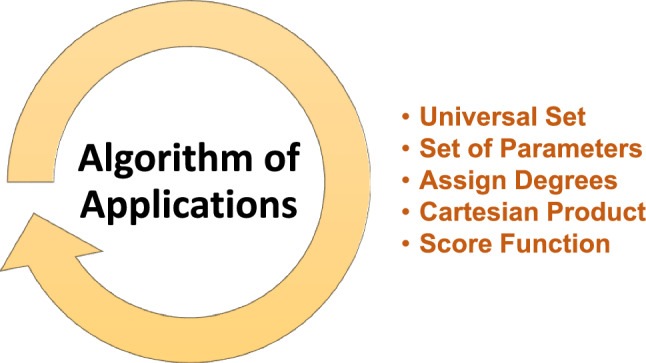
Algorithm of application.

The application’s algorithm, as shown in Fig. 1, shows the sequential process by which the data is analyzed and presented. This algorithm provides insights into the underlying structure and functionality of the application by visualizing the computational steps that it takes. This graphical representation captures the nuances of how data is retrieved, processed, and displayed on the user interface, providing a useful tool for both developers and users to understand the inner workings of the application. This entire process essentially emphasizes how web interactions are dynamic, with information moving across the digital landscape under the direction of the protocols and algorithms that support the operation of web applications. Our understanding of the complex dance between servers and browsers that makes it possible for content to be delivered to our screens smoothly is enhanced when we comprehend the function of HTTP and can visualize the application algorithm.

**Figure 2:**
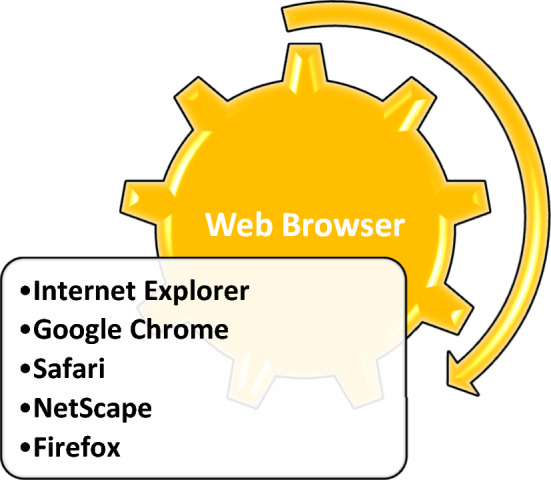
Summary of web browser.

Firstly, we express a universal set that it consists of four types of Web Browser. The universal set consists of four types of Web Browser i.e., ${\mathring{\rm{u}}}_{1}=$ Internet explorer, ${\mathring{\rm{u}}}_{2}=$ Google chrome and ${\mathring{\rm{u}}}_{3}=$ Safari, ${\mathring{\rm{u}}}_{4}=$ Net scape ${\mathring{\rm{u}}}_{5}=$ Firefox. The Fig. 2 discussed the types of Web Browser.

#### Internet explorer

The graphical web browser series known as Internet Explorer, created by Microsoft and utilised in conjunction with the Windows operating system, has been retired. It was initially released in 1995 as a part of the Plus! Windows 95 add-on package. Users can view websites on the internet with the free web browser, Internet Explorer. It is also known as IE or MSIE. Additionally, it is used to access online banking, internet marketing, streaming video, and many other things.

#### Google chrome

A cross-platform web browser created by Google is called Google Chrome. It was created using open-source software from Mozilla Firefox and Apple WebKit, and it was first made available in 2008 for Microsoft Windows. It later became the default browser on Linux, macOS, iOS, and Android. Free of charge, Google Chrome is a quick web browser. You can see if Chrome is compatible with your operating system and that you meet all the other system requirements before you download.

#### Safari

Apple created the graphical web browser known as Safari. It is based primarily on open-source code, particularly WebKit. It replaced Internet Explorer for Mac, Cyber dog, and Netscape Navigator as the standard web browser for Macintosh computers. On all of your Apple devices, Safari offers the best internet browsing experience. You can browse however you like, whenever you like, thanks to its extensive customization options, strong privacy protections, and industry-leading battery life. It is also the fastest browser in the world in terms of speed.

#### Netscape

A provider of computer services, Netscape Communications was best known for its Web browser, Navigator. Netscape was developed by Jim Clark and Marc Andreessen at Mosaic Communications Corp., which they established in April 1994. By introducing people to the future of the Web, Netscape also inspired Microsoft to develop its rival and market-dominating Internet Explorer browser. Mosaic Netscape is the original name of the Netscape browser.

#### Firefox

The Mozilla Foundation and its subsidiary, the Mozilla Corporation, created the free and open-source web browser known as Mozilla Firefox, or simply Firefox. It displays web pages using the Gecko rendering engine, which incorporates up-to-date and future web standards.

**Figure 3:**
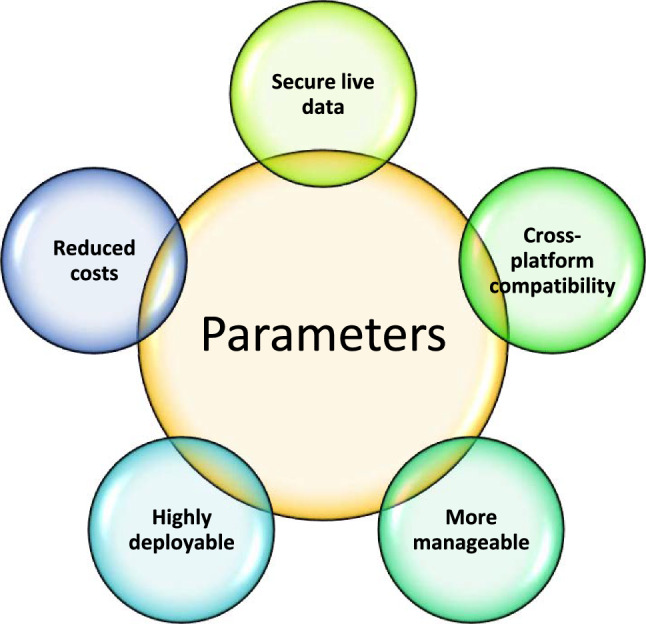
Summary of web browser parameters.

Secondly, describe the parameter of the web browser $\text{'}E=\left\{{s}_{1},{s}_{2},{s}_{3}, {s}_{4},{s}_{5}\right\}$ i.e. ${s}_{1}=$ Cross-plateform compatibility, ${s}_{2}=$ More manageable, ${s}_{3}=$ Highly deployable, ${s}_{4}=$ Secure live data, ${s}_{5}=$ Reduced costs. Figure 3 shows the summary of web browser parameters.

##### Cross-plateform compatibility

Most web browsers are far more compatible across platforms than traditional installed software. Typically the minimum requirement would be a web browser of which there are many. (Internet Explorer, Firefox, Netscape to name but a few). These web browsers are available for a multitude of operating systems and so whether you use Windows, Linux or Mac OS you can still run the web application.

##### More manageable

For web-based systems, the end user workstation needs very little installation beyond the server. Maintaining and updating the system is made much easier because everything can usually be done on the server. Any client updates can be easily deployed via the web server.

##### Highly deployable

Because of their cross-platform compatibility and manageability, web applications are much easier to deploy to end users. They work well when there is a lack of bandwidth and the user is far from the system and data. Sending the user a website address to log in and enable Internet access is the easiest way to implement them. By giving more of your customers, suppliers, and third parties access to your systems, you can expand access to your systems, streamline processes, and enhance relationships.

##### Secure live data

Data is typically stored in separate systems and sources and moved among larger, more complex systems. It is frequently possible to consolidate these systems and processes in web-based systems, which eliminates the need to move data around. By preventing the user from having access to the data and back-end servers, web browsers additionally add a layer of security.

##### Reduced costs

Due to their lower support and maintenance needs, fewer system requirements for end users, and simplified architecture, web browsers can significantly reduce costs. Additional savings are frequently found by streamlining your company’s operations as a result of your web browser.

In order to form a well-rounded opinion, the expert carefully considers all pertinent factors during a thorough analysis of a web browser. In this process, the expert’s observations $\left(F, {\acute{\text{\AA}}}\right)$ become vital, offering important insights into how well the browser performs across a range of parameters. The expert then assigns values for membership, abstention, and non-membership within the given framework after delving into the details of these parameters.

The judgment of the specialist includes creating matrices that indicate membership, non-membership, and abstinence for each unique group. These matrices are fundamental tools that provide a structured framework for the expert’s nuanced evaluations. Using these matrices, a methodical and quantitative approach is presented, which allows for a more accurate comprehension of the web browser’s compliance with the established standards. This careful inspection guarantees an extensive assessment that surpasses superficial factors, promoting a more profound comprehension of the browser’s appropriateness concerning a variety of standards and viewpoints, for $n=2$ n.

$$\left(F,{\acute{\text{\AA}}}\right)=\left(\begin{array}{c}\left(\left(\begin{array}{c}0.48{e}^{(0.31)\pi {{\it i}}},\\ 0.46{e}^{(0.23)\pi {{\it i}}},\\ 0.40{e}^{\left(0.39\right)\pi {{\it i}}}\end{array}\right),\left(\begin{array}{c}0.49{e}^{\left(0.23\right)\pi {{\it i}}},\\ 0.54{e}^{\left(0.39\right)\pi {{\it i}}},\\ 0.40{e}^{\left(0.45\right)\pi {{\it i}}}\end{array}\right),\left(\begin{array}{c}0.46{e}^{\left(0.34\right)\pi {{\it i}}},\\ 0.35{e}^{\left(0.46\right)\pi {{\it i}}},\\ 0.38{e}^{\left(0.40\right)\pi {{\it i}}}\end{array}\right),\left(\begin{array}{c}0.49{e}^{\left(0.36\right)\pi {{\it i}}},\\ 0.43{e}^{\left(0.41\right)\pi {{\it i}}},\\ 0.43{e}^{\left(0.48\right)\pi {{\it i}}}\end{array}\right),\left(\begin{array}{c}0.44{e}^{\left(0.35\right)\pi {{\it i}}},\\ 0.39{e}^{\left(0.43\right)\pi {{\it i}}},\\ 0.40{e}^{\left(0.34\right)\pi {{\it i}}}\end{array}\right),\left(\begin{array}{c}0.44{e}^{\left(0.39\right)\pi {{\it i}}},\\ 0.42{e}^{\left(0.43\right)\pi {{\it i}}},\\ 0.43{e}^{\left(0.43\right)\pi {{\it i}}}\end{array}\right)\right)\\ \left(\left(\begin{array}{c}0.44{e}^{\left(0.29\right)\pi {{\it i}}},\\ 0.35{e}^{\left(0.30\right)\pi {{\it i}}},\\ 0.34{e}^{\left(0.40\right)\pi {{\it i}}}\end{array}\right),\left(\begin{array}{c}0.48{e}^{\left(0.41\right)\pi {{\it i}}},\\ 0.41{e}^{\left(0.40\right)\pi {{\it i}}},\\ 0.39{e}^{\left(0.45\right)\pi {{\it i}}}\end{array}\right),\left(\begin{array}{c}0.52{e}^{\left(0.41\right)\pi {{\it i}}},\\ 0.53{e}^{\left(0.33\right)\pi {{\it i}}},\\ 0.51{e}^{\left(0.48\right)\pi {{\it i}}}\end{array}\right),\left(\begin{array}{c}0.51{e}^{\left(0.30\right)\pi {{\it i}}},\\ 0.35{e}^{\left(0.29\right)\pi {{\it i}}},\\ 0.43{e}^{\left(0.49\right)\pi {{\it i}}}\end{array}\right),\left(\begin{array}{c}0.41{e}^{\left(0.31\right)\pi {{\it i}}},\\ 0.30{e}^{\left(0.49\right)\pi {{\it i}}},\\ 0.37{e}^{\left(0.41\right)\pi {{\it i}}}\end{array}\right),\left(\begin{array}{c}0.49{e}^{\left(0.36\right)\pi {{\it i}}},\\ 0.43{e}^{\left(0.41\right)\pi {{\it i}}},\\ 0.43{e}^{\left(0.48\right)\pi {{\it i}}}\end{array}\right)\right)\\ \left(\left(\begin{array}{c}0.55{e}^{(0.34)\pi {{\it i}}},\\ 0.45{e}^{(0.39)\pi {{\it i}}},\\ 0.37{e}^{\left(0.40\right)\pi {{\it i}}}\end{array}\right),\left(\begin{array}{c}0.51{e}^{\left(0.40\right)\pi {{\it i}}},\\ 0.49{e}^{\left(0.33\right)\pi {{\it i}}},\\ 0.41{e}^{\left(0.40\right)\pi {{\it i}}}\end{array}\right),\left(\begin{array}{c}0.53{e}^{\left(0.41\right)\pi {{\it i}}},\\ 0.42{e}^{\left(0.39\right)\pi {{\it i}}},\\ 0.38{e}^{\left(0.39\right)\pi {{\it i}}}\end{array}\right),\left(\begin{array}{c}0.54{e}^{\left(0.41\right)\pi {{\it i}}},\\ 0.35{e}^{\left(0.41\right)\pi {{\it i}}},\\ 0.45{e}^{\left(0.51\right)\pi {{\it i}}}\end{array}\right),\left(\begin{array}{c}0.42{e}^{\left(0.37\right)\pi {{\it i}}},\\ 0.37{e}^{\left(0.43\right)\pi {{\it i}}},\\ 0.36{e}^{\left(0.42\right)\pi {{\it i}}}\end{array}\right),\left(\begin{array}{c}0.47{e}^{\left(0.40\right)\pi {{\it i}}},\\ 0.45{e}^{\left(0.43\right)\pi {{\it i}}},\\ 0.44{e}^{\left(0.47\right)\pi {{\it i}}}\end{array}\right)\right)\\ \left(\left(\begin{array}{c}0.48{e}^{\left(0.31\right)\pi {{\it i}}},\\ 0.49{e}^{\left(0.23\right)\pi {{\it i}}},\\ 0.37{e}^{\left(0.40\right)\pi {{\it i}}}\end{array}\right),\left(\begin{array}{c}0.49{e}^{\left(0.23\right)\pi {{\it i}}},\\ 0.49{e}^{\left(0.38\right)\pi {{\it i}}},\\ 0.41{e}^{\left(0.40\right)\pi {{\it i}}}\end{array}\right),\left(\begin{array}{c}0.46{e}^{\left(0.34\right)\pi {{\it i}}},\\ 0.35{e}^{\left(0.39\right)\pi {{\it i}}},\\ 0.38{e}^{\left(0.39\right)\pi {{\it i}}}\end{array}\right),\left(\begin{array}{c}0.49{e}^{\left(0.36\right)\pi {{\it i}}},\\ 0.35{e}^{\left(0.47\right)\pi {{\it i}}},\\ 0.45{e}^{\left(0.51\right)\pi {{\it i}}}\end{array}\right),\left(\begin{array}{c}0.48{e}^{\left(0.37\right)\pi {{\it i}}},\\ 0.38{e}^{\left(0.43\right)\pi {{\it i}}},\\ 0.40{e}^{\left(0.45\right)\pi {{\it i}}}\end{array}\right),\left(\begin{array}{c}0.46{e}^{\left(0.38\right)\pi {{\it i}}},\\ 0.48{e}^{\left(0.44\right)\pi {{\it i}}},\\ 0.42{e}^{\left(0.47\right)\pi {{\it i}}}\end{array}\right)\right)\\ \left(\left(\begin{array}{c}0.55{e}^{(0.34)\pi {{\it i}}},\\ 0.45{e}^{(0.39)\pi {{\it i}}},\\ 0.40{e}^{\left(0.40\right)\pi {{\it i}}}\end{array}\right),\left(\begin{array}{c}0.52{e}^{\left(0.39\right)\pi {{\it i}}},\\ 0.39{e}^{\left(0.33\right)\pi {{\it i}}},\\ 0.41{e}^{\left(0.45\right)\pi {{\it i}}}\end{array}\right),\left(\begin{array}{c}0.53{e}^{\left(0.45\right)\pi {{\it i}}},\\ 0.41{e}^{\left(0.46\right)\pi {{\it i}}},\\ 0.39{e}^{\left(0.40\right)\pi {{\it i}}}\end{array}\right),\left(\begin{array}{c}0.54{e}^{\left(0.46\right)\pi {{\it i}}},\\ 0.51{e}^{\left(0.41\right)\pi {{\it i}}},\\ 0.43{e}^{\left(0.46\right)\pi {{\it i}}}\end{array}\right),\left(\begin{array}{c}0.49{e}^{\left(0.31\right)\pi {{\it i}}},\\ 0.37{e}^{\left(0.43\right)\pi {{\it i}}},\\ 0.39{e}^{\left(0.39\right)\pi {{\it i}}}\end{array}\right),\left(\begin{array}{c}0.51{e}^{\left(0.30\right)\pi {{\it i}}},\\ 0.42{e}^{\left(0.41\right)\pi {{\it i}}},\\ 0.43{e}^{\left(0.48\right)\pi {{\it i}}}\end{array}\right)\right)\end{array}\right).$$

Suppose that the first five values of matrices ${\check{\mathrm{u}}}_{1},{\check{\mathrm{u}}}_{2},{\check{\mathrm{u}}}_{3},{\check{\mathrm{u}}}_{4}$ and ${\check{\mathrm{u}}}_{5}$ of each parameter give the values of membership, abstinence, and non-membership by expert. The sixth value of $\lambda$ of each parameter gives the value of membership, abstinence, and non-membership by expert and the sixth value are indicating the general belongingness value to the web browser. Then, its self-CP of $\left(F,{\acute{\text{\AA}}}\right)$ is shown in Table 2.

**Table 2:** CP of web browser.

Ordered pair	${\check{\mathrm{u}}}_{1}$	${\check{\mathrm{u}}}_{2}$	${\check{\mathrm{u}}}_{3}$	${\check{\mathrm{u}}}_{4}$	${\check{\mathrm{u}}}_{5}$	$\lambda$
($${s}_{1}$$,$${s}_{1})$$	$$\left(\begin{array}{c}0.48{e}^{(0.31)\pi {{\it i}}},\\ 0.46{e}^{(0.23)\pi {{\it i}}},\\ 0.40{e}^{\left(0.39\right)\pi {{\it i}}}\end{array}\right)$$	$$\left(\begin{array}{c}0.49{e}^{\left(0.23\right)\pi {{\it i}}},\\ 0.54{e}^{\left(0.39\right)\pi {{\it i}}},\\ 0.40{e}^{\left(0.45\right)\pi {{\it i}}}\end{array}\right)$$	$$\left(\begin{array}{c}0.46{e}^{\left(0.34\right)\pi {{\it i}}},\\ 0.35{e}^{\left(0.46\right)\pi {{\it i}}},\\ 0.38{e}^{\left(0.40\right)\pi {{\it i}}}\end{array}\right)$$	$$\left(\begin{array}{c}0.49{e}^{\left(0.36\right)\pi {{\it i}}},\\ 0.43{e}^{\left(0.41\right)\pi {{\it i}}},\\ 0.43{e}^{\left(0.48\right)\pi {{\it i}}}\end{array}\right)$$	$$\left(\begin{array}{c}0.44{e}^{\left(0.35\right)\pi {{\it i}}},\\ 0.39{e}^{\left(0.43\right)\pi {{\it i}}},\\ 0.40{e}^{\left(0.34\right)\pi {{\it i}}}\end{array}\right)$$	$$\left(\begin{array}{c}0.44{e}^{\left(0.39\right)\pi {{\it i}}},\\ 0.42{e}^{\left(0.43\right)\pi {{\it i}}},\\ 0.43{e}^{\left(0.43\right)\pi {{\it i}}}\end{array}\right)$$
($${s}_{1}$$,$${s}_{2})$$	$$\left(\begin{array}{c}0.44{e}^{(0.29)\pi {{\it i}}},\\ 0.35{e}^{(0.23)\pi {{\it i}}},\\ 0.40{e}^{\left(0.39\right)\pi {{\it i}}}\end{array}\right)$$	$$\left(\begin{array}{c}0.48{e}^{\left(0.23\right)\pi {{\it i}}},\\ 0.41{e}^{\left(0.39\right)\pi {{\it i}}},\\ 0.40{e}^{\left(0.45\right)\pi {{\it i}}}\end{array}\right)$$	$$\left(\begin{array}{c}0.46{e}^{\left(0.34\right)\pi {{\it i}}},\\ 0.35{e}^{\left(0.33\right)\pi {{\it i}}},\\ 0.51{e}^{\left(0.48\right)\pi {{\it i}}}\end{array}\right)$$	$$\left(\begin{array}{c}0.49{e}^{\left(0.30\right)\pi {{\it i}}},\\ 0.35{e}^{\left(0.29\right)\pi {{\it i}}},\\ 0.43{e}^{\left(0.49\right)\pi {{\it i}}}\end{array}\right)$$	$$\left(\begin{array}{c}0.41{e}^{\left(0.31\right)\pi {{\it i}}},\\ 0.30{e}^{\left(0.43\right)\pi {{\it i}}},\\ 0.40{e}^{\left(0.41\right)\pi {{\it i}}}\end{array}\right)$$	$$\left(\begin{array}{c}0.44{e}^{\left(0.36\right)\pi {{\it i}}},\\ 0.42{e}^{\left(0.41\right)\pi {{\it i}}},\\ 0.43{e}^{\left(0.48\right)\pi {{\it i}}}\end{array}\right)$$
($${s}_{1}$$,$${s}_{3})$$	$$\left(\begin{array}{c}0.48{e}^{(0.31)\pi {{\it i}}},\\ 0.45{e}^{(0.23)\pi {{\it i}}},\\ 0.40{e}^{\left(0.40\right)\pi {{\it i}}}\end{array}\right)$$	$$\left(\begin{array}{c}0.49{e}^{\left(0.23\right)\pi {{\it i}}},\\ 0.49{e}^{\left(0.33\right)\pi {{\it i}}},\\ 0.41{e}^{\left(0.45\right)\pi {{\it i}}}\end{array}\right)$$	$$\left(\begin{array}{c}0.46{e}^{\left(0.34\right)\pi {{\it i}}},\\ 0.35{e}^{\left(0.39\right)\pi {{\it i}}},\\ 0.38{e}^{\left(0.40\right)\pi {{\it i}}}\end{array}\right)$$	$$\left(\begin{array}{c}0.49{e}^{\left(0.36\right)\pi {{\it i}}},\\ 0.35{e}^{\left(0.41\right)\pi {{\it i}}},\\ 0.45{e}^{\left(0.51\right)\pi {{\it i}}}\end{array}\right)$$	$$\left(\begin{array}{c}0.42{e}^{\left(0.35\right)\pi {{\it i}}},\\ 0.37{e}^{\left(0.43\right)\pi {{\it i}}},\\ 0.40{e}^{\left(0.42\right)\pi {{\it i}}}\end{array}\right)$$	$$\left(\begin{array}{c}0.44{e}^{\left(0.39\right)\pi {{\it i}}},\\ 0.42{e}^{\left(0.43\right)\pi {{\it i}}},\\ 0.44{e}^{\left(0.47\right)\pi {{\it i}}}\end{array}\right)$$
($${s}_{1}$$,$${s}_{4})$$	$$\left(\begin{array}{c}0.48{e}^{(0.31)\pi {{\it i}}},\\ 0.46{e}^{(0.23)\pi {{\it i}}},\\ 0.40{e}^{\left(0.40\right)\pi {{\it i}}}\end{array}\right)$$	$$\left(\begin{array}{c}0.49{e}^{\left(0.23\right)\pi {{\it i}}},\\ 0.49{e}^{\left(0.38\right)\pi {{\it i}}},\\ 0.41{e}^{\left(0.45\right)\pi {{\it i}}}\end{array}\right)$$	$$\left(\begin{array}{c}0.46{e}^{\left(0.34\right)\pi {{\it i}}},\\ 0.35{e}^{\left(0.39\right)\pi {{\it i}}},\\ 0.38{e}^{\left(0.40\right)\pi {{\it i}}}\end{array}\right)$$	$$\left(\begin{array}{c}0.49{e}^{\left(0.36\right)\pi {{\it i}}},\\ 0.35{e}^{\left(0.41\right)\pi {{\it i}}},\\ 0.45{e}^{\left(0.51\right)\pi {{\it i}}}\end{array}\right)$$	$$\left(\begin{array}{c}0.44{e}^{\left(0.35\right)\pi {{\it i}}},\\ 0.38{e}^{\left(0.43\right)\pi {{\it i}}},\\ 0.40{e}^{\left(0.45\right)\pi {{\it i}}}\end{array}\right)$$	$$\left(\begin{array}{c}0.44{e}^{\left(0.38\right)\pi {{\it i}}},\\ 0.42{e}^{\left(0.43\right)\pi {{\it i}}},\\ 0.43{e}^{\left(0.47\right)\pi {{\it i}}}\end{array}\right)$$
($${s}_{1}$$,$${s}_{5})$$	$$\left(\begin{array}{c}0.48{e}^{(0.31)\pi {{\it i}}},\\ 0.45{e}^{(0.23)\pi {{\it i}}},\\ 0.40{e}^{\left(0.40\right)\pi {{\it i}}}\end{array}\right)$$	$$\left(\begin{array}{c}0.49{e}^{\left(0.23\right)\pi {{\it i}}},\\ 0.39{e}^{\left(0.33\right)\pi {{\it i}}},\\ 0.41{e}^{\left(0.45\right)\pi {{\it i}}}\end{array}\right)$$	$$\left(\begin{array}{c}0.46{e}^{\left(0.34\right)\pi {{\it i}}},\\ 0.35{e}^{\left(0.46\right)\pi {{\it i}}},\\ 0.39{e}^{\left(0.40\right)\pi {{\it i}}}\end{array}\right)$$	$$\left(\begin{array}{c}0.49{e}^{\left(0.36\right)\pi {{\it i}}},\\ 0.43{e}^{\left(0.41\right)\pi {{\it i}}},\\ 0.43{e}^{\left(0.48\right)\pi {{\it i}}}\end{array}\right)$$	$$\left(\begin{array}{c}0.44{e}^{\left(0.31\right)\pi {{\it i}}},\\ 0.37{e}^{\left(0.43\right)\pi {{\it i}}},\\ 0.40{e}^{\left(0.39\right)\pi {{\it i}}}\end{array}\right)$$	$$\left(\begin{array}{c}0.44{e}^{\left(0.30\right)\pi {{\it i}}},\\ 0.42{e}^{\left(0.41\right)\pi {{\it i}}},\\ 0.43{e}^{\left(0.48\right)\pi {{\it i}}}\end{array}\right)$$
($${s}_{2}$$,$${s}_{1})$$	$$\left(\begin{array}{c}0.44{e}^{(0.29)\pi {{\it i}}},\\ 0.35{e}^{(0.23)\pi {{\it i}}},\\ 0.40{e}^{\left(0.39\right)\pi {{\it i}}}\end{array}\right)$$	$$\left(\begin{array}{c}0.48{e}^{\left(0.23\right)\pi {{\it i}}},\\ 0.41{e}^{\left(0.39\right)\pi {{\it i}}},\\ 0.40{e}^{\left(0.45\right)\pi {{\it i}}}\end{array}\right)$$	$$\left(\begin{array}{c}0.46{e}^{\left(0.34\right)\pi {{\it i}}},\\ 0.35{e}^{\left(0.33\right)\pi {{\it i}}},\\ 0.51{e}^{\left(0.48\right)\pi {{\it i}}}\end{array}\right)$$	$$\left(\begin{array}{c}0.49{e}^{\left(0.30\right)\pi {{\it i}}},\\ 0.35{e}^{\left(0.29\right)\pi {{\it i}}},\\ 0.43{e}^{\left(0.49\right)\pi {{\it i}}}\end{array}\right)$$	$$\left(\begin{array}{c}0.41{e}^{\left(0.31\right)\pi {{\it i}}},\\ 0.30{e}^{\left(0.43\right)\pi {{\it i}}},\\ 0.40{e}^{\left(0.41\right)\pi {{\it i}}}\end{array}\right)$$	$$\left(\begin{array}{c}0.44{e}^{\left(0.36\right)\pi {{\it i}}},\\ 0.42{e}^{\left(0.41\right)\pi {{\it i}}},\\ 0.43{e}^{\left(0.48\right)\pi {{\it i}}}\end{array}\right)$$
($${s}_{2}$$,$${s}_{2})$$	$$\left(\begin{array}{c}0.44{e}^{\left(0.29\right)\pi {{\it i}}},\\ 0.35{e}^{\left(0.30\right)\pi {{\it i}}},\\ 0.34{e}^{\left(0.40\right)\pi {{\it i}}}\end{array}\right)$$	$$\left(\begin{array}{c}0.48{e}^{\left(0.41\right)\pi {{\it i}}},\\ 0.41{e}^{\left(0.40\right)\pi {{\it i}}},\\ 0.39{e}^{\left(0.45\right)\pi {{\it i}}}\end{array}\right)$$	$$\left(\begin{array}{c}0.52{e}^{\left(0.41\right)\pi {{\it i}}},\\ 0.53{e}^{\left(0.33\right)\pi {{\it i}}},\\ 0.51{e}^{\left(0.48\right)\pi {{\it i}}}\end{array}\right)$$	$$\left(\begin{array}{c}0.51{e}^{\left(0.30\right)\pi {{\it i}}},\\ 0.35{e}^{\left(0.29\right)\pi {{\it i}}},\\ 0.43{e}^{\left(0.49\right)\pi {{\it i}}}\end{array}\right)$$	$$\left(\begin{array}{c}0.41{e}^{\left(0.31\right)\pi {{\it i}}},\\ 0.30{e}^{\left(0.49\right)\pi {{\it i}}},\\ 0.37{e}^{\left(0.41\right)\pi {{\it i}}}\end{array}\right)$$	$$\left(\begin{array}{c}0.49{e}^{\left(0.36\right)\pi {{\it i}}},\\ 0.43{e}^{\left(0.41\right)\pi {{\it i}}},\\ 0.43{e}^{\left(0.48\right)\pi {{\it i}}}\end{array}\right)$$
($${s}_{2}$$,$${s}_{3})$$	$$\left(\begin{array}{c}0.44{e}^{\left(0.29\right)\pi {{\it i}}},\\ 0.35{e}^{\left(0.30\right)\pi {{\it i}}},\\ 0.37{e}^{\left(0.40\right)\pi {{\it i}}}\end{array}\right)$$	$$\left(\begin{array}{c}0.48{e}^{\left(0.40\right)\pi {{\it i}}},\\ 0.41{e}^{\left(0.33\right)\pi {{\it i}}},\\ 0.41{e}^{\left(0.45\right)\pi {{\it i}}}\end{array}\right)$$	$$\left(\begin{array}{c}0.52{e}^{\left(0.41\right)\pi {{\it i}}},\\ 0.42{e}^{\left(0.33\right)\pi {{\it i}}},\\ 0.51{e}^{\left(0.48\right)\pi {{\it i}}}\end{array}\right)$$	$$\left(\begin{array}{c}0.51{e}^{\left(0.30\right)\pi {{\it i}}},\\ 0.35{e}^{\left(0.29\right)\pi {{\it i}}},\\ 0.45{e}^{\left(0.51\right)\pi {{\it i}}}\end{array}\right)$$	$$\left(\begin{array}{c}0.41{e}^{\left(0.31\right)\pi {{\it i}}},\\ 0.30{e}^{\left(0.43\right)\pi {{\it i}}},\\ 0.37{e}^{\left(0.42\right)\pi {{\it i}}}\end{array}\right)$$	$$\left(\begin{array}{c}0.47{e}^{\left(0.36\right)\pi {{\it i}}},\\ 0.43{e}^{\left(0.41\right)\pi {{\it i}}},\\ 0.44{e}^{\left(0.48\right)\pi {{\it i}}}\end{array}\right)$$
($${s}_{2}$$,$${s}_{4})$$	$$\left(\begin{array}{c}0.44{e}^{\left(0.29\right)\pi {{\it i}}},\\ 0.35{e}^{\left(0.23\right)\pi {{\it i}}},\\ 0.37{e}^{\left(0.40\right)\pi {{\it i}}}\end{array}\right)$$	$$\left(\begin{array}{c}0.48{e}^{\left(0.23\right)\pi {{\it i}}},\\ 0.41{e}^{\left(0.38\right)\pi {{\it i}}},\\ 0.41{e}^{\left(0.45\right)\pi {{\it i}}}\end{array}\right)$$	$$\left(\begin{array}{c}0.46{e}^{\left(0.34\right)\pi {{\it i}}},\\ 0.35{e}^{\left(0.33\right)\pi {{\it i}}},\\ 0.51{e}^{\left(0.48\right)\pi {{\it i}}}\end{array}\right)$$	$$\left(\begin{array}{c}0.49{e}^{\left(0.30\right)\pi {{\it i}}},\\ 0.35{e}^{\left(0.29\right)\pi {{\it i}}},\\ 0.45{e}^{\left(0.51\right)\pi {{\it i}}}\end{array}\right)$$	$$\left(\begin{array}{c}0.41{e}^{\left(0.31\right)\pi {{\it i}}},\\ 0.30{e}^{\left(0.43\right)\pi {{\it i}}},\\ 0.40{e}^{\left(0.45\right)\pi {{\it i}}}\end{array}\right)$$	$$\left(\begin{array}{c}0.46{e}^{\left(0.36\right)\pi {{\it i}}},\\ 0.43{e}^{\left(0.41\right)\pi {{\it i}}},\\ 0.43{e}^{\left(0.48\right)\pi {{\it i}}}\end{array}\right)$$
($${s}_{2}$$,$${s}_{5})$$	$$\left(\begin{array}{c}0.44{e}^{\left(0.29\right)\pi {{\it i}}},\\ 0.35{e}^{\left(0.30\right)\pi {{\it i}}},\\ 0.40{e}^{\left(0.40\right)\pi {{\it i}}}\end{array}\right)$$	$$\left(\begin{array}{c}0.48{e}^{\left(0.39\right)\pi {{\it i}}},\\ 0.39{e}^{\left(0.33\right)\pi {{\it i}}},\\ 0.41{e}^{\left(0.45\right)\pi {{\it i}}}\end{array}\right)$$	$$\left(\begin{array}{c}0.52{e}^{\left(0.41\right)\pi {{\it i}}},\\ 0.41{e}^{\left(0.33\right)\pi {{\it i}}},\\ 0.51{e}^{\left(0.48\right)\pi {{\it i}}}\end{array}\right)$$	$$\left(\begin{array}{c}0.51{e}^{\left(0.30\right)\pi {{\it i}}},\\ 0.35{e}^{\left(0.29\right)\pi {{\it i}}},\\ 0.43{e}^{\left(0.49\right)\pi {{\it i}}}\end{array}\right)$$	$$\left(\begin{array}{c}0.41{e}^{\left(0.31\right)\pi {{\it i}}},\\ 0.30{e}^{\left(0.43\right)\pi {{\it i}}},\\ 0.39{e}^{\left(0.41\right)\pi {{\it i}}}\end{array}\right)$$	$$\left(\begin{array}{c}0.49{e}^{\left(0.30\right)\pi {{\it i}}},\\ 0.42{e}^{\left(0.41\right)\pi {{\it i}}},\\ 0.43{e}^{\left(0.48\right)\pi {{\it i}}}\end{array}\right)$$
($${s}_{3}$$,$${s}_{1})$$	$$\left(\begin{array}{c}0.48{e}^{(0.31)\pi {{\it i}}},\\ 0.45{e}^{(0.23)\pi {{\it i}}},\\ 0.40{e}^{\left(0.40\right)\pi {{\it i}}}\end{array}\right)$$	$$\left(\begin{array}{c}0.49{e}^{\left(0.23\right)\pi {{\it i}}},\\ 0.49{e}^{\left(0.33\right)\pi {{\it i}}},\\ 0.41{e}^{\left(0.45\right)\pi {{\it i}}}\end{array}\right)$$	$$\left(\begin{array}{c}0.46{e}^{\left(0.34\right)\pi {{\it i}}},\\ 0.35{e}^{\left(0.39\right)\pi {{\it i}}},\\ 0.38{e}^{\left(0.40\right)\pi {{\it i}}}\end{array}\right)$$	$$\left(\begin{array}{c}0.49{e}^{\left(0.36\right)\pi {{\it i}}},\\ 0.35{e}^{\left(0.41\right)\pi {{\it i}}},\\ 0.45{e}^{\left(0.51\right)\pi {{\it i}}}\end{array}\right)$$	$$\left(\begin{array}{c}0.42{e}^{\left(0.35\right)\pi {{\it i}}},\\ 0.37{e}^{\left(0.43\right)\pi {{\it i}}},\\ 0.40{e}^{\left(0.42\right)\pi {{\it i}}}\end{array}\right)$$	$$\left(\begin{array}{c}0.44{e}^{\left(0.39\right)\pi {{\it i}}},\\ 0.42{e}^{\left(0.43\right)\pi {{\it i}}},\\ 0.44{e}^{\left(0.47\right)\pi {{\it i}}}\end{array}\right)$$
($${s}_{3}$$,$${s}_{2})$$	$$\left(\begin{array}{c}0.44{e}^{\left(0.29\right)\pi {{\it i}}},\\ 0.35{e}^{\left(0.30\right)\pi {{\it i}}},\\ 0.37{e}^{\left(0.40\right)\pi {{\it i}}}\end{array}\right)$$	$$\left(\begin{array}{c}0.48{e}^{\left(0.40\right)\pi {{\it i}}},\\ 0.41{e}^{\left(0.33\right)\pi {{\it i}}},\\ 0.41{e}^{\left(0.45\right)\pi {{\it i}}}\end{array}\right)$$	$$\left(\begin{array}{c}0.52{e}^{\left(0.41\right)\pi {{\it i}}},\\ 0.42{e}^{\left(0.33\right)\pi {{\it i}}},\\ 0.51{e}^{\left(0.48\right)\pi {{\it i}}}\end{array}\right)$$	$$\left(\begin{array}{c}0.51{e}^{\left(0.30\right)\pi {{\it i}}},\\ 0.35{e}^{\left(0.29\right)\pi {{\it i}}},\\ 0.45{e}^{\left(0.51\right)\pi {{\it i}}}\end{array}\right)$$	$$\left(\begin{array}{c}0.41{e}^{\left(0.31\right)\pi {{\it i}}},\\ 0.30{e}^{\left(0.43\right)\pi {{\it i}}},\\ 0.37{e}^{\left(0.42\right)\pi {{\it i}}}\end{array}\right)$$	$$\left(\begin{array}{c}0.47{e}^{\left(0.36\right)\pi {{\it i}}},\\ 0.43{e}^{\left(0.41\right)\pi {{\it i}}},\\ 0.44{e}^{\left(0.48\right)\pi {{\it i}}}\end{array}\right)$$
($${s}_{3}$$,$${s}_{3})$$	$$\left(\begin{array}{c}0.55{e}^{(0.34)\pi {{\it i}}},\\ 0.45{e}^{(0.39)\pi {{\it i}}},\\ 0.37{e}^{\left(0.40\right)\pi {{\it i}}}\end{array}\right)$$	$$\left(\begin{array}{c}0.51{e}^{\left(0.40\right)\pi {{\it i}}},\\ 0.49{e}^{\left(0.33\right)\pi {{\it i}}},\\ 0.41{e}^{\left(0.40\right)\pi {{\it i}}}\end{array}\right)$$	$$\left(\begin{array}{c}0.53{e}^{\left(0.41\right)\pi {{\it i}}},\\ 0.42{e}^{\left(0.39\right)\pi {{\it i}}},\\ 0.38{e}^{\left(0.39\right)\pi {{\it i}}}\end{array}\right)$$	$$\left(\begin{array}{c}0.54{e}^{\left(0.41\right)\pi {{\it i}}},\\ 0.35{e}^{\left(0.41\right)\pi {{\it i}}},\\ 0.45{e}^{\left(0.51\right)\pi {{\it i}}}\end{array}\right)$$	$$\left(\begin{array}{c}0.42{e}^{\left(0.37\right)\pi {{\it i}}},\\ 0.37{e}^{\left(0.43\right)\pi {{\it i}}},\\ 0.36{e}^{\left(0.42\right)\pi {{\it i}}}\end{array}\right)$$	$$\left(\begin{array}{c}0.47{e}^{\left(0.40\right)\pi {{\it i}}},\\ 0.45{e}^{\left(0.43\right)\pi {{\it i}}},\\ 0.44{e}^{\left(0.47\right)\pi {{\it i}}}\end{array}\right)$$
($${s}_{3}$$,$${s}_{4})$$	$$\left(\begin{array}{c}0.48{e}^{(0.31)\pi {{\it i}}},\\ 0.45{e}^{(0.23)\pi {{\it i}}},\\ 0.37{e}^{\left(0.40\right)\pi {{\it i}}}\end{array}\right)$$	$$\left(\begin{array}{c}0.49{e}^{\left(0.23\right)\pi {{\it i}}},\\ 0.49{e}^{\left(0.33\right)\pi {{\it i}}},\\ 0.41{e}^{\left(0.40\right)\pi {{\it i}}}\end{array}\right)$$	$$\left(\begin{array}{c}0.46{e}^{\left(0.34\right)\pi {{\it i}}},\\ 0.35{e}^{\left(0.39\right)\pi {{\it i}}},\\ 0.38{e}^{\left(0.39\right)\pi {{\it i}}}\end{array}\right)$$	$$\left(\begin{array}{c}0.49{e}^{\left(0.36\right)\pi {{\it i}}},\\ 0.35{e}^{\left(0.41\right)\pi {{\it i}}},\\ 0.45{e}^{\left(0.51\right)\pi {{\it i}}}\end{array}\right)$$	$$\left(\begin{array}{c}0.42{e}^{\left(0.37\right)\pi {{\it i}}},\\ 0.37{e}^{\left(0.43\right)\pi {{\it i}}},\\ 0.40{e}^{\left(0.45\right)\pi {{\it i}}}\end{array}\right)$$	$$\left(\begin{array}{c}0.47{e}^{\left(0.30\right)\pi {{\it i}}},\\ 0.42{e}^{\left(0.41\right)\pi {{\it i}}},\\ 0.44{e}^{\left(0.48\right)\pi {{\it i}}}\end{array}\right)$$
($${s}_{3}$$,$${s}_{5})$$	$$\left(\begin{array}{c}0.55{e}^{(0.34)\pi {{\it i}}},\\ 0.45{e}^{(0.39)\pi {{\it i}}},\\ 0.40{e}^{\left(0.40\right)\pi {{\it i}}}\end{array}\right)$$	$$\left(\begin{array}{c}0.51{e}^{\left(0.39\right)\pi {{\it i}}},\\ 0.39{e}^{\left(0.33\right)\pi {{\it i}}},\\ 0.41{e}^{\left(0.45\right)\pi {{\it i}}}\end{array}\right)$$	$$\left(\begin{array}{c}0.53{e}^{\left(0.41\right)\pi {{\it i}}},\\ 0.41{e}^{\left(0.39\right)\pi {{\it i}}},\\ 0.39{e}^{\left(0.40\right)\pi {{\it i}}}\end{array}\right)$$	$$\left(\begin{array}{c}0.54{e}^{\left(0.41\right)\pi {{\it i}}},\\ 0.35{e}^{\left(0.41\right)\pi {{\it i}}},\\ 0.45{e}^{\left(0.51\right)\pi {{\it i}}}\end{array}\right)$$	$$\left(\begin{array}{c}0.42{e}^{\left(0.31\right)\pi {{\it i}}},\\ 0.37{e}^{\left(0.43\right)\pi {{\it i}}},\\ 0.39{e}^{\left(0.42\right)\pi {{\it i}}}\end{array}\right)$$	$$\left(\begin{array}{c}0.47{e}^{\left(0.30\right)\pi {{\it i}}},\\ 0.42{e}^{\left(0.41\right)\pi {{\it i}}},\\ 0.44{e}^{\left(0.48\right)\pi {{\it i}}}\end{array}\right)$$
($${s}_{4}$$,$${s}_{1})$$	$$\left(\begin{array}{c}0.48{e}^{(0.31)\pi {{\it i}}},\\ 0.46{e}^{(0.23)\pi {{\it i}}},\\ 0.40{e}^{\left(0.40\right)\pi {{\it i}}}\end{array}\right)$$	$$\left(\begin{array}{c}0.49{e}^{\left(0.23\right)\pi {{\it i}}},\\ 0.49{e}^{\left(0.38\right)\pi {{\it i}}},\\ 0.41{e}^{\left(0.45\right)\pi {{\it i}}}\end{array}\right)$$	$$\left(\begin{array}{c}0.46{e}^{\left(0.34\right)\pi {{\it i}}},\\ 0.35{e}^{\left(0.39\right)\pi {{\it i}}},\\ 0.38{e}^{\left(0.40\right)\pi {{\it i}}}\end{array}\right)$$	$$\left(\begin{array}{c}0.49{e}^{\left(0.36\right)\pi {{\it i}}},\\ 0.35{e}^{\left(0.41\right)\pi {{\it i}}},\\ 0.45{e}^{\left(0.51\right)\pi {{\it i}}}\end{array}\right)$$	$$\left(\begin{array}{c}0.44{e}^{\left(0.35\right)\pi {{\it i}}},\\ 0.38{e}^{\left(0.43\right)\pi {{\it i}}},\\ 0.40{e}^{\left(0.45\right)\pi {{\it i}}}\end{array}\right)$$	$$\left(\begin{array}{c}0.44{e}^{\left(0.38\right)\pi {{\it i}}},\\ 0.42{e}^{\left(0.43\right)\pi {{\it i}}},\\ 0.43{e}^{\left(0.47\right)\pi {{\it i}}}\end{array}\right)$$
($${s}_{4}$$,$${s}_{2})$$	$$\left(\begin{array}{c}0.44{e}^{\left(0.29\right)\pi {{\it i}}},\\ 0.35{e}^{\left(0.23\right)\pi {{\it i}}},\\ 0.37{e}^{\left(0.40\right)\pi {{\it i}}}\end{array}\right)$$	$$\left(\begin{array}{c}0.48{e}^{\left(0.23\right)\pi {{\it i}}},\\ 0.41{e}^{\left(0.38\right)\pi {{\it i}}},\\ 0.41{e}^{\left(0.45\right)\pi {{\it i}}}\end{array}\right)$$	$$\left(\begin{array}{c}0.46{e}^{\left(0.34\right)\pi {{\it i}}},\\ 0.35{e}^{\left(0.33\right)\pi {{\it i}}},\\ 0.51{e}^{\left(0.48\right)\pi {{\it i}}}\end{array}\right)$$	$$\left(\begin{array}{c}0.49{e}^{\left(0.30\right)\pi {{\it i}}},\\ 0.35{e}^{\left(0.29\right)\pi {{\it i}}},\\ 0.45{e}^{\left(0.51\right)\pi {{\it i}}}\end{array}\right)$$	$$\left(\begin{array}{c}0.41{e}^{\left(0.31\right)\pi {{\it i}}},\\ 0.30{e}^{\left(0.43\right)\pi {{\it i}}},\\ 0.40{e}^{\left(0.45\right)\pi {{\it i}}}\end{array}\right)$$	$$\left(\begin{array}{c}0.46{e}^{\left(0.36\right)\pi {{\it i}}},\\ 0.43{e}^{\left(0.41\right)\pi {{\it i}}},\\ 0.43{e}^{\left(0.48\right)\pi {{\it i}}}\end{array}\right)$$
($${s}_{4}$$,$${s}_{3})$$	$$\left(\begin{array}{c}0.48{e}^{(0.31)\pi {{\it i}}},\\ 0.45{e}^{(0.23)\pi {{\it i}}},\\ 0.37{e}^{\left(0.40\right)\pi {{\it i}}}\end{array}\right)$$	$$\left(\begin{array}{c}0.49{e}^{\left(0.23\right)\pi {{\it i}}},\\ 0.49{e}^{\left(0.33\right)\pi {{\it i}}},\\ 0.41{e}^{\left(0.40\right)\pi {{\it i}}}\end{array}\right)$$	$$\left(\begin{array}{c}0.46{e}^{\left(0.34\right)\pi {{\it i}}},\\ 0.35{e}^{\left(0.39\right)\pi {{\it i}}},\\ 0.38{e}^{\left(0.39\right)\pi {{\it i}}}\end{array}\right)$$	$$\left(\begin{array}{c}0.49{e}^{\left(0.36\right)\pi {{\it i}}},\\ 0.35{e}^{\left(0.41\right)\pi {{\it i}}},\\ 0.45{e}^{\left(0.51\right)\pi {{\it i}}}\end{array}\right)$$	$$\left(\begin{array}{c}0.42{e}^{\left(0.37\right)\pi {{\it i}}},\\ 0.37{e}^{\left(0.43\right)\pi {{\it i}}},\\ 0.40{e}^{\left(0.45\right)\pi {{\it i}}}\end{array}\right)$$	$$\left(\begin{array}{c}0.47{e}^{\left(0.30\right)\pi {{\it i}}},\\ 0.42{e}^{\left(0.41\right)\pi {{\it i}}},\\ 0.44{e}^{\left(0.48\right)\pi {{\it i}}}\end{array}\right)$$
($${s}_{4}$$,$${s}_{4})$$	$$\left(\begin{array}{c}0.48{e}^{\left(0.31\right)\pi {{\it i}}},\\ 0.49{e}^{\left(0.23\right)\pi {{\it i}}},\\ 0.37{e}^{\left(0.40\right)\pi {{\it i}}}\end{array}\right)$$	$$\left(\begin{array}{c}0.49{e}^{\left(0.23\right)\pi {{\it i}}},\\ 0.49{e}^{\left(0.38\right)\pi {{\it i}}},\\ 0.41{e}^{\left(0.40\right)\pi {{\it i}}}\end{array}\right)$$	$$\left(\begin{array}{c}0.46{e}^{\left(0.34\right)\pi {{\it i}}},\\ 0.35{e}^{\left(0.39\right)\pi {{\it i}}},\\ 0.38{e}^{\left(0.39\right)\pi {{\it i}}}\end{array}\right)$$	$$\left(\begin{array}{c}0.49{e}^{\left(0.36\right)\pi {{\it i}}},\\ 0.35{e}^{\left(0.47\right)\pi {{\it i}}},\\ 0.45{e}^{\left(0.51\right)\pi {{\it i}}}\end{array}\right)$$	$$\left(\begin{array}{c}0.48{e}^{\left(0.37\right)\pi {{\it i}}},\\ 0.38{e}^{\left(0.43\right)\pi {{\it i}}},\\ 0.40{e}^{\left(0.45\right)\pi {{\it i}}}\end{array}\right)$$	$$\left(\begin{array}{c}0.46{e}^{\left(0.38\right)\pi {{\it i}}},\\ 0.48{e}^{\left(0.44\right)\pi {{\it i}}},\\ 0.42{e}^{\left(0.47\right)\pi {{\it i}}}\end{array}\right)$$
($${s}_{4}$$,$${s}_{5})$$	$$\left(\begin{array}{c}0.48{e}^{\left(0.31\right)\pi {{\it i}}},\\ 0.45{e}^{\left(0.23\right)\pi {{\it i}}},\\ 0.40{e}^{\left(0.40\right)\pi {{\it i}}}\end{array}\right)$$	$$\left(\begin{array}{c}0.49{e}^{\left(0.23\right)\pi {{\it i}}},\\ 0.39{e}^{\left(0.33\right)\pi {{\it i}}},\\ 0.41{e}^{\left(0.45\right)\pi {{\it i}}}\end{array}\right)$$	$$\left(\begin{array}{c}0.46{e}^{\left(0.34\right)\pi {{\it i}}},\\ 0.35{e}^{\left(0.39\right)\pi {{\it i}}},\\ 0.39{e}^{\left(0.40\right)\pi {{\it i}}}\end{array}\right)$$	$$\left(\begin{array}{c}0.49{e}^{\left(0.36\right)\pi {{\it i}}},\\ 0.35{e}^{\left(0.41\right)\pi {{\it i}}},\\ 0.43{e}^{\left(0.46\right)\pi {{\it i}}}\end{array}\right)$$	$$\left(\begin{array}{c}0.48{e}^{\left(0.31\right)\pi {{\it i}}},\\ 0.37{e}^{\left(0.43\right)\pi {{\it i}}},\\ 0.40{e}^{\left(0.45\right)\pi {{\it i}}}\end{array}\right)$$	$$\left(\begin{array}{c}0.46{e}^{\left(0.30\right)\pi {{\it i}}},\\ 0.42{e}^{\left(0.41\right)\pi {{\it i}}},\\ 0.43{e}^{\left(0.48\right)\pi {{\it i}}}\end{array}\right)$$
($${s}_{5}$$,$${s}_{1})$$	$$\left(\begin{array}{c}0.48{e}^{(0.31)\pi {{\it i}}},\\ 0.45{e}^{(0.23)\pi {{\it i}}},\\ 0.40{e}^{\left(0.40\right)\pi {{\it i}}}\end{array}\right)$$	$$\left(\begin{array}{c}0.49{e}^{\left(0.23\right)\pi {{\it i}}},\\ 0.39{e}^{\left(0.33\right)\pi {{\it i}}},\\ 0.41{e}^{\left(0.45\right)\pi {{\it i}}}\end{array}\right)$$	$$\left(\begin{array}{c}0.46{e}^{\left(0.34\right)\pi {{\it i}}},\\ 0.35{e}^{\left(0.46\right)\pi {{\it i}}},\\ 0.39{e}^{\left(0.40\right)\pi {{\it i}}}\end{array}\right)$$	$$\left(\begin{array}{c}0.49{e}^{\left(0.36\right)\pi {{\it i}}},\\ 0.43{e}^{\left(0.41\right)\pi {{\it i}}},\\ 0.43{e}^{\left(0.48\right)\pi {{\it i}}}\end{array}\right)$$	$$\left(\begin{array}{c}0.44{e}^{\left(0.31\right)\pi {{\it i}}},\\ 0.37{e}^{\left(0.43\right)\pi {{\it i}}},\\ 0.40{e}^{\left(0.39\right)\pi {{\it i}}}\end{array}\right)$$	$$\left(\begin{array}{c}0.44{e}^{\left(0.30\right)\pi {{\it i}}},\\ 0.42{e}^{\left(0.41\right)\pi {{\it i}}},\\ 0.43{e}^{\left(0.48\right)\pi {{\it i}}}\end{array}\right)$$
($${s}_{5}$$,$${s}_{2})$$	$$\left(\begin{array}{c}0.44{e}^{\left(0.29\right)\pi {{\it i}}},\\ 0.35{e}^{\left(0.30\right)\pi {{\it i}}},\\ 0.40{e}^{\left(0.40\right)\pi {{\it i}}}\end{array}\right)$$	$$\left(\begin{array}{c}0.48{e}^{\left(0.39\right)\pi {{\it i}}},\\ 0.39{e}^{\left(0.33\right)\pi {{\it i}}},\\ 0.41{e}^{\left(0.45\right)\pi {{\it i}}}\end{array}\right)$$	$$\left(\begin{array}{c}0.52{e}^{\left(0.41\right)\pi {{\it i}}},\\ 0.41{e}^{\left(0.33\right)\pi {{\it i}}},\\ 0.51{e}^{\left(0.48\right)\pi {{\it i}}}\end{array}\right)$$	$$\left(\begin{array}{c}0.51{e}^{\left(0.30\right)\pi {{\it i}}},\\ 0.35{e}^{\left(0.29\right)\pi {{\it i}}},\\ 0.43{e}^{\left(0.49\right)\pi {{\it i}}}\end{array}\right)$$	$$\left(\begin{array}{c}0.41{e}^{\left(0.31\right)\pi {{\it i}}},\\ 0.30{e}^{\left(0.43\right)\pi {{\it i}}},\\ 0.39{e}^{\left(0.41\right)\pi {{\it i}}}\end{array}\right)$$	$$\left(\begin{array}{c}0.49{e}^{\left(0.30\right)\pi {{\it i}}},\\ 0.42{e}^{\left(0.41\right)\pi {{\it i}}},\\ 0.43{e}^{\left(0.48\right)\pi {{\it i}}}\end{array}\right)$$
($${s}_{5}$$,$${s}_{3})$$	$$\left(\begin{array}{c}0.55{e}^{(0.34)\pi {{\it i}}},\\ 0.45{e}^{(0.39)\pi {{\it i}}},\\ 0.40{e}^{\left(0.40\right)\pi {{\it i}}}\end{array}\right)$$	$$\left(\begin{array}{c}0.51{e}^{\left(0.39\right)\pi {{\it i}}},\\ 0.39{e}^{\left(0.33\right)\pi {{\it i}}},\\ 0.41{e}^{\left(0.45\right)\pi {{\it i}}}\end{array}\right)$$	$$\left(\begin{array}{c}0.53{e}^{\left(0.41\right)\pi {{\it i}}},\\ 0.41{e}^{\left(0.39\right)\pi {{\it i}}},\\ 0.39{e}^{\left(0.40\right)\pi {{\it i}}}\end{array}\right)$$	$$\left(\begin{array}{c}0.54{e}^{\left(0.41\right)\pi {{\it i}}},\\ 0.35{e}^{\left(0.41\right)\pi {{\it i}}},\\ 0.45{e}^{\left(0.51\right)\pi {{\it i}}}\end{array}\right)$$	$$\left(\begin{array}{c}0.42{e}^{\left(0.31\right)\pi {{\it i}}},\\ 0.37{e}^{\left(0.43\right)\pi {{\it i}}},\\ 0.39{e}^{\left(0.42\right)\pi {{\it i}}}\end{array}\right)$$	$$\left(\begin{array}{c}0.47{e}^{\left(0.30\right)\pi {{\it i}}},\\ 0.42{e}^{\left(0.41\right)\pi {{\it i}}},\\ 0.44{e}^{\left(0.48\right)\pi {{\it i}}}\end{array}\right)$$
($${s}_{5}$$,$${s}_{4})$$	$$\left(\begin{array}{c}0.48{e}^{\left(0.31\right)\pi {{\it i}}},\\ 0.45{e}^{\left(0.23\right)\pi {{\it i}}},\\ 0.40{e}^{\left(0.40\right)\pi {{\it i}}}\end{array}\right)$$	$$\left(\begin{array}{c}0.49{e}^{\left(0.23\right)\pi {{\it i}}},\\ 0.39{e}^{\left(0.33\right)\pi {{\it i}}},\\ 0.41{e}^{\left(0.45\right)\pi {{\it i}}}\end{array}\right)$$	$$\left(\begin{array}{c}0.46{e}^{\left(0.34\right)\pi {{\it i}}},\\ 0.35{e}^{\left(0.39\right)\pi {{\it i}}},\\ 0.39{e}^{\left(0.40\right)\pi {{\it i}}}\end{array}\right)$$	$$\left(\begin{array}{c}0.49{e}^{\left(0.36\right)\pi {{\it i}}},\\ 0.35{e}^{\left(0.41\right)\pi {{\it i}}},\\ 0.43{e}^{\left(0.46\right)\pi {{\it i}}}\end{array}\right)$$	$$\left(\begin{array}{c}0.48{e}^{\left(0.31\right)\pi {{\it i}}},\\ 0.37{e}^{\left(0.43\right)\pi {{\it i}}},\\ 0.40{e}^{\left(0.45\right)\pi {{\it i}}}\end{array}\right)$$	$$\left(\begin{array}{c}0.46{e}^{\left(0.30\right)\pi {{\it i}}},\\ 0.42{e}^{\left(0.41\right)\pi {{\it i}}},\\ 0.43{e}^{\left(0.48\right)\pi {{\it i}}}\end{array}\right)$$
($${s}_{5}$$,$${s}_{5})$$	$$\left(\begin{array}{c}0.55{e}^{(0.34)\pi {{\it i}}},\\ 0.45{e}^{(0.39)\pi {{\it i}}},\\ 0.40{e}^{\left(0.40\right)\pi {{\it i}}}\end{array}\right)$$	$$\left(\begin{array}{c}0.52{e}^{\left(0.39\right)\pi {{\it i}}},\\ 0.39{e}^{\left(0.33\right)\pi {{\it i}}},\\ 0.41{e}^{\left(0.45\right)\pi {{\it i}}}\end{array}\right)$$	$$\left(\begin{array}{c}0.53{e}^{\left(0.45\right)\pi {{\it i}}},\\ 0.41{e}^{\left(0.46\right)\pi {{\it i}}},\\ 0.39{e}^{\left(0.40\right)\pi {{\it i}}}\end{array}\right)$$	$$\left(\begin{array}{c}0.54{e}^{\left(0.46\right)\pi {{\it i}}},\\ 0.51{e}^{\left(0.41\right)\pi {{\it i}}},\\ 0.43{e}^{\left(0.46\right)\pi {{\it i}}}\end{array}\right)$$	$$\left(\begin{array}{c}0.49{e}^{\left(0.31\right)\pi {{\it i}}},\\ 0.37{e}^{\left(0.43\right)\pi {{\it i}}},\\ 0.39{e}^{\left(0.39\right)\pi {{\it i}}}\end{array}\right)$$	$$\left(\begin{array}{c}0.51{e}^{\left(0.30\right)\pi {{\it i}}},\\ 0.42{e}^{\left(0.41\right)\pi {{\it i}}},\\ 0.43{e}^{\left(0.48\right)\pi {{\it i}}}\end{array}\right)$$

The CP of two CSFSSs is shown in the table above. Convert the complex values to real numbers to compute the score values. First of all, to convert the all exponential values to the form of $a+ib$. i.e., $a+ib={re}^{\pi {{\it i}}\theta }, as r=\sqrt{{a}^{2}+{b}^{2}}\text{ and }{ e}^{\pi {{\it i}}\theta }={\text{cos}}\left(\theta \right)+i{\text{sin}}(\theta )$, Then $a = r{\text{cos}}\pi (\theta ), b = {\text{sin}}\pi (\theta ).\pi$ show to as a cycle of the circle. Take modulus after converting polar form to standard form. These procedures apply to the membership, abstinence and non-membership. After all this process, apply to membership, abstinence and non-membership score formula to ${{(\psi }_{{\acute{\text{\AA}}}})}^{2}+{{(\mu }_{{\acute{\text{\AA}}}})}^{2}-{{(\eta }_{{\acute{\text{\AA}}}})}^{2}$, as shown in Table 3.

**Table 3:** Score value of web browser.

Ordered pair	${\check{\mathrm{u}}}_{1}$	${\check{\mathrm{u}}}_{2}$	${\check{\mathrm{u}}}_{3}$	${\check{\mathrm{u}}}_{4}$	${\check{\mathrm{u}}}_{5}$	$\lambda$
($${s}_{1}$$,$${s}_{1})$$	0.28171	0.37188	0.18931	0.24001	0.18596	0.20808
($${s}_{1}$$,$${s}_{2})$$	0.15897	0.23858	0.07363	0.17751	0.09826	0.18808
($${s}_{1}$$,$${s}_{3})$$	0.27288	0.31192	0.18931	0.15991	0.15628	0.17938
($${s}_{1}$$,$${s}_{4})$$	0.28171	0.31192	0.18931	0.15991	0.17816	0.20808
($${s}_{1}$$,$${s}_{5})$$	0.27288	0.22400	0.18161	0.24001	0.17056	0.20808
($${s}_{2}$$,$${s}_{1})$$	0.15897	0.23858	0.07363	0.17751	0.09826	0.18808
($${s}_{2}$$,$${s}_{2})$$	0.20039	0.24620	0.29109	0.19757	0.12128	0.24001
($${s}_{2}$$,$${s}_{3})$$	0.17919	0.23020	0.18959	0.18017	0.12128	0.21216
($${s}_{2}$$,$${s}_{4})$$	0.17919	0.23020	0.07363	0.15991	0.09826	0.21130
($${s}_{2}$$,$${s}_{5})$$	0.15617	0.21420	0.17829	0.19757	0.10598	0.23450
($${s}_{3}$$,$${s}_{1})$$	0.27288	0.31192	0.18931	0.15991	0.15628	0.17938
($${s}_{3}$$,$${s}_{2})$$	0.17919	0.23020	0.18959	0.18017	0.12128	0.21216
($${s}_{3}$$,$${s}_{3})$$	0.36818	0.20986	0.31588	0.21149	0.18716	0.22976
($${s}_{3}$$,$${s}_{4})$$	0.29590	0.31192	0.18931	0.15991	0.15628	0.20666
($${s}_{3}$$,$${s}_{5})$$	0.34506	0.24406	0.29688	0.21149	0.16408	0.20666
($${s}_{4}$$,$${s}_{1})$$	0.28171	0.31192	0.18931	0.15991	0.17816	0.20808
($${s}_{4}$$,$${s}_{2})$$	0.17919	0.23020	0.07363	0.15991	0.09826	0.21130
($${s}_{4}$$,$${s}_{3})$$	0.29590	0.31192	0.18931	0.15991	0.15628	0.20666
($${s}_{4}$$,$${s}_{4})$$	0.33342	0.31192	0.18931	0.15991	0.21478	0.26212
($${s}_{4}$$,$${s}_{5})$$	0.27288	0.22400	0.18161	0.17751	0.20718	0.20580
($${s}_{5}$$,$${s}_{1})$$	0.27288	0.22400	0.18161	0.24001	0.17056	0.20808
($${s}_{5}$$,$${s}_{2})$$	0.15617	0.21420	0.17829	0.19757	0.10598	0.23450
($${s}_{5}$$,$${s}_{3})$$	0.34506	0.24406	0.29688	0.21149	0.16408	0.20666
($${s}_{5}$$,$${s}_{4})$$	0.27288	0.22400	0.18161	0.17751	0.20718	0.20580
($${s}_{5}$$,$${s}_{5})$$	0.34506	0.25427	0.29688	0.36676	0.22470	0.25456

To find the best web browser, we must first get the highest numerical degree in each row while ignoring the last column. The last column is the general belongingness of each web browser parameter. Now, the desired value of $\lambda$ is added to the product of these numerical degrees to determine each web browser’s score. The highest scoring of web browser is the best. We do not examine the numerical degree of the same parametric ordered pair’s browser in our analysis because we believe that this measure is not unique and does not provide a useful foundation for comparison. The intricate structure of web browsers and the variety of features they offer make it impossible to assess them solely through numerical means. Rather, we shift our attention to a more thorough comprehension of the distinct qualities and performance indicators that add to the overall effectiveness of every browser.

In the midst of this strategy, we now focus on the following phase of our assessment procedure. We focus our efforts on calculating the score function as presented in Table 4 in order to more precisely assess the performance. This methodical approach enables a nuanced evaluation by taking into account a number of variables and elements that affect each web browser’s overall performance and usefulness. We hope to offer a more thorough and informative analysis that goes beyond simple numerical comparisons and explores the subtle nuances of each browser’s capabilities by utilizing a score function methodology.

**Table 4:** Grade table of the score function.

$${\raisebox{-4.5pt}{{\rm R}}\rotatebox{45}{\hspace*{-11pt}--}}$$	($${s}_{1}$$,$${s}_{1})$$	($${s}_{1}$$,$${s}_{2})$$	($${s}_{1}$$,$${s}_{3})$$	($${s}_{1}$$,$${s}_{4})$$	($${s}_{1}$$,$${s}_{5})$$	($${s}_{2}$$,$${s}_{1})$$	($${s}_{2}$$,$${s}_{2})$$	($${s}_{2}$$,$${s}_{3})$$	($${s}_{2}$$,$${s}_{4})$$
${\check{\mathrm{u}}}_{i}$	${\check{\mathrm{u}}}_{2}$	${\check{\mathrm{u}}}_{2}$	${\check{\mathrm{u}}}_{2}$	${\check{\mathrm{u}}}_{2}$	${\check{\mathrm{u}}}_{1}$	${\check{\mathrm{u}}}_{2}$	${\check{\mathrm{u}}}_{3}$	${\check{\mathrm{u}}}_{2}$	${\check{\mathrm{u}}}_{2}$
Highest degree	×	0.23858	0.31192	0.31192	0.27288	0.23858	×	0.23020	0.23020
$\lambda$	×	0.18808	0.17938	0.20808	0.20808	0.18808	×	0.21216	0.21130
$${\raisebox{-4.5pt}{{\rm R}}\rotatebox{45}{\hspace*{-11pt}--}}$$	($${s}_{2}$$,$${s}_{5})$$	($${s}_{3}$$,$${s}_{1})$$	($${s}_{3}$$,$${s}_{2})$$	($${s}_{3}$$,$${s}_{3})$$	($${s}_{3}$$,$${s}_{4})$$	($${s}_{3}$$,$${s}_{5})$$	($${s}_{4}$$,$${s}_{1})$$	($${s}_{4}$$,$${s}_{2})$$	($${s}_{4}$$,$${s}_{3})$$
${\check{\mathrm{u}}}_{i}$	${\check{\mathrm{u}}}_{2}$	${\check{\mathrm{u}}}_{2}$	${\check{\mathrm{u}}}_{2}$	${\check{\mathrm{u}}}_{1}$	${\check{\mathrm{u}}}_{2}$	${\check{\mathrm{u}}}_{1}$	${\check{\mathrm{u}}}_{2}$	${\check{\mathrm{u}}}_{2}$	${\check{\mathrm{u}}}_{2}$
Highest degree	0.21420	0.31192	0.23020	×	0.31192	0.34506	0.31192	0.23020	0.31120
$\lambda$	0.23450	0.17938	0.21216	×	0.20666	0.20666	0.20808	0.21130	0.20666
$${\raisebox{-4.5pt}{{\rm R}}\rotatebox{45}{\hspace*{-11pt}--}}$$	($${s}_{4}$$,$${s}_{4})$$	($${s}_{4}$$,$${s}_{5})$$	($${s}_{5}$$,$${s}_{1})$$	($${s}_{5}$$,$${s}_{2})$$	($${s}_{5}$$,$${s}_{3})$$	($${s}_{5}$$,$${s}_{4})$$	($${s}_{5}$$,$${s}_{5})$$		
${\check{\mathrm{u}}}_{i}$	${\check{\mathrm{u}}}_{1}$	${\check{\mathrm{u}}}_{1}$	${\check{\mathrm{u}}}_{1}$	${\check{\mathrm{u}}}_{2}$	${\check{\mathrm{u}}}_{1}$	${\check{\mathrm{u}}}_{1}$	${\check{\mathrm{u}}}_{4}$		
Highest degree	×	0.27288	0.27288	0.21420	0.34506	0.27288	×		
$\lambda$	×	0.20580	0.20808	0.23450	0.20666	0.20580	×		

$${S({{\check{\mathrm{u}}}}}_{1})=\left(0.27288\times 0.20808\right)+\left(0.34506\times 0.20666\right)+\left(0.27288\times 0.20580\right)+\left(0.27288\times 0.20808\right)+\left(0.34506\times 0.20666\right)+\left(0.27288\times 0.20580\right)=0.36974,$$ $${S({{\check{\mathrm{u}}}}}_{2})=(0.23858\times 0.18808)+(0.31192\times 0.17938)+(0.31192\times 0.20808)+(0.23858\times 0.18808)+ (0.23020\times 0.21216)+(0.23020\times 0.21130)+(0.21420\times 0.23450)+(0.31192\times 0.17938)+(0.23020\times 0.21216)+(0.31192\times 0.20666)+(0.31192\times 0.20808)+(0.23020\times 0.21130)+(0.31120\times 0.20666)+(0.21420\times 0.23450)=0.75565.$$

When compared to other web browsers, Google Chrome has a reputation for being incredibly efficient, and users have acknowledged its exponential performance advantages. This browser’s unmatched efficiency can be attributed to its simplified features, quick page loads, and resource optimization. Its attractiveness is further increased by the easy-to-use UI and flawless device syncing. Google Chrome’s reputation as a dependable and trustworthy option is further strengthened by its dedication to security and regular updates. When compared to other browsers, Google Chrome performs better in terms of speed, stability, and overall usability. Users can enjoy a customized and effective browsing experience thanks to its extensive customization options and extensive extension library. Google Chrome has established itself as the leading web browser by continuously pushing the envelope of innovation and establishing the bar for other individuals to exceed.

### Comparative analysis

In this section, the concepts of the CSFSRs are compared with some pre-existing structures in the theory of FSS such as SRs, FSRs, CFSRs, IFSRs, and CIFSRs. A mapping from a parameterized family to the crisp subset is called a soft relation. Rather than defining yes or no, the crisp relation only defines 1 or 0. As a result, the limited knowledge is indicated by a sharp relation. A fuzzy number called the degree of membership defines a set called an FSS. A related relation is referred to as an FSR. FSRs are the only membership level that is discussed. In an ordered pair, the fuzzy soft relations indicating only the effectiveness of first parameter over the second. The fuzzy soft relations are the single dimension and give the limited information. The CFSSs are described by the complex fuzzy number, and corresponding relations are called a CFSRs. The CFSRs are discussed only the membership degree with complex number. The CFSRs are mainly two parts i.e. amplitude term and phase term. An amplitude term characterized the strength of the different web browser and the phase term is used to describe the time period over the certain conditions. A IFSSs are characterized the membership and non-membership degree. The corresponding relations are known as IFSRs. In an ordered pair the IFSRs show the effectiveness and ineffectiveness of the first parameter over the second. A IFSRs lack of ability to represent such problems that include time they are provided incomplete information. The degree of membership, abstinence, and non-membership in an SFSS is characterized. SFSRs are the corresponding relations. The SFSRs display each parameter’s effectiveness, ineffectiveness, and lack of effect. The CSFSRs are discussed the membership, abstinence, and non-membership with complex number. They are discussing the both amplitude term and phase term. So, it gives about complete information of any problem.

### Comparison of FSRs, CFSRs, CPyFSRs, with CSFSRs

A fundamental idea that unifies fuzzy logic and soft set theory, FSRs handle uncertainty by utilizing membership values in the [0, 1] range and linguistic terms to model uncertainty. On the other hand, CFSRs enhance the representation in complex systems by incorporating complex numbers. By extending PyFSs and utilizing complex numbers and Pythagorean triples, CPyFSRs present a methodical approach. By introducing SFS with complex numbers, CSFSRs further advance the paradigm and provide a comprehensive model for multidimensional uncertainty.

FSRs are a simple yet effective tool for comparing these models, whereas CFSRs offer more representational power. When triangular membership functions are advantageous, CPyFSRs perform well; similarly, CSFSRs perform exceptionally well in situations involving spherical uncertainty structures. The complexity of the problem domain determines which of these models is best, with computational complexity rising as models include more complex components. This highlights the need of making a thoughtful selection that is in line with the nature of uncertainty in decision-making processes.

### Comparison of IFSRs, and CIFSRs, with CSFSRs

Combining the ideas of FS theory and SS theory, IFSRs serve as the cornerstone of uncertainty modelling by offering a flexible tool for managing ambiguity and imprecision in a variety of contexts. On the other hand, by adding complex numbers, CIFSRs improve this paradigm and add a level of sophistication to the representation of uncertainty in complex systems. Because of their enhanced framework, CIFSRs allow for a more detailed modelling of relationships and uncertainties, especially in situations where traditional IFSRs might not be sufficient. Conversely, CSFSRs expand the representation of multidimensional uncertainty by introducing SFS with complex numbers, which is an innovative approach. In order to compare these models, one must assess how well-suited each is for various uncertainty structures, computational complexity, and representation richness. The choice depends on the particulars of the situation at hand and the complexities of the uncertainties involved. While IFSRs offer a strong foundation, CIFSRs and CSFSRs offer advanced features catering to complex and multidimensional uncertainties. In order to make well-informed decisions that are in line with the intricacies of their applications, researchers and practitioners need to carefully consider the distinctive qualities of each model.

### Comparison of PFSRs, and CPFSRs, with CSFSRs

PFSRs combine the visual richness of PFS with FS theory and SS theory to form a potent framework for managing uncertainty. By utilizing the graphical depiction of membership degrees, PFSRs offer a natural way to simulate uncertainty and imprecision in scenarios involving decision-making. However, by adding complex numbers, CPFSRs improve this paradigm and add another level of complexity to the capture of uncertainties in complex systems. When complex numbers are integrated, uncertainties can be represented more nuanced in the visual domain, providing a sophisticated tool for scenarios involving complex decision-making where standard PFSRs might not be adequate.

A different approach, called CSFSRs, extends the representation of uncertainties to a multidimensional spherical space by introducing SFS with complex numbers. In the comparison, the expressiveness of the visuals, computational efficiency, and fit for multidimensional, complex uncertainties are evaluated for each of these models. Although PFSRs provide a graphical approach that is easy to understand, CPFSRs and CSFSRs are designed for more complex scenarios and have different advantages and disadvantages. The type of uncertainties in a given problem domain determines which of these models to use, highlighting the importance of making an informed decision that is in line with the specifics of the decision-making environment.

### Comparison on the basis of structure

Table 5 provides a thorough and comprehensive summary of the conclusions drawn from the extensive comparative analysis carried out on Complex Spherical Fuzzy Soft Relations (CSFSRs) in comparison with a predetermined structure. This thorough investigation offers a nuanced understanding of the different aspects and complexities of CSFSRs, providing a comprehensive and rich comparative analysis that clarifies their qualities, applicability, and performance when compared to a predetermined structural framework. In addition to highlighting the unique characteristics of CSFSRs, the tabulated data is a useful tool for understanding how these relations perform relative to the established structure that is being examined. Thus, the comparative overview shown in Table 5 is an essential tool for scholars, practitioners, and interested parties who want to learn more about the nuances of CSFSRs and how they relate to predefined structures.

**Table 5:** Comparative analysis on the basis of structure.

Structure	Membership	Abstinence	Non-membership	Multi-dimension	Space
SR	No	No	No	No	$n=1$
IFR	Yes	No	Yes	No	$n=1$
FSR	Yes	No	No	No	$n=1$
CFSR	Yes	No	No	Yes	$n=1$
PyFR	Yes	No	Yes	No	$n=2$
IFSR	Yes	No	Yes	No	$n=1$
CIFSR	Yes	No	Yes	Yes	$n=1$
PFSR	Yes	Yes	Yes	No	$n=1$
CSFSR	Yes	Yes	Yes	Yes	$n=2$

## Conclusion

In this paper, introduced the new concept of CSFSRs and their types have been discussed such as CSFS converse relation, CSFS reflexive relation, CSFS irreflexive relation, CSFS symmetric relation, CSFS anti-symmetric relation, CSFS asymmetric relation, CSFS complete relation, CSFS transitive relation, CSFS equivalence relation, CSFS partial order relation, CSFS strict order relation, CSFS preorder relation and CSFS equivalence classes. Some outcomes proved with appropriate example. In addition, the CP of two CSFSS is also described. Moreover, this novel concept of CSFSRs is used an application of web browser. The goal of this application is to find the most effective web browser. The web browser represents the different parameters. The expert gives the membership, abstinence, and non-membership values of each web browser parameters.

Then, using score function, they choose the best web browser based on a set of parameters. Score function is used in this article to choose a best object or anything based on some parameters. To sum up, our investigation into a fresh perspective on web browsers using the ground-breaking idea of Complex Spherical Fuzzy Soft Information reveals an exciting new direction in the field of online user experiences. Our proposed framework presents a more adaptive and nuanced interpretation of user preferences, while also reinventing traditional browser functionalities through the integration of fuzzy logic, spherical geometry, and complexity theory. This work represents a major departure from the status quo by highlighting the significance of utilizing sophisticated mathematical models to improve the intelligence and sophistication of web browsers. Finally, the CSFSRs are preferable to the pre-determined structure. The CSFSRs have the ability to solve periodicity. These ideas can be used in future to further generalize FSS, resulting in a novel framework that can be useful to a variety of fields.

## Data Availability

The data used to support the findings of this study are available from the corresponding author upon request.

## References

[1] Zadeh LA (1965). Fuzzy sets. Inf. Control.

[2] Zimmermann HJ (2011). Fuzzy Set Theory—And Its Applications.

[3] Roberts DW (1986). Ordination on the basis of fuzzy set theory. Vegetatio.

[4] Maiers J, Sherif YS (1985). Applications of fuzzy set theory. IEEE Trans. Syst. Man Cybern..

[5] Mendel JM (1995). Fuzzy logic systems for engineering: A tutorial. Proc. IEEE.

[6] Yang MS, Shih HM (2001). Cluster analysis based on fuzzy relations. Fuzzy Sets Syst..

[7] Ramot D, Milo R, Friedman M, Kandel A (2002). Complex fuzzy sets. IEEE Trans. Fuzzy Syst..

[8] Li C, Tu CH (2019). Complex neural fuzzy system and its application on multi-class prediction—A novel approach using complex fuzzy sets, IIM and multi-swarm learning. Appl. Soft Comput..

[9] Yazdanbakhsh O, Dick S (2018). A systematic review of complex fuzzy sets and logic. Fuzzy Sets Syst..

[10] Khan M, Zeeshan M, Song SZ, Iqbal S (2021). Types of complex fuzzy relations with applications in future commission market. J. Math..

[11] Molodtsov D (1999). Soft set theory—First results. Comput. Math. Appl..

[12] Ali MI, Feng F, Liu X, Min WK, Shabir M (2009). On some new operations in soft set theory. Comput. Math. Appl..

[13] Yang CF (2008). A note on “soft set theory”. Comput. Math. Appl..

[14] Babitha KV, Sunil J (2010). Soft set relations and functions. Comput. Math. Appl..

[15] Maji PK, Roy AR, Biswas R (2002). An application of soft sets in a decision making problem. Comput. Math. Appl..

[16] Georgiou DN, Megaritis AC (2014). Soft set theory and topology. Appl. Gen. Topol..

[17] Park JH, Kim OH, Kwun YC (2012). Some properties of equivalence soft set relations. Comput. Math. Appl..

[18] Maji, P. K., Biswas, R. K. & Roy, A. *Fuzzy Soft Sets* (2001).

[19] Gogoi K, Dutta AK, Chutia C (2014). Application of fuzzy soft set theory in day to day problems. Int. J. Comput. Appl..

[20] Kong Z, Wang L, Wu Z (2011). Application of fuzzy soft set in decision making problems based on grey theory. J. Comput. Appl. Math..

[21] Borah MJ, Neog TJ, Sut DK (2012). Relations on fuzzy soft sets. J. Math. Comput. Sci..

[22] Hayat K, Ali MI, Karaaslan F, Cao BY, Shah MH (2020). Design concept evaluation using soft sets based on acceptable and satisfactory levels: An integrated TOPSIS and Shannon entropy. Soft Comput..

[23] Hayat K, Raja MS, Lughofer E, Yaqoob N (2023). New group-based generalized interval-valued q-rung orthopair fuzzy soft aggregation operators and their applications in sports decision-making problems. Comput. Appl. Math..

[24] Rehman UU, Mahmood T, Albaity M, Hayat K, Ali Z (2022). Identification and prioritization of DevOps success factors using bipolar complex fuzzy setting with Frank aggregation operators and analytical hierarchy process. IEEE Access.

[25] Yang X, Hayat K, Raja MS, Yaqoob N, Jana C (2022). Aggregation and interaction aggregation soft operators on interval-valued q-rung orthopair fuzzy soft environment and application in automation company evaluation. IEEE Access.

[26] Thirunavukarasu P, Suresh R, Ashokkumar V (2017). Theory of CFS set and its applications. Int. J. Innov. Res. Sci. Technol..

[27] Tamir, D. E., Rishe, N. D. & Kandel, A. Complex fuzzy sets and complex fuzzy logic an overview of theory and applications. In *Fifty Years of Fuzzy Logic and Its Applications* 661–681 (2015).

[28] Atanassov KT (1986). Intuitionistic fuzzy sets. Fuzzy Sets Syst..

[29] Gerstenkorn T, Mańko J (1991). Correlation of intuitionistic fuzzy sets. Fuzzy Sets Syst..

[30] Alkouri, A. M. D. J. S. & Salleh, A. R. Complex intuitionistic fuzzy sets. In *AIP Conference Proceedings*, Vol. 1482, 464–470 (American Institute of Physics, 2012).

[31] Xu, Y. J., Sun, Y. K. & Li, D. F. Intuitionistic fuzzy soft set. In *2010 2nd International Workshop on Intelligent Systems and Applications* 1–4 (IEEE, 2010).

[32] Agarwal M, Biswas KK, Hanmandlu M (2013). Generalized intuitionistic fuzzy soft sets with applications in decision-making. Appl. Soft Comput..

[33] Dinda, B. & Samanta, T. K. Relations on intuitionistic fuzzy soft sets. Preprint at http://arXiv.org/1202.4649 (2012).

[34] Kumar T, Bajaj RK (2014). On complex intuitionistic fuzzy soft sets with distance measures and entropies. J. Math..

[35] Akram M, Wasim F, Karaaslan F (2021). MCGDM with complex Pythagorean fuzzy-soft model. Expert Syst..

[36] Peng XD, Yang Y, Song J, Jiang Y (2015). Pythagorean fuzzy soft set and its application. Comput. Eng..

[37] Cuong BC, Kreinovich V (2014). Picture fuzzy sets. J. Comput. Sci. Cybern..

[38] Thong PH, Son LH, Nguyen V-H, Le A-C, Huynh V-N (2015). A new approach to multi-variable fuzzy forecasting using picture fuzzy clustering and picture fuzzy rule interpolation method. Knowledge and Systems Engineering.

[39] Kutlu GF, Kahraman C (2019). Spherical fuzzy sets and spherical fuzzy TOPSIS method. J. Intell. Fuzzy Syst..

[40] Jan N, Mahmood T, Zedam L, Ali Z (2020). Multi-valued picture fuzzy soft sets and their applications in group decision-making problems. Soft Comput..

[41] Shanthi SA, Umamakeswari T, Saranya M (2022). MCDM method on complex picture fuzzy soft environment. Mater. Today Proc..

[42] Akram M, Shabir M, Adeel A, Al-Kenani AN (2021). A multi attribute decision-making framework: VIKOR method with complex spherical fuzzy-soft sets. Math. Probl. Eng..

[43] Akram M, Shabir M, Al-Kenani AN, Alcantud JCR (2021). Hybrid decision-making frameworks under complex spherical fuzzy-soft sets. J. Math..

